# Supplementary material for: GRAPE for fast and scalable graph processing and random-walk-based embedding
Source: Nat Comput Sci. 2023 Jun 26;3(6):552–68. doi: 10.1038/s43588-023-00465-8 (PMC10768636; doi:10.1038/s43588-023-00465-8)
Supplement: Supplementary file 1 — Supplementary Figs. 1–37, Tables 1–51, Sections 1–10. [file 43588_2023_465_MOESM1_ESM.pdf]

nature computational science

Resource https://doi.org/10.1038/s43588-023-00465-8

GRAPE for fast and scalable graph processing and random-walk-based embedding

In the format provided by the authors and unedited

Contents

1 Related work **3**
- 1.1 NetworkX . . . 3
- 1.2 GraphEmbedding . . . 3
- 1.3 Node2Vec . . . 3
- 1.4 iGraph . . . 3
- 1.5 CSRGraph . . . 3
- 1.6 PecanPy . . . 3
- 1.7 FastNode2Vec . . . 4

2 Graphs used for *Ensmallen* benchmarks **5**

3 Graphs used for edge & node-label prediction experiments **6**
- 3.1 Datasets/Graphs directly accessible from *GRAPE* . . . 6
- 3.2 Graphs used for the edge-prediction experiments . . . 6
  - 3.2.1 HomoSapiens . . . 6
    - Degree centrality . . . 6
    - Weights . . . 6
    - Topological Oddities . . . 6
  - 3.2.2 MusMusculus . . . 10
    - Degree centrality . . . 10
    - Weights . . . 10
    - Topological Oddities . . . 10
  - 3.2.3 Homo Phenotype Ontology . . . 15
    - Degree centrality . . . 15
    - Node types . . . 15
    - Edge types . . . 15
    - Topological Oddities . . . 15
- 3.3 Graphs used for node-label prediction experiments . . . 19
  - 3.3.1 Cora . . . 19
    - Degree centrality . . . 19
    - Node types . . . 19
    - Topological Oddities . . . 20
  - 3.3.2 CiteSeer . . . 23
    - Degree centrality . . . 23
    - Node types . . . 24
    - Topological Oddities . . . 24
  - 3.3.3 Cora . . . 29
    - Degree centrality . . . 30
    - Node types . . . 30
    - Topological Oddities . . . 30

4 Models used in edge & node-label prediction experiments **34**
- 4.1 Evaluated node embedding models . . . 34
  - 4.1.1 Node2Vec CBOW . . . 34
  - 4.1.2 Node2Vec GloVe . . . 34
  - 4.1.3 Node2Vec SkipGram . . . 34
  - 4.1.4 DeepWalk CBOW . . . 34
  - 4.1.5 DeepWalk GloVe . . . 35
  - 4.1.6 DeepWalk SkipGram . . . 35
  - 4.1.7 First Order LINE . . . 35
  - 4.1.8 Second Order LINE . . . 35
  - 4.1.9 NMFADMM . . . 35
  - 4.1.10 RandNE . . . 35

4.1.11 GraRep . . . 37
4.1.12 DeepWalk Walklets SkipGram . . . 37
4.1.13 NetMF . . . 37
4.1.14 GLEE . . . 37
4.1.15 HOPE . . . 37
4.1.16 Role2Vec . . . 37
4.2 Evaluated edge prediction models . . . 38
4.2.1 Decision Tree Classifier . . . 38
4.2.2 Perceptron . . . 38
4.3 Evaluated node-label prediction models . . . 38
4.3.1 Decision Tree Classifier . . . 38
4.3.2 Random Forest Classifier . . . 38

5 Edge and node label prediction performance **39**
5.1 Edge prediction performance . . . 39
5.2 Node-label prediction performance . . . 48

6 Large real-world graph experimental results and big graphs used in the experiments. **56**
6.1 Big graphs description and construction . . . 56
English Wikipedia. . . . 56
Comparative Toxicogenomic Database (CTD). . . . 56
PheKnowLator biomedical data. . . . 56
6.2 State of the art graph libraries compared with *GRAPE* . . . 57
6.3 Evaluation of the results . . . 58
6.4 Additional experimental results with big graphs . . . 58

7 Ensmallen **64**
7.1 Elias-Fano scheme . . . 64
7.1.1 Edge encoding . . . 68
7.2 Specialized Random-Walks . . . 69
7.2.1 Faster Pseudo-Random Numbers Generators (Vectorized xorshift) . . . 71
7.2.2 Efficient cumulative sum computation . . . 73
7.2.3 Alias method . . . 75
7.2.4 SUSS: Sorted Unique Sub-Sampling . . . 76
7.3 Methods for accurately measuring RAM peak and time requirements . . . 76

8 Overview of techniques implemented in *GRAPE* **78**
8.1 Spectral and matrix factorization embedding methods . . . 78
8.2 RW-based embedding methods . . . 78
8.3 Triple-sampling methods. . . . 79
8.4 Corrupted triple-sampling methods. . . . 79

9 Visualization of Kipf GCN Models **80**

10 Other *GRAPE* utilities. **81**
10.1 Graph analysis and graph reporting . . . 81
10.2 Construction of train and test graphs for edge prediction . . . 81
10.2.1 K-folds . . . 81
10.2.2 Monte Carlo . . . 81
10.2.3 Connected Monte Carlo . . . 81
10.2.4 Negative edge sampling . . . 82

1 Related work

The node/edge embedding problem has been approached multiple times in the literature. In this section we briefly recall renowned packages that are mostly related to *GRAPE*, most of which have been used in our experiments as benchmarks for comparison.

None of the libraries identified in the literature provide an implementation of either a Graph-based CBOW or SkipGram, but rely on Gensim [1] CBOW and SkipGram Word2Vec model for the embedding procedure. The most performing libraries considered use Numba [2] just-in-time compilation to achieve better run-time executions, but such a dependency and related ecosystem (e.g. llvmlite) can be very complex to properly install e prone to signicant breaking changes between versions (e.g. between version '0.4' and '0.5').

1.1 NetworkX

NetworkX [3] is an extremely renowned Python language package for the exploration and analysis of networks. Even though this library does not provide fast first/second-order random walks, we includes it in our comparisons since it it used to handle the graph structure in the GraphEmbedding and Node2Vec packages mentioned below.

1.2 GraphEmbedding

GraphEmbedding [4] is a Python package for embedding networks via random-walk based methods such as Node2Vec. The graph is loaded using NetworkX. Within this package, the random walks are executed using the alias method [5] (see Supplementary Section 33), whose complexity during the preprocessing phase hampers the computation of random-walks to large graphs.To our knowledge, GraphEmbedding handles only undirected homogeneous graphs. The library relies on Gensim [1] for the embedding procedure.

1.3 Node2Vec

Node2Vec is a Python language package for embedding networks via random-walk based methods such as Node2Vec [6]. The graph is loaded using NetworkX. As mentioned for GraphEmbedding, also Node2Vec makes is limited by the usage of the alias method to execute the random walks. To our knowledge Node2Vec handles only undirected homogeneous graphs. The library relies on Gensim [1] for the embedding procedure.

1.4 iGraph

iGraph [7] is a library collection for creating and manipulating graphs and analyzing networks. While it was originally written in C language, some implementations are presently available as Python and R packages. We didn't use this library in our comparisons because it does not currently implement fast parallel first or second-order random walks, nor it does support their easy implementation. The iGraph library is not equipped with node embedding methods with the exception of two baseline spectral Laplacian methods.

1.5 CSRGraph

CSRGraph[8] is a library to execute fast first and second-order random walks using Numba [2]. Different from the previously mentioned libraries (e.g., GraphEmbedding or Node2Vec) CSRGraph doesn't store the graph into a NetworkX object, but instead exploits a compressed sparse row (CSR) matrix, which significantly reduces the memory requirements with respect to methods expliting NetworkX objects. Additionally, instead of using the aforementioned alias method, it computes the node probabilities and samples them lazily, as it is required. To our knowledge, this library only handles undirected homogeneous graphs. The library relies on Gensim [1] for the embedding procedure.

1.6 PecanPy

PecanPy [9] is a library to execute fast first and second-order random walks based on a set of different solutions depending on the graph densities and node number. For graphs with less than 10000 nodes,

PecanPy uses the alias-method, while for larger graphs it uses a strategy similar to CSR libray, where node probabilities and sampling are lazily computed and the CSR data structure is used to store the edges. Finally, for graphs with edge densities larger than 10% it switches the graph data structure to a dense adjacency matrix. Analogously to CSRGraph, also PecanPy makes extensive use of Numba [2]. The library relies on Gensim [1] for the embedding procedure.

1.7 FastNode2Vec

FastNode2Vec (`https://github.com/louisabraham/fastnode2vec`) is a library to *lazily* execute fast first and second-order random walks. Analogously to CSRGraph and PecanPy, also FastNode2CVec makes extensive use of Numba [2] and relies on Gensim [1] for the embedding procedure. The significant difference between FastNode2Vec and the previously mentioned libraries, namely PecanPy and CSRGraph, is that the random walks are computed lazily and directly fed into the Gensim model with a small amount of overhead and therefore it avoids the memory peak related to the rasterization of the random walks. This solution is very much analogous to what is done in GraPE, where the random walks are also computed lazily and fed into either the SkipGram or CBOW Rust models.

2 Graphs used for *Ensmallen* benchmarks

This section presents the main features of the graphs used in previous publications and used here to test *Ensmallen* vs. state-of-the-art graph libraries. 44 graphs from Network Repository [10], having a considerably different number of nodes and edges, have been collected as benchmark for comparison; the other graphs are GiantTN provided by Zenodo, Homo Sapiens being provided by STRING and KGCOVID19 provided by KGHub. Their main features are summarized in Supplementary Table 1.

Supplementary Table 1: Main features of the graphs considered as benchmark for comparison with *Ensmallen*.

Name	Nodes	Edges	Min degree	Max degree	Weights
HomoSapiens [11]	19566	11938498	0	7507	true
SocFlickr [10, 12]	513969	6380904	1	4369	false
SocFriendster [10]	65608366	3612134270	1	5214	false
SocBlogcatalog [10]	88784	4186390	1	9444	false
KGCOVID19 [13]	574215	36500884	0	122238	false
IMDB [10, 14]	896305	7564901	1	1590	false
BNHumanJung [10, 15]	975930	292218600	1	8009	false
BNFlyDrosophilaMedulla [10, 15]	1781	17927	1	927	false
BNMouseRetina [10, 15]	1076	181622	1	744	false
BNMacaqueRhesusBrain [10, 15]	242	6108	1	111	false
BioCeCx [10, 16]	15229	491904	1	375	true
BioCeGn [10, 16]	2220	107366	1	242	true
BioCeGt [10, 16]	924	6478	1	151	true
BioCeHt [10, 16]	2617	5970	1	44	true
BioCeLc [10, 16]	1387	3296	1	131	true
BioCePg [10, 16]	1871	95508	1	913	true
BioDmCx [10, 16]	4040	153434	1	362	true
BioDmHt [10, 16]	2989	9320	1	37	true
BioDmLc [10, 16]	658	2258	1	50	true
BioDmela [10, 16]	7393	51138	1	190	false
BioDrCx [10, 16]	3289	169880	1	497	true
BioHsCx [10, 16]	4413	217636	1	473	true
BioHsHt [10, 16]	2570	27382	1	149	true
BioHsLc [10, 16]	4227	78968	1	397	true
BioScCc [10, 16]	2223	69758	1	571	true
BioScGt [10, 16]	1716	67974	1	549	true
BioScHt [10, 16]	2084	126054	1	472	true
BioScLc [10, 16]	2004	40904	1	167	true
BioScTs [10, 16]	636	7918	1	66	true
BioCelegansDir [10, 17]	453	4065	1	238	false
BioCelegans [10, 17]	453	4050	1	237	false
BioDiseasome [10, 18]	516	2376	1	50	false
BioDmela [10, 19]	7393	51138	1	190	false
BioGridFissionYeast [10, 20]	2026	25274	1	439	false
BioGridFruitfly [10, 20]	7274	49788	1	176	false
BioGridHuman [10, 20]	9436	62364	1	308	false
BioGridMouse [10, 20]	1450	3272	1	111	false
BioGridPlant [10, 20]	1717	6196	1	71	false
BioGridWorm [10, 20]	3507	13062	1	523	false
BioGridYeast [10, 20]	6008	313890	1	2557	false
BioHumanGene1 [10, 21]	22283	24669643	1	7939	true
BioHumanGene2 [10, 21]	14340	18068388	1	7229	true
BioMouseGene [10, 21]	45101	28967291	1	8032	true
BioYeastProteinInter [10, 22]	1870	4480	1	56	false
BioYeast [10, 22]	1458	3896	1	56	false
GiantTN [?, 23]	25689	77809858	1	12384	true
WebWikipedia2009 [10]	1864433	9014630	1	2624	false

3 Graphs used for edge & node-label prediction experiments

3.1 Datasets/Graphs directly accessible from *GRAPE*

Other than simply loading an arbitrary graph from tabular documents with the node and edge lists, *GRAPE* allows the retrieval of all the graphs included into the STRING repository [11] (56691 graphs), KGHub and KGOBO [13], Monarch Initiative [24], Linqs [25], PheKnowLator [26], and over 1000 graphs from Network Repository [10]. Once a graph is loaded, the library can compute an extensive HTML human readable report simply by displaying the object, which within the context of a Jupyter Notebooks results in a well formatted report. Further tooling is made available to convert the report in LaTeX. The reports made available in Supplementary Sections 3.2 and 3.3 were obtained in this fashion. The automatically generated reports include general statistics about the graph, as well as data about singletons, node tuples, possible topological oddities, isomorphic node groups, trees, dendritic trees, stars and dendritic stars and tendrils eventually present in the graph.

3.2 Graphs used for the edge-prediction experiments

For the edge prediction experiments the STRING's Homo Sapiens, STRING's Mus Musculus, and Human Phenotype Ontology graphs have been used. To describe them in the following we copied the graph reports computed by *Ensmallen*.

3.2.1 HomoSapiens

The undirected graph HomoSapiens has 19.57K nodes and 252.98K edges. The graph contains 2.85K connected components (of which 2.75K are disconnected nodes), with the largest one containing 16.58K nodes and the smallest one containing a single node. The RAM requirements for the nodes and edges data structures are 2.67MB and 782.89KB respectively.

Degree centrality The minimum node degree is 0, the maximum node degree is 747, the mode degree is 0, the mean degree is 25.86 and the node degree median is 9. The nodes with the highest degree centrality are ENSP00000272317 (degree 747), ENSP00000269305 (degree 723), ENSP00000388107 (degree 669), ENSP00000441543 (degree 620) and ENSP00000362680 (degree 572).

Weights The minimum edge weight is 700, the maximum edge weight is 999 and the total edge weight is 437919420. The RAM requirement for the edge weights data structure is 2.02MB.

Topological Oddities A topological oddity is a set of nodes in the graph that *may be derived* by an error during the generation of the edge list of the graph and, depending on the task, could bias the results of topology-based models. In the following paragraph, we will describe the detected topological oddities.

Singleton nodes A singleton node is a node disconnected from all other nodes. We have detected 2.75K singleton nodes in the graph, involving a total of 2.75K nodes (14.07%). The detected singleton nodes are:

- ENSP00000005995
- ENSP00000006526
- ENSP00000006724
- ENSP00000007735
- ENSP00000010299
- ENSP00000039989
- ENSP00000064571
- ENSP00000084798
- ENSP00000086933
- ENSP00000158009
- ENSP00000159087
- ENSP00000161006
- ENSP00000162391
- ENSP00000164640
- ENSP00000190165

And other 2.74K singleton nodes.

Node tuples A node tuple is a connected component composed of two nodes. We have detected 77 node tuples in the graph, involving a total of 154 nodes (0.79%) and 77 edges (0.02%). The detected node tuples are:

- Node tuple containing the nodes ENSP00000484403 and ENSP00000484443.
- Node tuple containing the nodes ENSP00000480336 and ENSP00000485424.
- Node tuple containing the nodes ENSP00000454340 and ENSP00000485142.
- Node tuple containing the nodes ENSP00000448841 and ENSP00000481258.
- Node tuple containing the nodes ENSP00000436580 and ENSP00000436891.
- Node tuple containing the nodes ENSP00000436042 and ENSP00000436426.
- Node tuple containing the nodes ENSP00000435550 and ENSP00000441497.
- Node tuple containing the nodes ENSP00000430100 and ENSP00000454770.
- Node tuple containing the nodes ENSP00000429808 and ENSP00000433378.
- Node tuple containing the nodes ENSP00000419502 and ENSP00000483005.
- Node tuple containing the nodes ENSP00000406723 and ENSP00000480524.
- Node tuple containing the nodes ENSP00000399664 and ENSP00000472698.
- Node tuple containing the nodes ENSP00000391594 and ENSP00000460602.
- Node tuple containing the nodes ENSP00000388864 and ENSP00000396732.
- Node tuple containing the nodes ENSP00000384214 and ENSP00000397103.

And other 62 node tuples.

Isomorphic node groups Isomorphic groups are nodes with exactly the same neighbours and node types (if present in the graph). Nodes in such groups are topologically indistinguishable, that is swapping their ID would not change the graph topology. We have detected 21 isomorphic node groups in the graph, involving a total of 46 nodes (0.24%) and 558 edges (0.11%), with the largest one involving 5 nodes and 95 edges. The detected isomorphic node groups, sorted by decreasing size, are:

- Group with 5 nodes (degree 19): ENSP00000380488, ENSP00000423313, ENSP00000227756, ENSP00000344260 and ENSP00000297107.
- Group with 2 nodes (degree 29): ENSP00000391692 and ENSP00000391869.
- Group with 2 nodes (degree 21): ENSP00000436604 and ENSP00000444171.
- Group with 2 nodes (degree 18): ENSP00000384876 and ENSP00000373278.
- Group with 2 nodes (degree 18): ENSP00000347119 and ENSP00000354723.
- Group with 3 nodes (degree 11): ENSP00000369564, ENSP00000369566 and ENSP00000239347.
- Group with 2 nodes (degree 15): ENSP00000416741 and ENSP00000264363.
- Group with 2 nodes (degree 15): ENSP00000400157 and ENSP00000312700.
- Group with 2 nodes (degree 15): ENSP00000261196 and ENSP00000441269.
- Group with 2 nodes (degree 12): ENSP00000334681 and ENSP00000347810.
- Group with 2 nodes (degree 10): ENSP00000335281 and ENSP00000334330.
- Group with 2 nodes (degree 10): ENSP00000335307 and ENSP00000334364.
- Group with 2 nodes (degree 9): ENSP00000449334 and ENSP00000449223.

- Group with 2 nodes (degree 7): ENSP00000259205 and ENSP00000259211.
- Group with 2 nodes (degree 6): ENSP00000376955 and ENSP00000275884.

And other 6 isomorphic node groups.

Trees A tree is a connected component with n nodes and n-1 edges. We have detected 2 trees in the graph, involving a total of 14 nodes (0.07%) and 12 edges, with the largest one involving 9 nodes and 8 edges. The detected trees, sorted by decreasing size, are:

- Tree starting from the root node ENSP00000303028 (degree 2), and containing 9 nodes, with a maximal depth of 4, which are ENSP00000362386 (degree 3), ENSP00000385592, ENSP00000272438, ENSP00000364643 and ENSP00000384887 (degree 3).
- Tree starting from the root node ENSP00000355045 (degree 2), and containing 5 nodes, with a maximal depth of 2, which are ENSP00000379434, ENSP00000381857, ENSP00000352314 and ENSP00000473941.

Dendritic trees A dendritic tree is a tree-like structure starting from a root node that is part of another strongly connected component. We have detected 14 dendritic trees in the graph, involving a total of 61 nodes (0.31%) and 61 edges (0.01%), with the largest one involving 6 nodes and 6 edges. The detected dendritic trees, sorted by decreasing size, are:

- Dendritic tree starting from the root node ENSP00000254627 (degree 5), and containing 6 nodes, with a maximal depth of 3, which are ENSP00000407107 (degree 5), ENSP00000266022, ENSP00000299308, ENSP00000336693 and ENSP00000391404.
- Dendritic tree starting from the root node ENSP00000366542 (degree 5), and containing 6 nodes, with a maximal depth of 3, which are ENSP00000276127 (degree 3), ENSP00000281722, ENSP00000357889, ENSP00000362206 and ENSP00000306776.
- Dendritic tree starting from the root node ENSP00000310686 (degree 14), and containing 5 nodes, with a maximal depth of 3, which are ENSP00000482345, ENSP00000300896 (degree 4), ENSP00000478426, ENSP00000478683 and ENSP00000483965.
- Dendritic tree starting from the root node ENSP00000311336 (degree 5), and containing 5 nodes, with a maximal depth of 3, which are ENSP00000232892 (degree 3), ENSP00000353557, ENSP00000355156, ENSP00000362814 and ENSP00000348911.
- Dendritic tree starting from the root node ENSP00000446916 (degree 4), and containing 5 nodes, with a maximal depth of 2, which are ENSP00000373583 (degree 5), ENSP00000352299, ENSP00000357811, ENSP00000386118 and ENSP00000387298.
- Dendritic tree starting from the root node ENSP00000265322 (degree 43), and containing 4 nodes, with a maximal depth of 3, which are ENSP00000333183, ENSP00000467301 (degree 3), ENSP00000331435 and ENSP00000370801.

And other 8 dendritic trees.

Stars A star is a tree with a maximal depth of one, where nodes with maximal unique degree one are connected to a central root node with a high degree. We have detected 11 stars in the graph, involving a total of 35 nodes (0.18%) and 24 edges, with the largest one involving 4 nodes and 3 edges. The detected stars, sorted by decreasing size, are:

- Star starting from the root node ENSP00000334289 (degree 3), and containing 4 nodes, with a maximal depth of 1, which are ENSP00000324672, ENSP00000366223 and ENSP00000485629.
- Star starting from the root node ENSP00000345333 (degree 3), and containing 4 nodes, with a maximal depth of 1, which are ENSP00000328245, ENSP00000339314 and ENSP00000483077.
- Star starting from the root node ENSP00000281523 (degree 2), and containing 3 nodes, with a maximal depth of 1, which are ENSP00000297063 and ENSP00000418575.

- Star starting from the root node ENSP00000295012 (degree 2), and containing 3 nodes, with a maximal depth of 1, which are ENSP00000469705 and ENSP00000484537.
- Star starting from the root node ENSP00000319590 (degree 2), and containing 3 nodes, with a maximal depth of 1, which are ENSP00000230301 and ENSP00000354590.
- Star starting from the root node ENSP00000344129 (degree 2), and containing 3 nodes, with a maximal depth of 1, which are ENSP00000380071 and ENSP00000469417.

And other 5 stars.

Dendritic stars A dendritic star is a dendritic tree with a maximal depth of one, where nodes with maximal unique degree one are connected to a central root node with high degree and inside a strongly connected component. We have detected 90 dendritic stars in the graph, involving a total of 220 nodes (1.12%) and 220 edges (0.04%), with the largest one involving 19 nodes and 19 edges. The detected dendritic stars, sorted by decreasing size, are:

- Dendritic star starting from the root node ENSP00000334051 (degree 82), and containing 19 nodes, with a maximal depth of 1, which are ENSP00000284287, ENSP00000307726, ENSP00000311605, ENSP00000318997 and ENSP00000319071.
- Dendritic star starting from the root node ENSP00000432561 (degree 14), and containing 7 nodes, with a maximal depth of 1, which are ENSP00000254166, ENSP00000303533, ENSP00000344162, ENSP00000379464 and ENSP00000385163.
- Dendritic star starting from the root node ENSP00000319673 (degree 16), and containing 6 nodes, with a maximal depth of 1, which are ENSP00000360360, ENSP00000392283, ENSP00000393315, ENSP00000399711 and ENSP00000410400.
- Dendritic star starting from the root node ENSP00000376345 (degree 380), and containing 4 nodes, with a maximal depth of 1, which are ENSP00000320038, ENSP00000356093, ENSP00000384593 and ENSP00000470441.
- Dendritic star starting from the root node ENSP00000267017 (degree 20), and containing 4 nodes, with a maximal depth of 1, which are ENSP00000309782, ENSP00000314042, ENSP00000330612 and ENSP00000366331.
- Dendritic star starting from the root node ENSP00000246801 (degree 7), and containing 3 nodes, with a maximal depth of 1, which are ENSP00000362634, ENSP00000375081 and ENSP00000382544.

And other 84 dendritic stars.

Dendritic tendril stars A dendritic tendril star is a dendritic tree with a depth greater than one, where the arms of the star are tendrils. We have detected 13 dendritic tendril stars in the graph, involving a total of 48 nodes (0.25%) and 48 edges, with the largest one involving 9 nodes and 9 edges. The detected dendritic tendril stars, sorted by decreasing size, are:

- Dendritic tendril star starting from the root node ENSP00000371594 (degree 68), and containing 9 nodes, with a maximal depth of 2, which are ENSP00000302199, ENSP00000312403, ENSP00000323853, ENSP00000329982 and ENSP00000330904.
- Dendritic tendril star starting from the root node ENSP00000310275 (degree 58), and containing 5 nodes, with a maximal depth of 4, which are ENSP00000356121, ENSP00000360320, ENSP00000351727, ENSP00000355840 and ENSP00000340887.
- Dendritic tendril star starting from the root node ENSP00000363382 (degree 4), and containing 4 nodes, with a maximal depth of 3, which are ENSP00000332663, ENSP00000363894, ENSP00000448580 and ENSP00000363899.
- Dendritic tendril star starting from the root node ENSP00000216862 (degree 24), and containing 3 nodes, with a maximal depth of 2, which are ENSP00000309649, ENSP00000318912 and ENSP00000242315.

- Dendritic tendril star starting from the root node ENSP00000276590 (degree 10), and containing 3 nodes, with a maximal depth of 2, which are ENSP00000262288, ENSP00000271375 and ENSP00000348033.
- Dendritic tendril star starting from the root node ENSP00000283946 (degree 10), and containing 3 nodes, with a maximal depth of 2, which are ENSP00000359819, ENSP00000377577 and ENSP00000229002.

And other 7 dendritic tendril stars.

Tendrils A tendril is a path starting from a node of degree one, connected to a strongly connected component. We have detected 995 tendrils in the graph, involving a total of 1.08K nodes (5.52%) and 1.08K edges (0.21%), with the largest one involving 4 nodes and 4 edges. The detected tendrils, sorted by decreasing size, are:

- Tendril starting from the root node ENSP00000374014 (degree 7), and containing 4 nodes, with a maximal depth of 4, which are ENSP00000291481, ENSP00000317000, ENSP00000394178 and ENSP00000296862.
- Tendril starting from the root node ENSP00000371512 (degree 13), and containing 3 nodes, with a maximal depth of 3, which are ENSP00000310182, ENSP00000344331 and ENSP00000386389.
- Tendril starting from the root node ENSP00000264554 (degree 44), and containing 3 nodes, with a maximal depth of 3, which are ENSP00000344465, ENSP00000336661 and ENSP00000430271.
- Tendril starting from the root node ENSP00000302578 (degree 4), and containing 3 nodes, with a maximal depth of 3, which are ENSP00000264735, ENSP00000295092 and ENSP00000370724.
- Tendril starting from the root node ENSP00000302843 (degree 6), and containing 3 nodes, with a maximal depth of 3, which are ENSP00000414920, ENSP00000206020 and ENSP00000317289.
- Tendril starting from the root node ENSP00000307636 (degree 6), and containing 3 nodes, with a maximal depth of 3, which are ENSP00000386563, ENSP00000367792 and ENSP00000411253.

And other 989 tendrils.

3.2.2 MusMusculus

The undirected graph MusMusculus has 22.05K nodes and 233.76K edges. The graph contains 5.57K connected components (of which 5.42K are disconnected nodes), with the largest one containing 16.18K nodes and the smallest one containing a single node. The RAM requirements for the nodes and edges data structures are 3.28MB and 739.96KB respectively.

Degree centrality The minimum node degree is 0, the maximum node degree is 684, the mode degree is 0, the mean degree is 21.21 and the node degree median is 6. The nodes with the highest degree centrality are ENSMUSP00000099909 (degree 684), ENSMUSP00000104298 (degree 649), ENSMUSP00000007130 (degree 585), ENSMUSP00000029175 (degree 538) and ENSMUSP00000001780 (degree 517).

Weights The minimum edge weight is 700, the maximum edge weight is 999 and the total edge weight is 394822140. The RAM requirement for the edge weights data structure is 1.87MB.

Topological Oddities A topological oddity is a set of nodes in the graph that *may be derived* by an error during the generation of the edge list of the graph and, depending on the task, could bias the results of topology-based models. In the following paragraph, we will describe the detected topological oddities.

Singleton nodes A singleton node is a node disconnected from all other nodes. We have detected 5.42K singleton nodes in the graph, involving a total of 5.42K nodes (24.56%). The detected singleton nodes are:

- ENSMUSP00000000208
- ENSMUSP00000000266
- ENSMUSP00000000327
- ENSMUSP00000000449
- ENSMUSP00000000619
- ENSMUSP00000000717

- ENSMUSP00000000755
- ENSMUSP00000001009
- ENSMUSP00000001122
- ENSMUSP00000001172
- ENSMUSP00000001185
- ENSMUSP00000001242
- ENSMUSP00000001456
- ENSMUSP00000001544
- ENSMUSP00000001626

And other 5.40K singleton nodes.

Node tuples A node tuple is a connected component composed of two nodes. We have detected 98 node tuples in the graph, involving a total of 196 nodes (0.89%) and 98 edges (0.02%). The detected node tuples are:

- Node tuple containing the nodes ENSMUSP00000139506 and ENSMUSP00000140944.
- Node tuple containing the nodes ENSMUSP00000139148 and ENSMUSP00000140416.
- Node tuple containing the nodes ENSMUSP00000137187 and ENSMUSP00000137299.
- Node tuple containing the nodes ENSMUSP00000135899 and ENSMUSP00000136712.
- Node tuple containing the nodes ENSMUSP00000126635 and ENSMUSP00000132971.
- Node tuple containing the nodes ENSMUSP00000125936 and ENSMUSP00000136153.
- Node tuple containing the nodes ENSMUSP00000121288 and ENSMUSP00000129762.
- Node tuple containing the nodes ENSMUSP00000113317 and ENSMUSP00000128826.
- Node tuple containing the nodes ENSMUSP00000109660 and ENSMUSP00000111141.
- Node tuple containing the nodes ENSMUSP00000102037 and ENSMUSP00000133699.
- Node tuple containing the nodes ENSMUSP00000101431 and ENSMUSP00000111088.
- Node tuple containing the nodes ENSMUSP00000100068 and ENSMUSP00000107129.
- Node tuple containing the nodes ENSMUSP00000097268 and ENSMUSP00000100772.
- Node tuple containing the nodes ENSMUSP00000097041 and ENSMUSP00000112932.
- Node tuple containing the nodes ENSMUSP00000096773 and ENSMUSP00000114092.

And other 83 node tuples.

Isomorphic node groups Isomorphic groups are nodes with exactly the same neighbours and node types (if present in the graph). Nodes in such groups are topologically indistinguishable, that is swapping their ID would not change the graph topology. We have detected 66 isomorphic node groups in the graph, involving a total of 179 nodes (0.81%) and 6.42K edges (1.37%), with the largest one involving 11 nodes and 590 edges. The detected isomorphic node groups, sorted by decreasing size, are:

- Group with 5 nodes (degree 118): ENSMUSP00000132343, ENSMUSP00000096592, ENSMUSP00000128232, ENSMUSP00000100655 and ENSMUSP00000129788.
- Group with 5 nodes (degree 103): ENSMUSP00000100589, ENSMUSP00000071496, ENSMUSP00000138961, ENSMUSP00000087632 and ENSMUSP00000092345.
- Group with 2 nodes (degree 219): ENSMUSP00000126892 and ENSMUSP00000128443.
- Group with 2 nodes (degree 207): ENSMUSP00000138342 and ENSMUSP00000127859.
- Group with 3 nodes (degree 137): ENSMUSP00000126334, ENSMUSP00000110610 and ENSMUSP00000132241.
- Group with 2 nodes (degree 191): ENSMUSP00000128489 and ENSMUSP00000131471.
- Group with 2 nodes (degree 186): ENSMUSP00000137545 and ENSMUSP00000100516.

- Group with 2 nodes (degree 172): ENSMUSP00000100805 and ENSMUSP00000100528.
- Group with 2 nodes (degree 160): ENSMUSP00000129583 and ENSMUSP00000129695.
- Group with 2 nodes (degree 147): ENSMUSP00000136791 and ENSMUSP00000080543.
- Group with 2 nodes (degree 138): ENSMUSP00000093124 and ENSMUSP00000136275.
- Group with 2 nodes (degree 137): ENSMUSP00000082867 and ENSMUSP00000078747.
- Group with 11 nodes (degree 22): ENSMUSP00000025322, ENSMUSP00000109371, ENSMUSP00000040435, ENSMUSP00000058686, ENSMUSP00000084411 and other 6.
- Group with 5 nodes (degree 40): ENSMUSP00000100769, ENSMUSP00000136763, ENSMUSP00000088719, ENSMUSP00000137043 and ENSMUSP00000126567.
- Group with 2 nodes (degree 82): ENSMUSP00000138211 and ENSMUSP00000138181.

And other 51 isomorphic node groups.

Dendritic trees A dendritic tree is a tree-like structure starting from a root node that is part of another strongly connected component. We have detected 19 dendritic trees in the graph, involving a total of 81 nodes (0.37%) and 81 edges (0.02%), with the largest one involving 9 nodes and 9 edges. The detected dendritic trees, sorted by decreasing size, are:

- Dendritic tree starting from the root node ENSMUSP00000132092 (degree 4), and containing 9 nodes, with a maximal depth of 7, which are ENSMUSP00000092725, ENSMUSP00000094845, ENSMUSP00000049864 (degree 3), ENSMUSP00000037596 and ENSMUSP00000054292.
- Dendritic tree starting from the root node ENSMUSP00000038744 (degree 202), and containing 6 nodes, with a maximal depth of 2, which are ENSMUSP00000038638, ENSMUSP00000055692, ENSMUSP00000064785, ENSMUSP00000106128 and ENSMUSP00000063248.
- Dendritic tree starting from the root node ENSMUSP00000044998 (degree 10), and containing 6 nodes, with a maximal depth of 5, which are ENSMUSP00000053849, ENSMUSP00000039821 (degree 3), ENSMUSP00000036503, ENSMUSP00000050902 and ENSMUSP00000043513.
- Dendritic tree starting from the root node ENSMUSP00000086311 (degree 3), and containing 6 nodes, with a maximal depth of 4, which are ENSMUSP00000085528, ENSMUSP00000088822 (degree 3), ENSMUSP00000028283, ENSMUSP00000085531 and ENSMUSP00000109664.
- Dendritic tree starting from the root node ENSMUSP00000014830 (degree 11), and containing 5 nodes, with a maximal depth of 2, which are ENSMUSP00000057433 (degree 5), ENSMUSP00000032520, ENSMUSP00000032663, ENSMUSP00000047914 and ENSMUSP00000092344.
- Dendritic tree starting from the root node ENSMUSP00000063839 (degree 62), and containing 5 nodes, with a maximal depth of 3, which are ENSMUSP00000029800 (degree 3), ENSMUSP00000035263, ENSMUSP00000062837, ENSMUSP00000096097 and ENSMUSP00000096443.

And other 13 dendritic trees.

Stars A star is a tree with a maximal depth of one, where nodes with maximal unique degree one are connected to a central root node with a high degree. We have detected 26 stars in the graph, involving a total of 82 nodes (0.37%) and 56 edges (0.01%), with the largest one involving 5 nodes and 4 edges. The detected stars, sorted by decreasing size, are:

- Star starting from the root node ENSMUSP00000062719 (degree 4), and containing 5 nodes, with a maximal depth of 1, which are ENSMUSP00000076107, ENSMUSP00000078756, ENSMUSP00000079039 and ENSMUSP00000095815.
- Star starting from the root node ENSMUSP00000110147 (degree 3), and containing 4 nodes, with a maximal depth of 1, which are ENSMUSP00000063474, ENSMUSP0000070718 and ENSMUSP00000132644.

- Star starting from the root node ENSMUSP00000076458 (degree 3), and containing 4 nodes, with a maximal depth of 1, which are ENSMUSP00000033575, ENSMUSP00000076472 and ENSMUSP00000083892.
- Star starting from the root node ENSMUSP00000038678 (degree 2), and containing 3 nodes, with a maximal depth of 1, which are ENSMUSP00000077650 and ENSMUSP00000110164.
- Star starting from the root node ENSMUSP00000113392 (degree 2), and containing 3 nodes, with a maximal depth of 1, which are ENSMUSP00000097485 and ENSMUSP00000107204.
- Star starting from the root node ENSMUSP00000096752 (degree 2), and containing 3 nodes, with a maximal depth of 1, which are ENSMUSP00000135685 and ENSMUSP00000137012.

And other 20 stars.

Dendritic stars A dendritic star is a dendritic tree with a maximal depth of one, where nodes with maximal unique degree one are connected to a central root node with high degree and inside a strongly connected component. We have detected 106 dendritic stars in the graph, involving a total of 352 nodes (1.60%) and 352 edges (0.08%), with the largest one involving 95 nodes and 95 edges. The detected dendritic stars, sorted by decreasing size, are:

- Dendritic star starting from the root node ENSMUSP00000025402 (degree 190), and containing 95 nodes, with a maximal depth of 1, which are ENSMUSP00000038992, ENSMUSP00000041524, ENSMUSP00000050544, ENSMUSP00000050833 and ENSMUSP00000051280.
- Dendritic star starting from the root node ENSMUSP00000033300 (degree 20), and containing 16 nodes, with a maximal depth of 1, which are ENSMUSP00000071783, ENSMUSP00000074745, ENSMUSP00000076041, ENSMUSP00000076665 and ENSMUSP00000079376.
- Dendritic star starting from the root node ENSMUSP00000041483 (degree 43), and containing 6 nodes, with a maximal depth of 1, which are ENSMUSP00000015585, ENSMUSP00000015587, ENSMUSP00000015594, ENSMUSP00000022757 and ENSMUSP00000080742.
- Dendritic star starting from the root node ENSMUSP00000131269 (degree 167), and containing 6 nodes, with a maximal depth of 1, which are ENSMUSP00000052312, ENSMUSP00000071824, ENSMUSP00000077990, ENSMUSP00000079893 and ENSMUSP00000087276.
- Dendritic star starting from the root node ENSMUSP00000006235 (degree 37), and containing 5 nodes, with a maximal depth of 1, which are ENSMUSP00000023619, ENSMUSP00000078181, ENSMUSP00000087054, ENSMUSP00000093794 and ENSMUSP00000093795.
- Dendritic star starting from the root node ENSMUSP00000074436 (degree 33), and containing 4 nodes, with a maximal depth of 1, which are ENSMUSP00000082127, ENSMUSP00000105584, ENSMUSP00000113945 and ENSMUSP00000140024.

And other 100 dendritic stars.

Dendritic tendril stars A dendritic tendril star is a dendritic tree with a depth greater than one, where the arms of the star are tendrils. We have detected 24 dendritic tendril stars in the graph, involving a total of 93 nodes (0.42%) and 93 edges (0.02%), with the largest one involving 15 nodes and 15 edges. The detected dendritic tendril stars, sorted by decreasing size, are:

- Dendritic tendril star starting from the root node ENSMUSP00000122219 (degree 36), and containing 15 nodes, with a maximal depth of 2, which are ENSMUSP00000021776, ENSMUSP00000021778, ENSMUSP00000021779, ENSMUSP00000023602 and ENSMUSP00000046522.
- Dendritic tendril star starting from the root node ENSMUSP00000031011 (degree 134), and containing 7 nodes, with a maximal depth of 2, which are ENSMUSP00000032185, ENSMUSP00000040429, ENSMUSP00000059419, ENSMUSP00000094334 and ENSMUSP00000130758.

- Dendritic tendril star starting from the root node ENSMUSP00000130738 (degree 144), and containing 5 nodes, with a maximal depth of 2, which are ENSMUSP00000002880, ENSMUSP00000086835, ENSMUSP00000094097, ENSMUSP00000114489 and ENSMUSP00000033210.
- Dendritic tendril star starting from the root node ENSMUSP00000049228 (degree 128), and containing 4 nodes, with a maximal depth of 2, which are ENSMUSP00000030878, ENSMUSP00000036916, ENSMUSP00000076935 and ENSMUSP00000033414.
- Dendritic tendril star starting from the root node ENSMUSP00000104298 (degree 649), and containing 4 nodes, with a maximal depth of 2, which are ENSMUSP00000031434, ENSMUSP00000118809, ENSMUSP00000130176 and ENSMUSP00000071896.
- Dendritic tendril star starting from the root node ENSMUSP00000051068 (degree 22), and containing 4 nodes, with a maximal depth of 3, which are ENSMUSP00000044228, ENSMUSP00000110316, ENSMUSP00000034413 and ENSMUSP00000136717.

And other 18 dendritic tendril stars.

Free-floating chains A free-floating chain is a tree with maximal degree two. We have detected 2 free-floating chains in the graph, involving a total of 9 nodes (0.04%) and 7 edges, with the largest one involving 5 nodes and 4 edges. The detected free-floating chains, sorted by decreasing size, are:

- Free-floating chain starting from the root node ENSMUSP00000069019 (degree 3), and containing 5 nodes, with a maximal depth of 2, which are ENSMUSP00000090857, ENSMUSP00000101667, ENSMUSP00000117294 and ENSMUSP00000033804.
- Free-floating chain starting from the root node ENSMUSP00000104573 (degree 2), and containing 4 nodes, with a maximal depth of 2, which are ENSMUSP00000136794, ENSMUSP00000137575 and ENSMUSP00000104609.

Tendrils A tendril is a path starting from a node of degree one, connected to a strongly connected component. We have detected 1.05K tendrils in the graph, involving a total of 1.16K nodes (5.27%) and 1.16K edges (0.25%), with the largest one involving 4 nodes and 4 edges. The detected tendrils, sorted by decreasing size, are:

- Tendril starting from the root node ENSMUSP00000030446 (degree 14), and containing 4 nodes, with a maximal depth of 4, which are ENSMUSP00000098200, ENSMUSP00000087257, ENSMUSP00000020926 and ENSMUSP00000065613.
- Tendril starting from the root node ENSMUSP00000061753 (degree 4), and containing 3 nodes, with a maximal depth of 3, which are ENSMUSP00000101679, ENSMUSP00000120070 and ENSMUSP00000086041.
- Tendril starting from the root node ENSMUSP00000023673 (degree 70), and containing 3 nodes, with a maximal depth of 3, which are ENSMUSP00000088678, ENSMUSP00000078374 and ENSMUSP00000068904.
- Tendril starting from the root node ENSMUSP00000026986 (degree 4), and containing 3 nodes, with a maximal depth of 3, which are ENSMUSP00000001080, ENSMUSP00000058119 and ENSMUSP00000104752.
- Tendril starting from the root node ENSMUSP00000131648 (degree 102), and containing 3 nodes, with a maximal depth of 3, which are ENSMUSP00000058810, ENSMUSP00000090326 and ENSMUSP00000067699.
- Tendril starting from the root node ENSMUSP00000011526 (degree 8), and containing 3 nodes, with a maximal depth of 3, which are ENSMUSP00000029325, ENSMUSP00000029326 and ENSMUSP00000129444.

And other 1.04K tendrils.

3.2.3 Homo Phenotype Ontology

The undirected multigraph HP has 43.47K homogeneous nodes and 88.96K heterogeneous edges. The graph contains 551 connected components (of which 543 are disconnected nodes), with the largest one containing 42.83K nodes and the smallest one containing a single node. The RAM requirements for the nodes and edges data structures are 5.45MB and 359.04KB respectively.

Degree centrality The minimum node degree is 0, the maximum node degree is 1.96K, the mode degree is 1, the mean degree is 4.09 and the node degree median is 2. The nodes with the highest degree centrality are GO:0065007 (degree 1.96K and node type biolink:NamedThing), UBERON_CORE:pheno_slim (degree 1.72K and node type biolink:NamedThing), OBO:chebi#3_STAR (degree 1.52K and node type biolink:NamedThing), UBERON_CORE:uberon_slim (degree 900 and node type biolink:NamedThing) and OBO:hp#hposlim_core (degree 840 and node type biolink:NamedThing).

Node types The graph has a single node type, which is biolink:NamedThing. The RAM requirement for the node types data structure is 2.26MB.

Homogeneous node types Homogeneous node types are node types that are assigned to all the nodes in the graph, making the node type relatively meaningless, as it adds no more information than the fact that the node is in the graph. The graph contains a homogeneous node type, which is biolink:NamedThing.

Edge types The graph has 46 edge types, of which the 10 most common are biolink:subclass_of (95.86K edges, 53.90%), biolink:related_to (24.77K edges, 13.93%), biolink:has_part (14.28K edges, 8.03%), biolink:part_of (13.77K edges, 7.74%), biolink:close_match (11.76K edges, 6.61%), biolink:develops_from (2.59K edges, 1.46%), biolink:has_attribute (1.60K edges, 0.90%), biolink:regulates (1.48K edges, 0.83%), biolink:positively_regulates (1.25K edges, 0.71%) and biolink:negatively_regulates (1.25K edges, 0.70%). The RAM requirement for the edge types data structure is 1.43MB.

Topological Oddities A topological oddity is a set of nodes in the graph that *may be derived* by an error during the generation of the edge list of the graph and, depending on the task, could bias the results of topology-based models. In the following paragraph, we will describe the detected topological oddities.

Singleton nodes A singleton node is a node disconnected from all other nodes. We have detected 539 singleton nodes in the graph, involving a total of 539 nodes (1.24%). The detected singleton nodes are:

- is_substituent_group_from (node type biolink:NamedThing)
- BFO:0000056 (node type biolink:NamedThing)
- OBO:cl#lacks_part (node type biolink:NamedThing)
- OBO:pr#has_gene_template (node type biolink:NamedThing)
- GOREL:0002003 (node type biolink:NamedThing)
- RO:0015011 (node type biolink:NamedThing)
- UBERON_CORE:channels_from (node type biolink:NamedThing)
- UBERON_CORE:synapsed_by (node type biolink:NamedThing)
- OBO:nbo#has_participant (node type biolink:NamedThing)
- UBERON_CORE:filtered_through (node type biolink:NamedThing)
- UBERON_CORE:site_of (node type biolink:NamedThing)
- OBO:chebi#is_tautomer_of (node type biolink:NamedThing)
- RO:0015012 (node type biolink:NamedThing)
- UBERON_CORE:indirectly_supplies (node type biolink:NamedThing)
- OBO:nbo#has-input (node type biolink:NamedThing)

And other 524 singleton nodes.

Singleton nodes with self-loops A singleton node with self-loops is a node disconnected from all other nodes except itself. We have detected 4 singleton nodes with self-loops in the graph, involving a total of 4 nodes and 4 edges. The detected singleton nodes with self-loops are:

- dc:description (node type biolink:NamedThing)
- dcterms-license (node type biolink:NamedThing)
- dc:title (node type biolink:NamedThing)
- dc:contributor (node type biolink:NamedThing)

Node tuples A node tuple is a connected component composed of two nodes. We have detected 2 node tuples in the graph, involving a total of 4 nodes and 2 edges. The detected node tuples are:

- Node tuple containing the nodes BSPO:0000098 (node type biolink:NamedThing) and ventral_to (node type biolink:NamedThing).
- Node tuple containing the nodes OBO:chebi#is_conjugate_acid_of (node type biolink:NamedThing) and OBO:chebi#is_conjugate_base_of (node type biolink:NamedThing).

Isomorphic node groups Isomorphic groups are nodes with exactly the same neighbours and node types (if present in the graph). Nodes in such groups are topologically indistinguishable, that is swapping their ID would not change the graph topology. We have detected 4 isomorphic node groups in the graph, involving a total of 9 nodes (0.02%) and 50 edges (0.03%), with the largest one involving 3 nodes and 18 edges. The detected isomorphic node groups, sorted by decreasing size, are:

- Group with 3 nodes (degree 6 and node type biolink:NamedThing): RO:0002296, RO:0002298 and RO:0002299.
- Group with 2 nodes (degree 6 and node type biolink:NamedThing): UBERON:0013773 and UBERON:0013772.
- Group with 2 nodes (degree 5 and node type biolink:NamedThing): RO:0002232 and RO:0002231.
- Group with 2 nodes (degree 5 and node type biolink:NamedThing): NCBITaxon:3176 and NCBITaxon:3378.

Dendritic trees A dendritic tree is a tree-like structure starting from a root node that is part of another strongly connected component. We have detected 721 dendritic trees in the graph, involving a total of 9.92K nodes (22.81%) and 9.92K edges (5.58%), with the largest one involving 249 nodes and 249 edges. The detected dendritic trees, sorted by decreasing size, are:

- Dendritic tree starting from the root node HP:0002960 (degree 9), and containing 249 nodes, with a maximal depth of 5, which are HP:0030057 (degree 164), HP:0002725, Nbf08f17380a543868ede822e13da03e7, HP:0032265 and HP:5000021 (degree 3). Its nodes have a single node type, which is biolink:NamedThing. Its edges have 2 edge types, which are biolink:subclass_of (493 edges, 99.80%) and biolink:has_part.
- Dendritic tree starting from the root node HP:0033354 (degree 45), and containing 140 nodes, with a maximal depth of 4, which are HP:0011279 (degree 4), HP:0033187, HP:0012404 (degree 4), HP:0003541 (degree 3) and HP:0011814. Its nodes have a single node type, which is biolink:NamedThing. Its edges have 2 edge types, which are biolink:subclass_of (157 edges, 64.08%) and biolink:has_part (88 edges, 35.92%).
- Dendritic tree starting from the root node HP:0010876 (degree 55), and containing 111 nodes, with a maximal depth of 5, which are HP:0031222, HP:0032463, HP:0012509, HP:0002152 and HP:0025201 (degree 3). Its nodes have a single node type, which is biolink:NamedThing. Its edges have 2 edge types, which are biolink:subclass_of (149 edges, 84.66%) and biolink:has_part (27 edges, 15.34%).
- Dendritic tree starting from the root node HP:0004303 (degree 28), and containing 103 nodes, with a maximal depth of 3, which are HP:0030089 (degree 19), HP:0012269 (degree 4), HP:0003791, HP:0030230 and HP:0100298. Its nodes have a single node type, which is biolink:NamedThing. Its edges have 2 edge types, which are biolink:subclass_of (157 edges, 87.22%) and biolink:has_part (23 edges, 12.78%).

- Dendritic tree starting from the root node HP:0000708 (degree 46), and containing 98 nodes, with a maximal depth of 4, which are HP:4000009, HP:0033051 (degree 6), HP:0033676 (degree 3), HP:0030858 (degree 3) and HP:0000711 (degree 3). Its nodes have a single node type, which is biolink:NamedThing. Its edges have 2 edge types, which are biolink:subclass_of (142 edges, 85.03%) and biolink:has_part (25 edges, 14.97%).
- Dendritic tree starting from the root node HP:0012379 (degree 52), and containing 96 nodes, with a maximal depth of 4, which are HP:0000816 (degree 5), HP:0033168, HP:0032459 (degree 3), HP:0031821 (degree 3) and HP:0034202 (degree 3). Its nodes have a single node type, which is biolink:NamedThing. Its edges have 2 edge types, which are biolink:subclass_of (92 edges, 62.16%) and biolink:has_part (56 edges, 37.84%).

And other 715 dendritic trees.

Stars A star is a tree with a maximal depth of one, where nodes with maximal unique degree one are connected to a central root node with a high degree. We have detected 2 stars in the graph, involving a total of 9 nodes (0.02%) and 7 edges, with the largest one involving 6 nodes and 5 edges. The detected stars, sorted by decreasing size, are:

- Star starting from the root node OIO:SynonymTypeProperty (degree 5), and containing 6 nodes, with a maximal depth of 1, which are OBO:hp#layperson, OBO:hp#uk_spelling, OBO:hp#obsolete_synonym, OBO:hp#abbreviation and OBO:hp#plural_form. Its nodes have a single node type, which is biolink:NamedThing. Its edges have a single edge type, which is biolink:subclass_of.
- Star starting from the root node CHEBI:21241 (degree 2), and containing 3 nodes, with a maximal depth of 1, which are IAO:0000227 and CHEBI:176783. Its nodes have a single node type, which is biolink:NamedThing. Its edges have a single edge type, which is biolink:related_to.

Dendritic stars A dendritic star is a dendritic tree with a maximal depth of one, where nodes with maximal unique degree one are connected to a central root node with high degree and inside a strongly connected component. We have detected 2.47K dendritic stars in the graph, involving a total of 6.87K nodes (15.81%) and 6.87K edges (3.86%), with the largest one involving 16 nodes and 16 edges. The detected dendritic stars, sorted by decreasing size, are:

- Dendritic star starting from the root node MAXO:0000072 (degree 28), and containing 16 nodes, with a maximal depth of 1, which are MAXO:0010202, MAXO:0010211, MAXO:0010222, MAXO:0010218 and MAXO:0010219. Its nodes have a single node type, which is biolink:NamedThing. Its edges have a single edge type, which is biolink:subclass_of.
- Dendritic star starting from the root node BFO:0000050 (degree 40), and containing 13 nodes, with a maximal depth of 1, which are BSPO:0015102, BSPO:0001115, BSPO:0005001, BSPO:0000122 and BSPO:0015101. Its nodes have a single node type, which is biolink:NamedThing. Its edges have 3 edge types, which are biolink:subclass_of (10 edges, 76.92%), rdfs:seeAlso (2 edges, 15.38%) and biolink:related_to.
- Dendritic star starting from the root node UBERON:0002149 (degree 24), and containing 12 nodes, with a maximal depth of 1, which are http://braininfo.rprc.washington.edu/centraldirectory.aspx?ID=590, http://www.snomedbrowser.com/Codes/Details/369028007, UBERON:0003011, UBERON:0002143 and UBERON:0002963. Its nodes have a single node type, which is biolink:NamedThing. Its edges have 2 edge types, which are biolink:related_to (8 edges, 66.67%) and biolink:close_match (4 edges, 33.33%).
- Dendritic star starting from the root node HP:0003540 (degree 13), and containing 11 nodes, with a maximal depth of 1, which are HP:0031128, HP:0011894, N709ece82e4b04f06a9daa5b99744af65, HP:0008320 and HP:0031129. Its nodes have a single node type, which is biolink:NamedThing. Its edges have 2 edge types, which are biolink:subclass_of (10 edges, 90.91%) and biolink:has_part.

- Dendritic star starting from the root node HP:0000972 (degree 14), and containing 11 nodes, with a maximal depth of 1, which are HP:0007548, HP:0007497, HP:0000982, HP:0007553 and HP:0007613. Its nodes have a single node type, which is biolink:NamedThing. Its edges have a single edge type, which is biolink:subclass_of.
- Dendritic star starting from the root node UBERON:0002871 (degree 21), and containing 11 nodes, with a maximal depth of 1, which are UBERON:0002155, UBERON:0002559, UBERON:0002127, http://www.snomedbrowser.com/Codes/Details/47361005 and UBERON:0002154. Its nodes have a single node type, which is biolink:NamedThing. Its edges have 2 edge types, which are biolink:related_to (8 edges, 72.73%) and biolink:close_match (3 edges, 27.27%).

And other 2.47K dendritic stars.

Dendritic tendril stars A dendritic tendril star is a dendritic tree with a depth greater than one, where the arms of the star are tendrils. We have detected 391 dendritic tendril stars in the graph, involving a total of 1.60K nodes (3.69%) and 1.60K edges (0.90%), with the largest one involving 17 nodes and 17 edges. The detected dendritic tendril stars, sorted by decreasing size, are:

- Dendritic tendril star starting from the root node HP:0001878 (degree 18), and containing 17 nodes, with a maximal depth of 2, which are HP:0004863, HP:0005511, HP:0004802, HP:0001930 and HP:0005524. Its nodes have a single node type, which is biolink:NamedThing. Its edges have 2 edge types, which are biolink:subclass_of (15 edges, 83.33%) and biolink:has_part (3 edges, 16.67%).
- Dendritic tendril star starting from the root node MAXO:0010365 (degree 14), and containing 13 nodes, with a maximal depth of 2, which are MAXO:0010359, MAXO:0010361, MAXO:0010351, MAXO:0010355 and MAXO:0010357. Its nodes have a single node type, which is biolink:NamedThing. Its edges have a single edge type, which is biolink:subclass_of.
- Dendritic tendril star starting from the root node HP:0031331 (degree 12), and containing 11 nodes, with a maximal depth of 2, which are HP:0033997, HP:0031319, HP:0031332, HP:0031333 and HP:0031339. Its nodes have a single node type, which is biolink:NamedThing. Its edges have a single edge type, which is biolink:subclass_of.
- Dendritic tendril star starting from the root node HP:0000962 (degree 23), and containing 11 nodes, with a maximal depth of 2, which are HP:0005595, HP:0007501, HP:0025080, HP:0007490 and HP:0031288. Its nodes have a single node type, which is biolink:NamedThing. Its edges have 2 edge types, which are biolink:subclass_of (9 edges, 75.00%) and biolink:has_part (3 edges, 25.00%).
- Dendritic tendril star starting from the root node HP:0001832 (degree 27), and containing 10 nodes, with a maximal depth of 2, which are HP:0005194, HP:0008125, HP:0008078, HP:0004699 and HP:0010508. Its nodes have a single node type, which is biolink:NamedThing. Its edges have 2 edge types, which are biolink:subclass_of (8 edges, 72.73%) and biolink:has_part (3 edges, 27.27%).
- Dendritic tendril star starting from the root node HP:0001369 (degree 12), and containing 9 nodes, with a maximal depth of 2, which are HP:0040310, HP:0001370, N49475b12e70c4737aa3ca006af35921e, HP:0040311 and HP:0033037. Its nodes have a single node type, which is biolink:NamedThing. Its edges have 2 edge types, which are biolink:subclass_of (9 edges) and biolink:has_part.

And other 385 dendritic tendril stars.

Free-floating chains A free-floating chain is a tree with maximal degree two. We have detected a single free-floating chain in the graph, involving a total of 5 nodes (0.01%) and 4 edges.

- Free-floating chain starting from the root node RO:0002563 (degree 3), and containing 5 nodes, with a maximal depth of 2, which are RO:0002564, RO:0002464, http://ontologydesignpatterns.org/wiki&#x[illegible] Ary_Relation_Pattern_%28OWL_2%29 and RO:0002481. Its nodes have a single node type, which is biolink:NamedThing. Its edges have 2 edge types, which are biolink:subclass_of (4 edges) and rdfs:seeAlso.

Tendrils A tendril is a path starting from a node of degree one, connected to a strongly connected component. We have detected 4.32K tendrils in the graph, involving a total of 4.35K nodes (10.00%) and 4.35K edges (2.44%), with the largest one involving 3 nodes and 3 edges. The detected tendrils, sorted by decreasing size, are:

- Tendril starting from the root node NBO:0000607 (degree 3), and containing 3 nodes, with a maximal depth of 3, which are NBO:0000006, NBO:0000170 and NBO:0000304. Its nodes have a single node type, which is biolink:NamedThing. Its edges have a single edge type, which is biolink:subclass_of.
- Tendril starting from the root node NBO:0000389 (degree 3), and containing 3 nodes, with a maximal depth of 3, which are NBO:0000411, NBO:0000416 and NBO:0000417. Its nodes have a single node type, which is biolink:NamedThing. Its edges have a single edge type, which is biolink:subclass_of.
- Tendril starting from the root node HP:0100836 (degree 6), and containing 3 nodes, with a maximal depth of 3, which are HP:0002885, HP:0007129 and N520ca489d82d46018dce6dd76acb53a4. Its nodes have a single node type, which is biolink:NamedThing. Its edges have 2 edge types, which are biolink:subclass_of (3 edges) and biolink:has_part (2 edges).
- Tendril starting from the root node HP:0010786 (degree 5), and containing 3 nodes, with a maximal depth of 3, which are HP:0009725, HP:0002862 and HP:0006740. Its nodes have a single node type, which is biolink:NamedThing. Its edges have a single edge type, which is biolink:subclass_of.
- Tendril starting from the root node HP:0006304 (degree 3), and containing 2 nodes, with a maximal depth of 2, which are HP:0001566 and N11df713e04764d4ba280ea7097fd2c5e. Its nodes have a single node type, which is biolink:NamedThing. Its edges have 2 edge types, which are biolink:has_part (2 edges) and biolink:subclass_of.
- Tendril starting from the root node HP:0500148 (degree 5), and containing 2 nodes, with a maximal depth of 2, which are HP:0410068 and Na4825961d95b44a9ae1d3cf34e55f4c7. Its nodes have a single node type, which is biolink:NamedThing. Its edges have 2 edge types, which are biolink:has_part (2 edges) and biolink:subclass_of.

And other 4.31K tendrils.

3.3 Graphs used for node-label prediction experiments

For the node-label prediction experiments Citeseer [25, 27], Cora [25, 27], and Pubmed [28] graphs have been used. To describe them in the following we copied the graph reports automatically computed by *Ensmallen*.

3.3.1 Cora

The undirected graph Cora has 2.71K heterogeneous nodes and 5.28K edges. The graph contains 78 connected components, with the largest one containing 2.48K nodes and the smallest one containing 2 nodes. The RAM requirements for the nodes and edges data structures are 207.68KB and 16.34KB respectively.

Degree centrality The minimum node degree is 1, the maximum node degree is 168, the mode degree is 2, the mean degree is 3.90 and the node degree median is 3. The nodes with the highest degree centrality are 35 (degree 168 and node type Genetic_Algorithms), 6213 (degree 78 and node type Reinforcement_Learning), 1365 (degree 74 and node type Neural_Networks), 3229 (degree 65 and node type Neural_Networks) and 910 (degree 44 and node type Neural_Networks).

Node types The graph has 7 node types, which are Neural_Networks (818 nodes, 30.21%), Probabilistic_Methods (426 nodes, 15.73%), Genetic_Algorithms (418 nodes, 15.44%), Theory (351 nodes, 12.96%), Case_Based (298 nodes, 11.00%), Reinforcement_Learning (217 nodes, 8.01%) and Rule_Learning (180 nodes, 6.65%). The RAM requirement for the node types data structure is 141.72KB.

Topological Oddities A topological oddity is a set of nodes in the graph that *may be derived* by an error during the generation of the edge list of the graph and, depending on the task, could bias the results of topology-based models. In the following paragraph, we will describe the detected topological oddities.

Node tuples A node tuple is a connected component composed of two nodes. We have detected 57 node tuples in the graph, involving a total of 114 nodes (4.21%) and 57 edges (0.54%). The detected node tuples are:

- Node tuple containing the nodes 1105622 (node type Neural_Networks) and 430574 (node type Neural_Networks).
- Node tuple containing the nodes 116512 (node type Neural_Networks) and 1107808 (node type Neural_Networks).
- Node tuple containing the nodes 1107728 (node type Neural_Networks) and 115188 (node type Neural_Networks).
- Node tuple containing the nodes 1136040 (node type Neural_Networks) and 754594 (node type Neural_Networks).
- Node tuple containing the nodes 73972 (node type Case_Based) and 50980 (node type Case_Based).
- Node tuple containing the nodes 628458 (node type Neural_Networks) and 628459 (node type Neural_Networks).
- Node tuple containing the nodes 180301 (node type Probabilistic_Methods) and 1110628 (node type Probabilistic_Methods).
- Node tuple containing the nodes 1133008 (node type Neural_Networks) and 688824 (node type Neural_Networks).
- Node tuple containing the nodes 654519 (node type Genetic_Algorithms) and 1131754 (node type Genetic_Algorithms).
- Node tuple containing the nodes 49720 (node type Probabilistic_Methods) and 49753 (node type Probabilistic_Methods).
- Node tuple containing the nodes 133628 (node type Theory) and 1108570 (node type Theory).
- Node tuple containing the nodes 617378 (node type Neural_Networks) and 1130069 (node type Neural_Networks).
- Node tuple containing the nodes 529165 (node type Neural_Networks) and 1126315 (node type Neural_Networks).
- Node tuple containing the nodes 824245 (node type Neural_Networks) and 1139009 (node type Neural_Networks).
- Node tuple containing the nodes 820661 (node type Neural_Networks) and 817774 (node type Neural_Networks).

And other 42 node tuples.

Isomorphic node groups Isomorphic groups are nodes with exactly the same neighbours and node types (if present in the graph). Nodes in such groups are topologically indistinguishable, that is swapping their ID would not change the graph topology. We have detected 3 isomorphic node groups in the graph, involving a total of 6 nodes (0.22%) and 30 edges (0.28%). The detected isomorphic node groups, sorted by decreasing size, are:

- Group with 2 nodes (degree 5 and node type Genetic_Algorithms): 1104999 and 63832.
- Group with 2 nodes (degree 5 and node type Neural_Networks): 43698 and 31336.

- Group with 2 nodes (degree 5 and node type Neural_Networks): 1154123 and 1154124.

Dendritic trees A dendritic tree is a tree-like structure starting from a root node that is part of another strongly connected component. We have detected 13 dendritic trees in the graph, involving a total of 64 nodes (2.36%) and 64 edges (0.61%), with the largest one involving 9 nodes and 9 edges. The detected dendritic trees, sorted by decreasing size, are:

- Dendritic tree starting from the root node 16819 (degree 14), and containing 9 nodes, with a maximal depth of 4, which are 1131274, 643003, 644843, 1131189 and 645016 (degree 5). Its nodes have a single node type, which is Probabilistic_Methods.
- Dendritic tree starting from the root node 35 (degree 168), and containing 7 nodes, with a maximal depth of 2, which are 1152508, 1137466, 1128945, 1119505 and 15670. Its nodes have a single node type, which is Genetic_Algorithms.
- Dendritic tree starting from the root node 16437 (degree 6), and containing 6 nodes, with a maximal depth of 3, which are 51831, 430329, 127940 (degree 4), 416964 and 1114364. Its nodes have a single node type, which is Neural_Networks.
- Dendritic tree starting from the root node 424540 (degree 3), and containing 5 nodes, with a maximal depth of 3, which are 18536 (degree 3), 1106854, 86923 (degree 3), 18532 and 1114184. Its nodes have a single node type, which is Neural_Networks.
- Dendritic tree starting from the root node 910 (degree 44 and node type Neural_Networks), and containing 5 nodes, with a maximal depth of 2, which are 94953 (node type Neural_Networks), 1122460 (node type Neural_Networks), 1114118 (node type Neural_Networks), 245288 (node type Reinforcement_Learning) and 119712 (node type Genetic_Algorithms). Its nodes have 3 node types, which are Neural_Networks (3 nodes, 0.11%), Reinforcement_Learning and Genetic_Algorithms.
- Dendritic tree starting from the root node 13885 (degree 7 and node type Neural_Networks), and containing 5 nodes, with a maximal depth of 3, which are 84459 (node type Theory), 6238 (degree 4 and node type Theory), 1123991 (node type Probabilistic_Methods), 10793 (node type Theory) and 1130356 (node type Theory). Its nodes have 2 node types, which are Theory (4 nodes, 0.15%) and Probabilistic_Methods.

And other 7 dendritic trees.

Stars A star is a tree with a maximal depth of one, where nodes with maximal unique degree one are connected to a central root node with a high degree. We have detected 3 stars in the graph, involving a total of 9 nodes (0.33%) and 6 edges (0.06%). The detected stars are:

- Star starting from the root node 1112071 (degree 2), and containing 3 nodes, with a maximal depth of 1, which are 212107 and 212097. Its nodes have a single node type, which is Probabilistic_Methods.
- Star starting from the root node 1123215 (degree 2), and containing 3 nodes, with a maximal depth of 1, which are 288107 and 149139. Its nodes have a single node type, which is Theory.
- Star starting from the root node 9559 (degree 2), and containing 3 nodes, with a maximal depth of 1, which are 1102794 and 252725. Its nodes have a single node type, which is Rule_Learning.

Dendritic stars A dendritic star is a dendritic tree with a maximal depth of one, where nodes with maximal unique degree one are connected to a central root node with high degree and inside a strongly connected component. We have detected 29 dendritic stars in the graph, involving a total of 84 nodes (3.10%) and 84 edges (0.80%), with the largest one involving 12 nodes and 12 edges. The detected dendritic stars, sorted by decreasing size, are:

- Dendritic star starting from the root node 1365 (degree 74 and node type Neural_Networks), and containing 12 nodes, with a maximal depth of 1, which are 1105062 (node type Reinforcement_Learning),

853150 (node type Neural_Networks), 949318 (node type Neural_Networks), 1136442 (node type Neural_Networks) and 1132922 (node type Neural_Networks). Its nodes have 2 node types, which are Neural_Networks (11 nodes, 0.41%) and Reinforcement_Learning.

- Dendritic star starting from the root node 20193 (degree 23), and containing 11 nodes, with a maximal depth of 1, which are 1153877, 1153879, 1153889, 1130653 and 1130657. Its nodes have a single node type, which is Case_Based.
- Dendritic star starting from the root node 6913 (degree 12), and containing 4 nodes, with a maximal depth of 1, which are 1105011, 1131230, 703953 and 646289. Its nodes have a single node type, which is Probabilistic_Methods.
- Dendritic star starting from the root node 205196 (degree 9), and containing 4 nodes, with a maximal depth of 1, which are 628766, 1130568, 1130586 and 815073. Its nodes have a single node type, which is Neural_Networks.
- Dendritic star starting from the root node 89547 (degree 13 and node type Theory), and containing 3 nodes, with a maximal depth of 1, which are 1152379 (node type Theory), 1116328 (node type Neural_Networks) and 237376 (node type Theory). Its nodes have 2 node types, which are Theory (2 nodes, 0.07%) and Neural_Networks.
- Dendritic star starting from the root node 31353 (degree 19), and containing 3 nodes, with a maximal depth of 1, which are 286562, 1063773 and 686559. Its nodes have a single node type, which is Neural_Networks.

And other 23 dendritic stars.

Dendritic tendril stars A dendritic tendril star is a dendritic tree with a depth greater than one, where the arms of the star are tendrils. We have detected 7 dendritic tendril stars in the graph, involving a total of 28 nodes (1.03%) and 28 edges (0.27%), with the largest one involving 6 nodes and 6 edges. The detected dendritic tendril stars, sorted by decreasing size, are:

- Dendritic tendril star starting from the root node 3229 (degree 65 and node type Neural_Networks), and containing 6 nodes, with a maximal depth of 3, which are 919885 (node type Neural_Networks), 1125082 (node type Genetic_Algorithms), 7022 (node type Neural_Networks), 1112767 (node type Neural_Networks) and 226698 (node type Neural_Networks). Its nodes have 2 node types, which are Neural_Networks (5 nodes, 0.18%) and Genetic_Algorithms.
- Dendritic tendril star starting from the root node 643069 (degree 4 and node type Probabilistic_Methods), and containing 6 nodes, with a maximal depth of 5, which are 14090 (node type Probabilistic_Methods), 1131192 (node type Probabilistic_Methods), 1103016 (node type Neural_Networks), 14083 (node type Neural_Networks) and 62676 (node type Neural_Networks). Its nodes have 2 node types, which are Neural_Networks (4 nodes, 0.15%) and Probabilistic_Methods (2 nodes, 0.07%).
- Dendritic tendril star starting from the root node 3243 (degree 12 and node type Theory), and containing 4 nodes, with a maximal depth of 3, which are 1103610 (node type Theory), 854434 (node type Neural_Networks), 8961 (node type Reinforcement_Learning) and 1133390 (node type Theory). Its nodes have 3 node types, which are Theory (2 nodes, 0.07%), Neural_Networks and Reinforcement_Learning.
- Dendritic tendril star starting from the root node 5086 (degree 12), and containing 3 nodes, with a maximal depth of 2, which are 354004, 1105698 and 1118546. Its nodes have a single node type, which is Probabilistic_Methods.
- Dendritic tendril star starting from the root node 35863 (degree 5 and node type Reinforcement_Learning), and containing 3 nodes, with a maximal depth of 2, which are 28359 (node type Reinforcement_Learning), 134060 (node type Theory) and 481073 (node type Reinforcement_Learning). Its nodes have 2 node types, which are Reinforcement_Learning (2 nodes, 0.07%) and Theory.

- Dendritic tendril star starting from the root node 162080 (degree 5), and containing 3 nodes, with a maximal depth of 2, which are 738941, 1135345 and 1135455. Its nodes have a single node type, which is Neural_Networks.

And another dendritic tendril star.

Free-floating chains A free-floating chain is a tree with maximal degree two. We have detected 2 free-floating chains in the graph, involving a total of 8 nodes (0.30%) and 6 edges (0.06%). The detected free-floating chains are:

- Free-floating chain starting from the root node 375825 (degree 2), and containing 4 nodes, with a maximal depth of 2, which are 1119623, 421481 and 111770. Its nodes have a single node type, which is Probabilistic_Methods.
- Free-floating chain starting from the root node 430711 (degree 2), and containing 4 nodes, with a maximal depth of 2, which are 671052, 1132416 and 1132406. Its nodes have a single node type, which is Neural_Networks.

Tendrils A tendril is a path starting from a node of degree one, connected to a strongly connected component. We have detected 224 tendrils in the graph, involving a total of 265 nodes (9.79%) and 265 edges (2.51%), with the largest one involving 4 nodes and 4 edges. The detected tendrils, sorted by decreasing size, are:

- Tendril starting from the root node 83847 (degree 4), and containing 4 nodes, with a maximal depth of 4, which are 1130678, 630890, 233106 and 12275. Its nodes have a single node type, which is Neural_Networks.
- Tendril starting from the root node 1140543 (degree 5), and containing 3 nodes, with a maximal depth of 3, which are 120817, 1109873 and 163235. Its nodes have a single node type, which is Neural_Networks.
- Tendril starting from the root node 683404 (degree 4), and containing 3 nodes, with a maximal depth of 3, which are 683360, 522338 and 1132864. Its nodes have a single node type, which is Probabilistic_Methods.
- Tendril starting from the root node 20534 (degree 10 and node type Reinforcement_Learning), and containing 3 nodes, with a maximal depth of 3, which are 13972 (node type Reinforcement_Learning), 1126050 (node type Reinforcement_Learning) and 93318 (node type Neural_Networks). Its nodes have 2 node types, which are Reinforcement_Learning (2 nodes, 0.07%) and Neural_Networks.
- Tendril starting from the root node 66751 (degree 5), and containing 3 nodes, with a maximal depth of 3, which are 1138043, 77108 and 77112. Its nodes have a single node type, which is Theory.
- Tendril starting from the root node 3231 (degree 36 and node type Theory), and containing 2 nodes, with a maximal depth of 2, which are 1113926 (node type Neural_Networks) and 250566 (node type Case_Based). Its nodes have 2 node types, which are Neural_Networks and Case_Based.

And other 218 tendrils.

3.3.2 CiteSeer

The undirected graph CiteSeer has 3.31K heterogeneous nodes and 4.66K edges. The graph contains 438 connected components (of which 48 are disconnected nodes), with the largest one containing 2.11K nodes and the smallest one containing a single node. The RAM requirements for the nodes and edges data structures are 335.92KB and 14.76KB respectively.

Degree centrality The minimum node degree is 1, the maximum node degree is 99, the mode degree is 1, the mean degree is 2.78 and the node degree median is 2. The nodes with the highest degree centrality are brin98anatomy (degree 99 and node type IR), rao95bdi (degree 51 and node type Agents),

chakrabarti98automatic (degree 35 and node type IR), bharat98improved (degree 34 and node type IR) and lawrence98searching (degree 30 and node type IR).

Node types The graph has 6 node types, which are DB (701 nodes, 21.17%), IR (668 nodes, 20.17%), Agents (596 nodes, 18.00%), ML (590 nodes, 17.81%), HCI (508 nodes, 15.34%) and AI (249 nodes, 7.52%). The RAM requirement for the node types data structure is 172.75KB.

Topological Oddities A topological oddity is a set of nodes in the graph that *may be derived* by an error during the generation of the edge list of the graph and, depending on the task, could bias the results of topology-based models. In the following paragraph, we will describe the detected topological oddities.

Singleton nodes with self-loops A singleton node with self-loops is a node disconnected from all other nodes except itself. We have detected 48 singleton nodes with self-loops in the graph, involving a total of 48 nodes (1.45%) and 48 edges (0.52%). The detected singleton nodes with self-loops are:

- 408356 (node type DB)
- eiter98firstorder (node type AI)
- 156949 (node type DB)
- bruno01stholes (node type DB)
- kumar01behaviorbased (node type ML)
- lin01efficiently (node type DB)
- park01segmentbased (node type DB)
- tabuada01feasible (node type Agents)
- 146066 (node type DB)
- 190915 (node type DB)
- 202521 (node type DB)
- 274436 (node type HCI)
- 346149 (node type ML)
- 423028 (node type HCI)
- 43511 (node type DB)

And other 33 singleton nodes with self-loops.

Node tuples A node tuple is a connected component composed of two nodes. We have detected 252 node tuples in the graph, involving a total of 504 nodes (15.22%) and 252 edges (2.74%). The detected node tuples are:

- Node tuple containing the nodes zhang99situated (node type ML) and zhang99towards (node type IR).
- Node tuple containing the nodes zhang01evolutionary (node type DB) and zhang99evolving (node type ML).
- Node tuple containing the nodes wolski00design (node type DB) and wolski98fuzzy (node type ML).
- Node tuple containing the nodes wills00open (node type ML) and wills01open (node type ML).
- Node tuple containing the nodes vilalta00quantification (node type ML) and vilalta01rule (node type ML).
- Node tuple containing the nodes vazov01system (node type IR) and wonsever01contextual (node type IR).
- Node tuple containing the nodes vasconcelos00bayesian (node type ML) and vasconcelos99probabilistic (node type ML).
- Node tuple containing the nodes valencia98algebraic (node type AI) and valencia98hitch (node type AI).
- Node tuple containing the nodes tzouramanis99overlapping (node type DB) and tzouramanis99processing (node type DB).

- Node tuple containing the nodes tourapis01advanced (node type ML) and tourapis01temporal (node type ML).
- Node tuple containing the nodes sterritt00exploring (node type AI) and sterritt01soft (degree 2 and node type ML).
- Node tuple containing the nodes rosenthal00view (node type DB) and rosenthal01administering (node type DB).
- Node tuple containing the nodes paulson00inductive (node type ML) and steel02finding (node type ML).
- Node tuple containing the nodes oria99defining (node type DB) and oria99visualmoql (node type DB).
- Node tuple containing the nodes oard01clef (node type HCI) and oard01evaluating (node type HCI).

And other 237 node tuples.

Isomorphic node groups Isomorphic groups are nodes with exactly the same neighbours and node types (if present in the graph). Nodes in such groups are topologically indistinguishable, that is swapping their ID would not change the graph topology. We have detected 11 isomorphic node groups in the graph, involving a total of 23 nodes (0.69%) and 152 edges (1.65%), with the largest one involving 3 nodes and 24 edges. The detected isomorphic node groups, sorted by decreasing size, are:

- Group with 2 nodes (degree 12 and node type IR): 65816 and baker98distributional.
- Group with 3 nodes (degree 6 and node type IR): 540380, 532128 and 536016.
- Group with 2 nodes (degree 8 and node type HCI): 150449 and billinghurst98wearable.
- Group with 2 nodes (degree 7 and node type DB): 254693 and 352789.
- Group with 2 nodes (degree 6 and node type IR): 323867 and lawrence99searching.
- Group with 2 nodes (degree 6 and node type IR): 509763 and nguyen00active.
- Group with 2 nodes (degree 6 and node type Agents): 295535 and kumar00adaptive.
- Group with 2 nodes (degree 6 and node type DB): artale01reasoning and 454077.
- Group with 2 nodes (degree 6 and node type DB): jagadish99querying and 106339.
- Group with 2 nodes (degree 5 and node type DB): 486074 and 506324.
- Group with 2 nodes (degree 5 and node type AI): 28223 and 40513.

Trees A tree is a connected component with n nodes and n-1 edges. We have detected 7 trees in the graph, involving a total of 46 nodes (1.39%) and 39 edges (0.42%), with the largest one involving 9 nodes and 8 edges. The detected trees, sorted by decreasing size, are:

- Tree starting from the root node edmond98achieving (degree 2 and node type DB), and containing 9 nodes, with a maximal depth of 3, which are barros97towards (node type HCI), manolescu01microworkflow (node type IR), barros96business (degree 4 and node type HCI), muth99integrating (node type DB) and 70863 (node type HCI). Its nodes have 3 node types, which are HCI (5 nodes, 0.15%), DB (2 nodes, 0.06%) and IR.
- Tree starting from the root node bonnet99query (degree 3 and node type DB), and containing 8 nodes, with a maximal depth of 2, which are 272797 (node type DB), bonnet00query (node type DB), heidemann01building (degree 3 and node type IR), chen00algebraic (node type DB) and dekhtyar99probabilistic (node type DB). Its nodes have 3 node types, which are DB (4 nodes, 0.12%), IR (2 nodes, 0.06%) and Agents.

- Tree starting from the root node kim01secret (degree 2), and containing 7 nodes, with a maximal depth of 3, which are 467998, vagina03cryptographic (degree 4), 482071, 496883 and tan01trust. Its nodes have a single node type, which is Agents.
- Tree starting from the root node cadoli98survey (degree 2 and node type AI), and containing 6 nodes, with a maximal depth of 2, which are 83444 (degree 4 and node type AI), prendinger00hyper (node type DB), 210930 (node type AI), bellardo00implementing (node type AI) and eiter00difference (node type DB). Its nodes have 2 node types, which are AI (3 nodes, 0.09%) and DB (2 nodes, 0.06%).
- Tree starting from the root node das98rule (degree 2 and node type ML), and containing 6 nodes, with a maximal depth of 2, which are 169000 (degree 4 and node type DB), 74893 (node type AI), 469106 (node type DB), 534720 (node type DB) and rodriguez00learning (node type DB). Its nodes have 2 node types, which are DB (4 nodes, 0.12%) and AI.
- Tree starting from the root node sampaio98deductive (degree 2), and containing 5 nodes, with a maximal depth of 2, which are murray98kaleidoquery (degree 3), sampaio00design, 116696 and fegaras99voodoo. Its nodes have a single node type, which is DB.

And another tree.

Dendritic trees A dendritic tree is a tree-like structure starting from a root node that is part of another strongly connected component. We have detected 44 dendritic trees in the graph, involving a total of 291 nodes (8.79%) and 291 edges (3.16%), with the largest one involving 26 nodes and 26 edges. The detected dendritic trees, sorted by decreasing size, are:

- Dendritic tree starting from the root node rao95bdi (degree 51 and node type Agents), and containing 26 nodes, with a maximal depth of 4, which are 243827 (node type Agents), 257383 (node type Agents), 270678 (node type Agents), 318212 (node type Agents) and 445758 (node type Agents). Its nodes have 3 node types, which are Agents (23 nodes, 0.69%), AI (2 nodes, 0.06%) and IR.
- Dendritic tree starting from the root node brin98anatomy (degree 99 and node type IR), and containing 21 nodes, with a maximal depth of 5, which are 128239 (node type IR), 165504 (node type IR), 500641 (node type IR), 520488 (node type IR) and 528932 (degree 3 and node type IR). Its nodes have 4 node types, which are IR (14 nodes, 0.42%), ML (5 nodes, 0.15%), DB and HCI.
- Dendritic tree starting from the root node essa99computer (degree 3 and node type HCI), and containing 14 nodes, with a maximal depth of 4, which are 24549 (degree 9 and node type DB), 28031 (node type ML), baker00hallucinating (node type ML), martinez00recognition (node type ML) and moghaddam98beyond (degree 4 and node type DB). Its nodes have 3 node types, which are ML (10 nodes, 0.30%), DB (3 nodes, 0.09%) and HCI.
- Dendritic tree starting from the root node howe97savvysearch (degree 21 and node type IR), and containing 12 nodes, with a maximal depth of 7, which are scime01websifter (node type IR), tzitzikas01democratic (node type IR), 496354 (node type DB), 537920 (node type IR) and mcilraith01semantic (degree 3 and node type IR). Its nodes have 5 node types, which are IR (5 nodes, 0.15%), DB (4 nodes, 0.12%), Agents, ML and HCI.
- Dendritic tree starting from the root node 90507 (degree 10 and node type IR), and containing 12 nodes, with a maximal depth of 2, which are cunningham01developing (degree 4 and node type IR), he00comparative (node type IR), itskevitch01automatic (degree 7 and node type IR), cunningham99experience (node type IR) and gaizauskas98information (node type IR). Its nodes have 2 node types, which are IR (10 nodes, 0.30%) and DB (2 nodes, 0.06%).
- Dendritic tree starting from the root node bergamaschi99semantic (degree 11 and node type DB), and containing 12 nodes, with a maximal depth of 5, which are 254597 (degree 5 and node type IR), 311175 (node type DB), 471747 (node type IR), 496736 (node type IR) and feng01towards (degree 4 and node type IR). Its nodes have 2 node types, which are IR (8 nodes, 0.24%) and DB (4 nodes, 0.12%).

And other 38 dendritic trees.

Stars A star is a tree with a maximal depth of one, where nodes with maximal unique degree one are connected to a central root node with a high degree. We have detected 65 stars in the graph, involving a total of 207 nodes (6.25%) and 142 edges (1.54%), with the largest one involving 5 nodes and 4 edges. The detected stars, sorted by decreasing size, are:

- Star starting from the root node white98towards (degree 4), and containing 5 nodes, with a maximal depth of 1, which are 205160, 409610, ferguson95role and wittner00network. Its nodes have a single node type, which is Agents.
- Star starting from the root node 196348 (degree 3), and containing 4 nodes, with a maximal depth of 1, which are 263968, 63224 and sengupta99constructing. Its nodes have a single node type, which is DB.
- Star starting from the root node degaris99building (degree 3), and containing 4 nodes, with a maximal depth of 1, which are 261630, degaris00simulating and degaris99evolving. Its nodes have a single node type, which is ML.
- Star starting from the root node markatos99caching (degree 3 and node type DB), and containing 4 nodes, with a maximal depth of 1, which are glance00community (node type HCI), markatos98effective (node type IR) and xie02locality (node type IR). Its nodes have 2 node types, which are IR (2 nodes, 0.06%) and HCI.
- Star starting from the root node takahashi00location (degree 3 and node type IR), and containing 4 nodes, with a maximal depth of 1, which are 539969 (node type IR), ishida99digital (node type Agents) and takahashi98mobile (node type IR). Its nodes have 2 node types, which are IR (2 nodes, 0.06%) and Agents.
- Star starting from the root node timm01synthesis (degree 3 and node type Agents), and containing 4 nodes, with a maximal depth of 1, which are timm00multiagent (node type IR), timm01enterprise (node type Agents) and toenshoff01flexible (node type Agents). Its nodes have 2 node types, which are Agents (2 nodes, 0.06%) and IR.

And other 59 stars.

Dendritic stars A dendritic star is a dendritic tree with a maximal depth of one, where nodes with maximal unique degree one are connected to a central root node with high degree and inside a strongly connected component. We have detected 61 dendritic stars in the graph, involving a total of 137 nodes (4.14%) and 137 edges (1.49%), with the largest one involving 5 nodes and 5 edges. The detected dendritic stars, sorted by decreasing size, are:

- Dendritic star starting from the root node liu98relationlog (degree 11), and containing 5 nodes, with a maximal depth of 1, which are 82903, fraternali98proceedings, liu01rulebased, liu98logical and liu99partial. Its nodes have a single node type, which is DB.
- Dendritic star starting from the root node decker95environment (degree 17 and node type Agents), and containing 5 nodes, with a maximal depth of 1, which are bilgic97risk (node type Agents), bilgic97system (node type Agents), decker98coordinating (node type Agents), obrst97constraints (node type Agents) and prasad96offline (node type ML). Its nodes have 2 node types, which are Agents (4 nodes, 0.12%) and ML.
- Dendritic star starting from the root node 78547 (degree 10 and node type IR), and containing 4 nodes, with a maximal depth of 1, which are 194227 (node type IR), ahanger99technique (node type IR), jaimes00integrating (node type ML) and slaughter00open (node type IR). Its nodes have 2 node types, which are IR (3 nodes, 0.09%) and ML.
- Dendritic star starting from the root node schattenberg00planning (degree 6 and node type Agents), and containing 3 nodes, with a maximal depth of 1, which are kitano99robocup (node type HCI), logan00distributed (node type Agents) and rintanen98planning (node type AI). Its nodes have 3 node types, which are HCI, Agents and AI.

- Dendritic star starting from the root node khaled98gado (degree 7), and containing 3 nodes, with a maximal depth of 1, which are bourdeau99three, davison98applying and rasheed98adaptive. Its nodes have a single node type, which is ML.
- Dendritic star starting from the root node godfrey98integrity (degree 5 and node type DB), and containing 3 nodes, with a maximal depth of 1, which are 71092 (node type DB), chan99possible (node type DB) and godfrey97minimization (node type IR). Its nodes have 2 node types, which are DB (2 nodes, 0.06%) and IR.

And other 55 dendritic stars.

Dendritic tendril stars A dendritic tendril star is a dendritic tree with a depth greater than one, where the arms of the star are tendrils. We have detected 23 dendritic tendril stars in the graph, involving a total of 93 nodes (2.81%) and 93 edges (1.01%), with the largest one involving 7 nodes and 7 edges. The detected dendritic tendril stars, sorted by decreasing size, are:

- Dendritic tendril star starting from the root node aha91casebased (degree 21), and containing 7 nodes, with a maximal depth of 2, which are 5234, 75123, 77029, mair99investigation and petrak95objectoriented. Its nodes have a single node type, which is ML.
- Dendritic tendril star starting from the root node holtman98automatic (degree 6), and containing 6 nodes, with a maximal depth of 3, which are 300668, 340027, schaller99objectivitydb, stockinger01design and 35804. Its nodes have a single node type, which is DB.
- Dendritic tendril star starting from the root node papadopoulos00models (degree 7 and node type HCI), and containing 5 nodes, with a maximal depth of 3, which are 532291 (node type Agents), chen99dynamic (node type Agents), skarmeas99component (node type Agents), bardram97plans (node type HCI) and reddy01coordinating (node type HCI). Its nodes have 2 node types, which are Agents (3 nodes, 0.09%) and HCI (2 nodes, 0.06%).
- Dendritic tendril star starting from the root node beigi97metaseek (degree 10 and node type IR), and containing 5 nodes, with a maximal depth of 2, which are 291240 (node type HCI), 305534 (node type ML), 420817 (node type HCI), laaksonen99picsom (node type IR) and koskela00picsom (node type HCI). Its nodes have 3 node types, which are HCI (3 nodes, 0.09%), ML and IR.
- Dendritic tendril star starting from the root node langley95applications (degree 6 and node type ML), and containing 5 nodes, with a maximal depth of 3, which are 162298 (node type ML), giraud-carrier98beyond (node type AI), lau00version (node type ML), 415731 (node type IR) and lau99programming (node type ML). Its nodes have 3 node types, which are ML (3 nodes, 0.09%), AI and IR.
- Dendritic tendril star starting from the root node thomas98wearable (degree 6), and containing 4 nodes, with a maximal depth of 3, which are 503243, 534400, ockerman98preliminary and pentland99digital. Its nodes have a single node type, which is HCI.

And other 17 dendritic tendril stars.

Free-floating chains A free-floating chain is a tree with maximal degree two. We have detected 15 free-floating chains in the graph, involving a total of 65 nodes (1.96%) and 50 edges (0.54%), with the largest one involving 7 nodes and 6 edges. The detected free-floating chains, sorted by decreasing size, are:

- Free-floating chain starting from the root node mcroy95repair (degree 5 and node type Agents), and containing 7 nodes, with a maximal depth of 2, which are 3489 (node type AI), ardissono00plan (node type Agents), ardissono96uso (node type Agents), mcroy98achieving (node type AI) and traum99speech (node type Agents). Its nodes have 2 node types, which are Agents (4 nodes, 0.12%) and AI (2 nodes, 0.06%).
- Free-floating chain starting from the root node liu00extended (degree 3 and node type ML), and containing 5 nodes, with a maximal depth of 2, which are 521000 (node type ML), frank98generating

(node type ML), hekanaho98dogma (node type AI) and fertig99fuzzy (node type ML). Its nodes have 2 node types, which are ML (3 nodes, 0.09%) and AI.

- Free-floating chain starting from the root node hatzilygeroudis00neurules (degree 4), and containing 5 nodes, with a maximal depth of 2, which are hatzilygeroudis02multiinference, prentzas01webbased, prentzas02webbased and hatzilygeroudis01hymes. Its nodes have a single node type, which is AI.
- Free-floating chain starting from the root node chen98learningbased (degree 2), and containing 4 nodes, with a maximal depth of 2, which are sreerupa98dynamic, weng98visionguided and 208646. Its nodes have a single node type, which is ML.
- Free-floating chain starting from the root node 288424 (degree 2), and containing 4 nodes, with a maximal depth of 2, which are granlund01patternsupported, vanwelie00patterns and borchers00pattern. Its nodes have a single node type, which is HCI.
- Free-floating chain starting from the root node 461740 (degree 2 and node type ML), and containing 4 nodes, with a maximal depth of 2, which are 149759 (node type AI), nguyen98strict (node type AI) and 496719 (node type ML). Its nodes have 2 node types, which are AI (2 nodes, 0.06%) and ML.

And other 9 free-floating chains.

Tendrils A tendril is a path starting from a node of degree one, connected to a strongly connected component. We have detected 240 tendrils in the graph, involving a total of 321 nodes (9.69%) and 321 edges (3.49%), with the largest one involving 3 nodes and 3 edges. The detected tendrils, sorted by decreasing size, are:

- Tendril starting from the root node bharat99comparison (degree 5), and containing 3 nodes, with a maximal depth of 3, which are 502499, almeida01analyzing and heinonen96www. Its nodes have a single node type, which is IR.
- Tendril starting from the root node kubiatowicz00oceanstore (degree 4 and node type HCI), and containing 3 nodes, with a maximal depth of 3, which are 525023 (node type HCI), grimm01systems (node type Agents) and jennings01aspects (node type HCI). Its nodes have 2 node types, which are HCI (2 nodes, 0.06%) and Agents.
- Tendril starting from the root node stolzenburg01from (degree 3 and node type Agents), and containing 3 nodes, with a maximal depth of 3, which are 335912 (node type ML), boutilier01partialorder (node type Agents) and brafman98knowledge (node type AI). Its nodes have 3 node types, which are ML, Agents and AI.
- Tendril starting from the root node 35592 (degree 5), and containing 3 nodes, with a maximal depth of 3, which are 69807, onoda98asymptotic and 12247. Its nodes have a single node type, which is ML.
- Tendril starting from the root node 242172 (degree 10), and containing 3 nodes, with a maximal depth of 3, which are rao96agentspeakl, hindriks00architecture and 491166. Its nodes have a single node type, which is Agents.
- Tendril starting from the root node oviatt99ten (degree 4), and containing 3 nodes, with a maximal depth of 3, which are 443913, conati00toward and 452812. Its nodes have a single node type, which is HCI.

And other 234 tendrils.

3.3.3 Cora

The undirected graph Cora has 2.71K heterogeneous nodes and 5.28K edges. The graph contains 78 connected components, with the largest one containing 2.48K nodes and the smallest one containing 2 nodes. The RAM requirements for the nodes and edges data structures are 207.68KB and 16.34KB respectively.

Degree centrality The minimum node degree is 1, the maximum node degree is 168, the mode degree is 2, the mean degree is 3.90 and the node degree median is 3. The nodes with the highest degree centrality are 35 (degree 168 and node type Genetic_Algorithms), 6213 (degree 78 and node type Reinforcement_Learning), 1365 (degree 74 and node type Neural_Networks), 3229 (degree 65 and node type Neural_Networks) and 910 (degree 44 and node type Neural_Networks).

Node types The graph has 7 node types, which are Neural_Networks (818 nodes, 30.21%), Probabilistic_Methods (426 nodes, 15.73%), Genetic_Algorithms (418 nodes, 15.44%), Theory (351 nodes, 12.96%), Case_Based (298 nodes, 11.00%), Reinforcement_Learning (217 nodes, 8.01%) and Rule_Learning (180 nodes, 6.65%). The RAM requirement for the node types data structure is 141.72KB.

Topological Oddities A topological oddity is a set of nodes in the graph that *may be derived* by an error during the generation of the edge list of the graph and, depending on the task, could bias the results of topology-based models. In the following paragraph, we will describe the detected topological oddities.

Node tuples A node tuple is a connected component composed of two nodes. We have detected 57 node tuples in the graph, involving a total of 114 nodes (4.21%) and 57 edges (0.54%). The detected node tuples are:

- Node tuple containing the nodes 1105622 (node type Neural_Networks) and 430574 (node type Neural_Networks).
- Node tuple containing the nodes 116512 (node type Neural_Networks) and 1107808 (node type Neural_Networks).
- Node tuple containing the nodes 1107728 (node type Neural_Networks) and 115188 (node type Neural_Networks).
- Node tuple containing the nodes 1136040 (node type Neural_Networks) and 754594 (node type Neural_Networks).
- Node tuple containing the nodes 73972 (node type Case_Based) and 50980 (node type Case_Based).
- Node tuple containing the nodes 628458 (node type Neural_Networks) and 628459 (node type Neural_Networks).
- Node tuple containing the nodes 180301 (node type Probabilistic_Methods) and 1110628 (node type Probabilistic_Methods).
- Node tuple containing the nodes 1133008 (node type Neural_Networks) and 688824 (node type Neural_Networks).
- Node tuple containing the nodes 654519 (node type Genetic_Algorithms) and 1131754 (node type Genetic_Algorithms).
- Node tuple containing the nodes 49720 (node type Probabilistic_Methods) and 49753 (node type Probabilistic_Methods).
- Node tuple containing the nodes 133628 (node type Theory) and 1108570 (node type Theory).
- Node tuple containing the nodes 617378 (node type Neural_Networks) and 1130069 (node type Neural_Networks).
- Node tuple containing the nodes 529165 (node type Neural_Networks) and 1126315 (node type Neural_Networks).
- Node tuple containing the nodes 824245 (node type Neural_Networks) and 1139009 (node type Neural_Networks).
- Node tuple containing the nodes 820661 (node type Neural_Networks) and 817774 (node type Neural_Networks).

And other 42 node tuples.

Isomorphic node groups Isomorphic groups are nodes with exactly the same neighbours and node types (if present in the graph). Nodes in such groups are topologically indistinguishable, that is swapping their ID would not change the graph topology. We have detected 3 isomorphic node groups in the graph, involving a total of 6 nodes (0.22%) and 30 edges (0.28%). The detected isomorphic node groups, sorted by decreasing size, are:

- Group with 2 nodes (degree 5 and node type Genetic_Algorithms): 1104999 and 63832.
- Group with 2 nodes (degree 5 and node type Neural_Networks): 43698 and 31336.
- Group with 2 nodes (degree 5 and node type Neural_Networks): 1154123 and 1154124.

Dendritic trees A dendritic tree is a tree-like structure starting from a root node that is part of another strongly connected component. We have detected 13 dendritic trees in the graph, involving a total of 64 nodes (2.36%) and 64 edges (0.61%), with the largest one involving 9 nodes and 9 edges. The detected dendritic trees, sorted by decreasing size, are:

- Dendritic tree starting from the root node 16819 (degree 14), and containing 9 nodes, with a maximal depth of 4, which are 1131274, 643003, 644843, 1131189 and 645016 (degree 5). Its nodes have a single node type, which is Probabilistic_Methods.
- Dendritic tree starting from the root node 35 (degree 168), and containing 7 nodes, with a maximal depth of 2, which are 1152508, 1137466, 1128945, 1119505 and 15670. Its nodes have a single node type, which is Genetic_Algorithms.
- Dendritic tree starting from the root node 16437 (degree 6), and containing 6 nodes, with a maximal depth of 3, which are 51831, 430329, 127940 (degree 4), 416964 and 1114364. Its nodes have a single node type, which is Neural_Networks.
- Dendritic tree starting from the root node 424540 (degree 3), and containing 5 nodes, with a maximal depth of 3, which are 18536 (degree 3), 1106854, 86923 (degree 3), 18532 and 1114184. Its nodes have a single node type, which is Neural_Networks.
- Dendritic tree starting from the root node 910 (degree 44 and node type Neural_Networks), and containing 5 nodes, with a maximal depth of 2, which are 94953 (node type Neural_Networks), 1122460 (node type Neural_Networks), 1114118 (node type Neural_Networks), 245288 (node type Reinforcement_Learning) and 119712 (node type Genetic_Algorithms). Its nodes have 3 node types, which are Neural_Networks (3 nodes, 0.11%), Reinforcement_Learning and Genetic_Algorithms.
- Dendritic tree starting from the root node 13885 (degree 7 and node type Neural_Networks), and containing 5 nodes, with a maximal depth of 3, which are 84459 (node type Theory), 6238 (degree 4 and node type Theory), 1123991 (node type Probabilistic_Methods), 10793 (node type Theory) and 1130356 (node type Theory). Its nodes have 2 node types, which are Theory (4 nodes, 0.15%) and Probabilistic_Methods.

And other 7 dendritic trees.

Stars A star is a tree with a maximal depth of one, where nodes with maximal unique degree one are connected to a central root node with a high degree. We have detected 3 stars in the graph, involving a total of 9 nodes (0.33%) and 6 edges (0.06%). The detected stars are:

- Star starting from the root node 1112071 (degree 2), and containing 3 nodes, with a maximal depth of 1, which are 212107 and 212097. Its nodes have a single node type, which is Probabilistic_Methods.
- Star starting from the root node 1123215 (degree 2), and containing 3 nodes, with a maximal depth of 1, which are 288107 and 149139. Its nodes have a single node type, which is Theory.

- Star starting from the root node 9559 (degree 2), and containing 3 nodes, with a maximal depth of 1, which are 1102794 and 252725. Its nodes have a single node type, which is Rule_Learning.

Dendritic stars A dendritic star is a dendritic tree with a maximal depth of one, where nodes with maximal unique degree one are connected to a central root node with high degree and inside a strongly connected component. We have detected 29 dendritic stars in the graph, involving a total of 84 nodes (3.10%) and 84 edges (0.80%), with the largest one involving 12 nodes and 12 edges. The detected dendritic stars, sorted by decreasing size, are:

- Dendritic star starting from the root node 1365 (degree 74 and node type Neural_Networks), and containing 12 nodes, with a maximal depth of 1, which are 1105062 (node type Reinforcement_Learning), 853150 (node type Neural_Networks), 949318 (node type Neural_Networks), 1136442 (node type Neural_Networks) and 1132922 (node type Neural_Networks). Its nodes have 2 node types, which are Neural_Networks (11 nodes, 0.41%) and Reinforcement_Learning.
- Dendritic star starting from the root node 20193 (degree 23), and containing 11 nodes, with a maximal depth of 1, which are 1153877, 1153879, 1153889, 1130653 and 1130657. Its nodes have a single node type, which is Case_Based.
- Dendritic star starting from the root node 6913 (degree 12), and containing 4 nodes, with a maximal depth of 1, which are 1105011, 1131230, 703953 and 646289. Its nodes have a single node type, which is Probabilistic_Methods.
- Dendritic star starting from the root node 205196 (degree 9), and containing 4 nodes, with a maximal depth of 1, which are 628766, 1130568, 1130586 and 815073. Its nodes have a single node type, which is Neural_Networks.
- Dendritic star starting from the root node 89547 (degree 13 and node type Theory), and containing 3 nodes, with a maximal depth of 1, which are 1152379 (node type Theory), 1116328 (node type Neural_Networks) and 237376 (node type Theory). Its nodes have 2 node types, which are Theory (2 nodes, 0.07%) and Neural_Networks.
- Dendritic star starting from the root node 31353 (degree 19), and containing 3 nodes, with a maximal depth of 1, which are 286562, 1063773 and 686559. Its nodes have a single node type, which is Neural_Networks.

And other 23 dendritic stars.

Dendritic tendril stars A dendritic tendril star is a dendritic tree with a depth greater than one, where the arms of the star are tendrils. We have detected 7 dendritic tendril stars in the graph, involving a total of 28 nodes (1.03%) and 28 edges (0.27%), with the largest one involving 6 nodes and 6 edges. The detected dendritic tendril stars, sorted by decreasing size, are:

- Dendritic tendril star starting from the root node 3229 (degree 65 and node type Neural_Networks), and containing 6 nodes, with a maximal depth of 3, which are 919885 (node type Neural_Networks), 1125082 (node type Genetic_Algorithms), 7022 (node type Neural_Networks), 1112767 (node type Neural_Networks) and 226698 (node type Neural_Networks). Its nodes have 2 node types, which are Neural_Networks (5 nodes, 0.18%) and Genetic_Algorithms.
- Dendritic tendril star starting from the root node 643069 (degree 4 and node type Probabilistic_Methods), and containing 6 nodes, with a maximal depth of 5, which are 14090 (node type Probabilistic_Methods), 1131192 (node type Probabilistic_Methods), 1103016 (node type Neural_Networks), 14083 (node type Neural_Networks) and 62676 (node type Neural_Networks). Its nodes have 2 node types, which are Neural_Networks (4 nodes, 0.15%) and Probabilistic_Methods (2 nodes, 0.07%).
- Dendritic tendril star starting from the root node 3243 (degree 12 and node type Theory), and containing 4 nodes, with a maximal depth of 3, which are 1103610 (node type Theory), 854434 (node type Neural_Networks), 8961 (node type Reinforcement_Learning) and 1133390 (node type Theory).

Its nodes have 3 node types, which are Theory (2 nodes, 0.07%), Neural_Networks and Reinforcement_Learning.

- Dendritic tendril star starting from the root node 5086 (degree 12), and containing 3 nodes, with a maximal depth of 2, which are 354004, 1105698 and 1118546. Its nodes have a single node type, which is Probabilistic_Methods.
- Dendritic tendril star starting from the root node 35863 (degree 5 and node type Reinforcement_Learning), and containing 3 nodes, with a maximal depth of 2, which are 28359 (node type Reinforcement_Learning), 134060 (node type Theory) and 481073 (node type Reinforcement_Learning). Its nodes have 2 node types, which are Reinforcement_Learning (2 nodes, 0.07%) and Theory.
- Dendritic tendril star starting from the root node 162080 (degree 5), and containing 3 nodes, with a maximal depth of 2, which are 738941, 1135345 and 1135455. Its nodes have a single node type, which is Neural_Networks.

And another dendritic tendril star.

Free-floating chains A free-floating chain is a tree with maximal degree two. We have detected 2 free-floating chains in the graph, involving a total of 8 nodes (0.30%) and 6 edges (0.06%). The detected free-floating chains are:

- Free-floating chain starting from the root node 375825 (degree 2), and containing 4 nodes, with a maximal depth of 2, which are 1119623, 421481 and 111770. Its nodes have a single node type, which is Probabilistic_Methods.
- Free-floating chain starting from the root node 430711 (degree 2), and containing 4 nodes, with a maximal depth of 2, which are 671052, 1132416 and 1132406. Its nodes have a single node type, which is Neural_Networks.

Tendrils A tendril is a path starting from a node of degree one, connected to a strongly connected component. We have detected 224 tendrils in the graph, involving a total of 265 nodes (9.79%) and 265 edges (2.51%), with the largest one involving 4 nodes and 4 edges. The detected tendrils, sorted by decreasing size, are:

- Tendril starting from the root node 83847 (degree 4), and containing 4 nodes, with a maximal depth of 4, which are 1130678, 630890, 233106 and 12275. Its nodes have a single node type, which is Neural_Networks.
- Tendril starting from the root node 1140543 (degree 5), and containing 3 nodes, with a maximal depth of 3, which are 120817, 1109873 and 163235. Its nodes have a single node type, which is Neural_Networks.
- Tendril starting from the root node 683404 (degree 4), and containing 3 nodes, with a maximal depth of 3, which are 683360, 522338 and 1132864. Its nodes have a single node type, which is Probabilistic_Methods.
- Tendril starting from the root node 20534 (degree 10 and node type Reinforcement_Learning), and containing 3 nodes, with a maximal depth of 3, which are 13972 (node type Reinforcement_Learning), 1126050 (node type Reinforcement_Learning) and 93318 (node type Neural_Networks). Its nodes have 2 node types, which are Reinforcement_Learning (2 nodes, 0.07%) and Neural_Networks.
- Tendril starting from the root node 66751 (degree 5), and containing 3 nodes, with a maximal depth of 3, which are 1138043, 77108 and 77112. Its nodes have a single node type, which is Theory.
- Tendril starting from the root node 3231 (degree 36 and node type Theory), and containing 2 nodes, with a maximal depth of 2, which are 1113926 (node type Neural_Networks) and 250566 (node type Case_Based). Its nodes have 2 node types, which are Neural_Networks and Case_Based.

And other 218 tendrils.

4 Models used in edge & node-label prediction experiments

In this section we report details about the *Embiggen* models, their hyper-parameters and results obtained in the edge and node label prediction experiments.

4.1 Evaluated node embedding models

In the following section we report the node embedding model parameters used within the context of the *GRAPE* pipelines for the evaluation of models on node-label and edge prediction tasks.

4.1.1 Node2Vec CBOW

The parameters used for the node embedding model *Node2Vec CBOW* in *GRAPE* evaluation pipelines for the node-label and edge prediction tasks are reported in Supplementary Table 2.

Supplementary Table 2: Node2Vec CBOW model parameters.

Parameter name	Value
Embedding size	100
Epochs	10
Number of negative samples	10
Walk length	128
Iterations	10
Window size	10
Return weight	0.25
Explore weight	4
Max neighbours	100
Learning rate	0.01
Learning rate decay	0.9

4.1.2 Node2Vec GloVe

The parameters used for the node embedding model *Node2Vec GloVe* in *GRAPE* evaluation pipelines for the node-label and edge prediction tasks are reported in Supplementary Table 3.

Supplementary Table 3: Node2Vec GloVe model parameters.

Parameter name	Value
Embedding size	100
Alpha	0.75
Walk length	128
Iterations	10
Window size	10
Return weight	0.25
Explore weight	4
Max neighbours	100
Epochs	500
Learning rate	0.01
Learning rate decay	0.9

4.1.3 Node2Vec SkipGram

The parameters used for the node embedding model *Node2Vec SkipGram* in *GRAPE* evaluation pipelines for the node-label and edge prediction tasks are reported in Supplementary Table 4.

Supplementary Table 4: Node2Vec SkipGram model parameters.

Parameter name	Value
Embedding size	100
Epochs	10
Number of negative samples	10
Walk length	128
Iterations	10
Window size	10
Return weight	0.25
Explore weight	4
Max neighbours	100
Learning rate	0.01
Learning rate decay	0.9

4.1.4 DeepWalk CBOW

The parameters used for the node embedding model *DeepWalk CBOW* in *GRAPE* evaluation pipelines for the node-label and edge prediction tasks are reported in Supplementary Table 5.

Supplementary Table 5: DeepWalk CBOW model parameters.

Parameter name	Value
Embedding size	100
Epochs	10
Number of negative samples	10
Walk length	128
Iterations	10
Window size	10
Max neighbours	100
Learning rate	0.01
Learning rate decay	0.9

4.1.5 DeepWalk GloVe

The parameters used for the node embedding model *DeepWalk GloVe* in *GRAPE* evaluation pipelines for the node-label and edge prediction tasks are reported in Supplementary Table 6.

Supplementary Table 6: DeepWalk GloVe model parameters.

Parameter name	Value
Embedding size	100
Alpha	0.75
Walk length	128
Iterations	10
Window size	10
Max neighbours	100
Epochs	500
Learning rate	0.01
Learning rate decay	0.9

4.1.6 DeepWalk SkipGram

The parameters used for the node embedding model *DeepWalk SkipGram* in *GRAPE* evaluation pipelines for the node-label and edge prediction tasks are reported in Supplementary Table 7.

Supplementary Table 7: DeepWalk SkipGram model parameters.

Parameter name	Value
Embedding size	100
Epochs	10
Number of negative samples	10
Walk length	128
Iterations	10
Window size	10
Max neighbours	100
Learning rate	0.01
Learning rate decay	0.9

4.1.7 First Order LINE

The parameters used for the node embedding model *First Order LINE* in *GRAPE* evaluation pipelines for the node-label and edge prediction tasks are reported in Supplementary Table 8.

Supplementary Table 8: First Order LINE model parameters.

Parameter name	Value
Embedding size	100
Epochs	500
Batch size	1024
Optimizer	nadam
Early stopping min delta	0.001
Early stopping patience	10
Learning rate plateau min delta	0.001
Learning rate plateau patience	5
Negative samples rate	0.5

4.1.8 Second Order LINE

The parameters used for the node embedding model *Second Order LINE* in *GRAPE* evaluation pipelines for the node-label and edge prediction tasks are reported in Supplementary Table 9.

Supplementary Table 9: Second Order LINE model parameters.

Parameter name	Value
Embedding size	100
Epochs	500
Batch size	1024
Optimizer	nadam
Early stopping min delta	0.001
Early stopping patience	10
Learning rate plateau min delta	0.001
Learning rate plateau patience	5
Negative samples rate	0.5

4.1.9 NMFADMM

The parameters used for the node embedding model *NMFADMM* in *GRAPE* evaluation pipelines for the node-label and edge prediction tasks are reported in Supplementary Table 10.

Supplementary Table 10: NMFADMM model parameters.

Parameter name	Value
Embedding size	128
Iterations	100
Rho	1

4.1.10 RandNE

The parameters used for the node embedding model *RandNE* in *GRAPE* evaluation pipelines for the node-label and edge prediction tasks are reported in Supplementary Table 11.

Supplementary Table 11: RandNE model parameters.

Parameter name	Value
Embedding size	100
Alphas	(0.5, 0.5)

4.1.11 GraRep

The parameters used for the node embedding model *GraRep* in *GRAPE* evaluation pipelines for the node-label and edge prediction tasks are reported in Supplementary Table 12.

Supplementary Table 12: GraRep model parameters.

Parameter name	Value
Embedding size	128
Iteration	10
Order	5

4.1.12 DeepWalk Walklets SkipGram

The parameters used for the node embedding model *Walklets SkipGram* in *GRAPE* evaluation pipelines for the node-label and edge prediction tasks are reported in Supplementary Table 13.

Supplementary Table 13: DeepWalk Walklets SkipGram model parameters.

Parameter name	Value
Embedding size	100
Epochs	10
Number of negative samples	10
Walk length	128
Iterations	10
Window size	10
Max neighbours	100
Learning rate	0.01
Learning rate decay	0.9

4.1.13 NetMF

The parameters used for the node embedding model *NetMF* in *GRAPE* evaluation pipelines for the node-label and edge prediction tasks are reported in Supplementary Table 14.

Supplementary Table 14: NetMF model parameters.

Parameter name	Value
Embedding size	100
Walk length	128
Iterations	10
Window size	10
Max neighbours	100

4.1.14 GLEE

The parameters used for the node embedding model *GLEE* in *GRAPE* evaluation pipelines for the node-label and edge prediction tasks are reported in Supplementary Table 15.

Supplementary Table 15: GLEE model parameters.

Parameter name	Value
Embedding size	100

4.1.15 HOPE

The parameters used for the node embedding model *HOPE* in *GRAPE* evaluation pipelines for the node-label and edge prediction tasks are reported in Supplementary Table 16.

Supplementary Table 16: HOPE model parameters.

Parameter name	Value
Embedding size	100
Metric	Neighbours Intersection size
Root node name	None

4.1.16 Role2Vec

The parameters used for the node embedding model *Role2Vec* in *GRAPE* evaluation pipelines for the node-label and edge prediction tasks are reported in Supplementary Table 17.

Supplementary Table 17: Role2Vec model parameters.

Parameter name	Value
Embedding size	100
Walk number	10
Walk length	80
Window size	5
Epochs	10
Learning rate	0.05
Min count	1
Down sampling	0.0001
Weisfeiler lehman hashing iterations	2
Erase base features	False

4.2 Evaluated edge prediction models

4.2.1 Decision Tree Classifier

The parameters used for the edge prediction model *Decision Tree Classifier* in *GRAPE* evaluation pipelines for the node-label and edge prediction tasks are reported in Supplementary Table 18.

Supplementary Table 18: Parameters of Decision Tree Classifier model for edge prediction.

Parameter name	Value
Edge embedding method	Hadamard
Training unbalance rate	1
Criterion	gini
Splitter	best
Max depth	10
Min samples split	2
Min samples leaf	1
Min weight fraction leaf	0
Max features	None
Max leaf nodes	None
Min impurity decrease	0
CCP alpha	0

4.2.2 Perceptron

The parameters used for the edge prediction model *Perceptron* in *GRAPE* evaluation pipelines for the node-label and edge prediction tasks are reported in Supplementary Table 19.

Supplementary Table 19: Parameters of Perceptron model for edge prediction.

Parameter name	Value
Edge embeddings	Hadamard
Cooccurrence iterations	100
Cooccurrence window size	10
Number of epochs	100
Number of edges per mini batch	256
Learning rate	0.001
First order decay factor	0.9
Second order decay factor	0.999

4.3 Evaluated node-label prediction models

4.3.1 Decision Tree Classifier

The parameters used for the node label prediction model *Decision Tree Classifier* in *GRAPE* evaluation pipelines for the node-label and edge prediction tasks are reported in Supplementary Table 20.

Supplementary Table 20: Parameters of Decision Tree Classifier model for node-label prediction.

Parameter name	Value
Criterion	gini
Splitter	best
Max depth	10
Min samples split	2
Min samples leaf	1
Min weight fraction leaf	0
Max features	None
Max leaf nodes	None
Min impurity decrease	0
Class weight	balanced
CCP alpha	0

4.3.2 Random Forest Classifier

The parameters used for the node label prediction model *Random Forest Classifier* in *GRAPE* evaluation pipelines for the node-label and edge prediction tasks are reported in Supplementary Table 21.

Supplementary Table 21: Parameters of Random Forest Classifier model for node-label prediction.

Parameter name	Value
N estimators	1000
Criterion	gini
Max depth	10
Min samples split	2
Min samples leaf	1
Min weight fraction leaf	0
Max features	sqrt
Max leaf nodes	None
Min impurity decrease	0
Bootstrap	True
Oob score	False
Warm start	False
Class weight	balanced
CCP alpha	0
Max samples	None

5 Edge and node label prediction performance

In this section, we report the full performance results, estimated with different metrics, using the *GRAPE* pipelines for the evaluation of edge and node-label prediction tasks.

5.1 Edge prediction performance

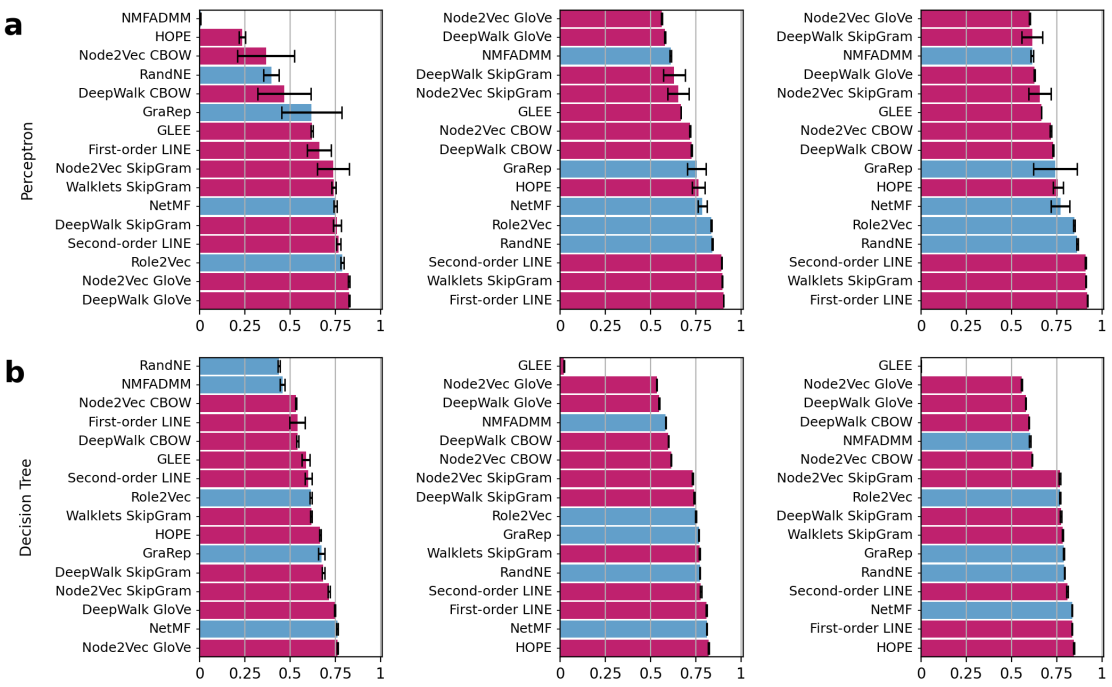

Supplementary Figure 1: **Average f1 score of edge prediction models trained on embedding methods.** Results are averaged across $n = 10$ holdouts. Data are presented as mean values $+/-$ SD. Embedding models are sorted for each task; methods implemented in *GRAPE* are in purple, while methods integrated through the *GRAPE* interface are in cyan. We show perceptron and decision tree in row **a** and **b**, respectively. From left to right, Human Phenotype Ontology, STRING Homo sapiens and STRING Mus musculus.

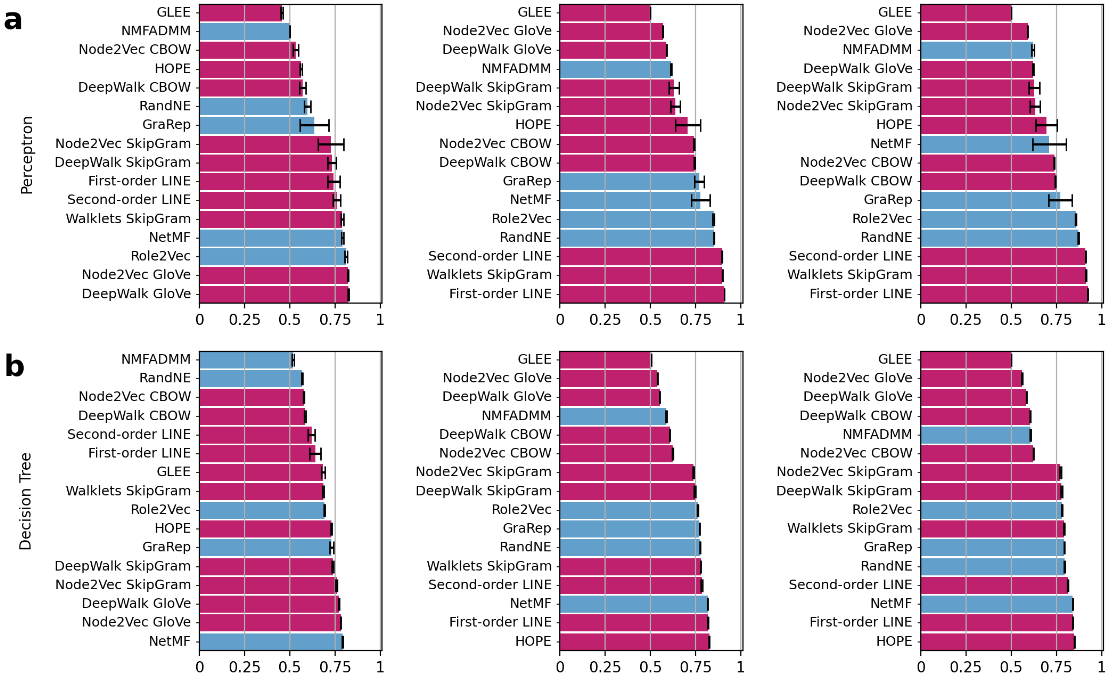

Supplementary Figure 2: **Average balanced accuracy of edge prediction models trained on embedding methods.** Results are averaged across $n = 10$ holdouts. Data are presented as mean values $+/-$ SD. Embedding models are sorted for each task; methods implemented in *GRAPE* are in purple, while methods integrated through the *GRAPE* interface are in cyan. We show perceptron and decision tree in row **a** and **b**, respectively. From left to right, Human Phenotype Ontology, STRING Homo sapiens and STRING Mus musculus.

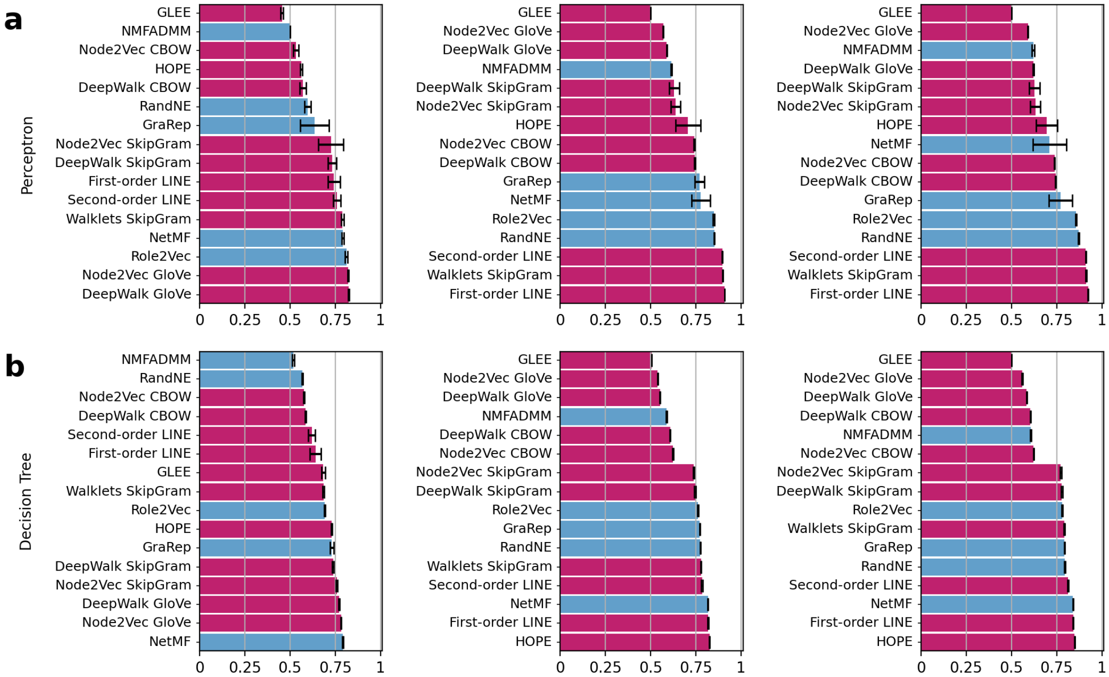

Supplementary Figure 3: **Average accuracy of edge prediction models trained on embedding methods.** Results are averaged across $n = 10$ holdouts. Data are presented as mean values $+/-$ SD. Embedding models are sorted for each task; methods implemented in *GRAPE* are in purple, while methods integrated through the *GRAPE* interface are in cyan. We show perceptron and decision tree in row **a** and **b**, respectively. From left to right, Human Phenotype Ontology, STRING Homo sapiens and STRING Mus musculus.

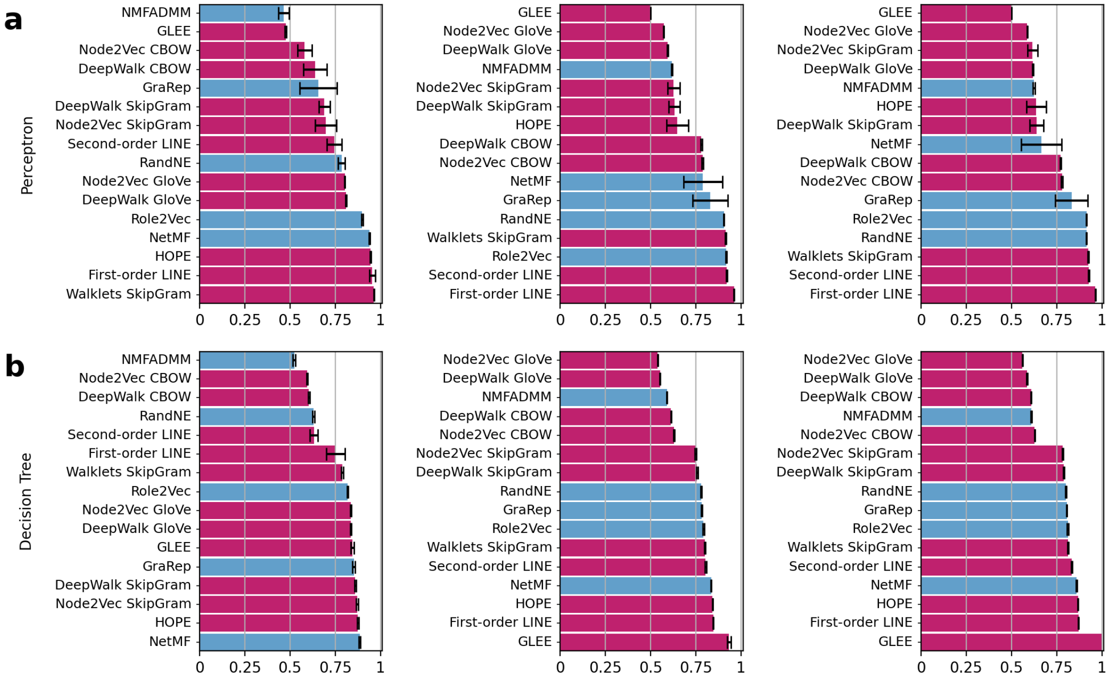

Supplementary Figure 4: **Average precision of edge prediction models trained on embedding methods.** Results are averaged across $n = 10$ holdouts. Data are presented as mean values +/− SD. Embedding models are sorted for each task; methods implemented in *GRAPE* are in purple, while methods integrated through the *GRAPE* interface are in cyan. We show perceptron and decision tree in row **a** and **b**, respectively. From left to right, Human Phenotype Ontology, STRING Homo sapiens and STRING Mus musculus.

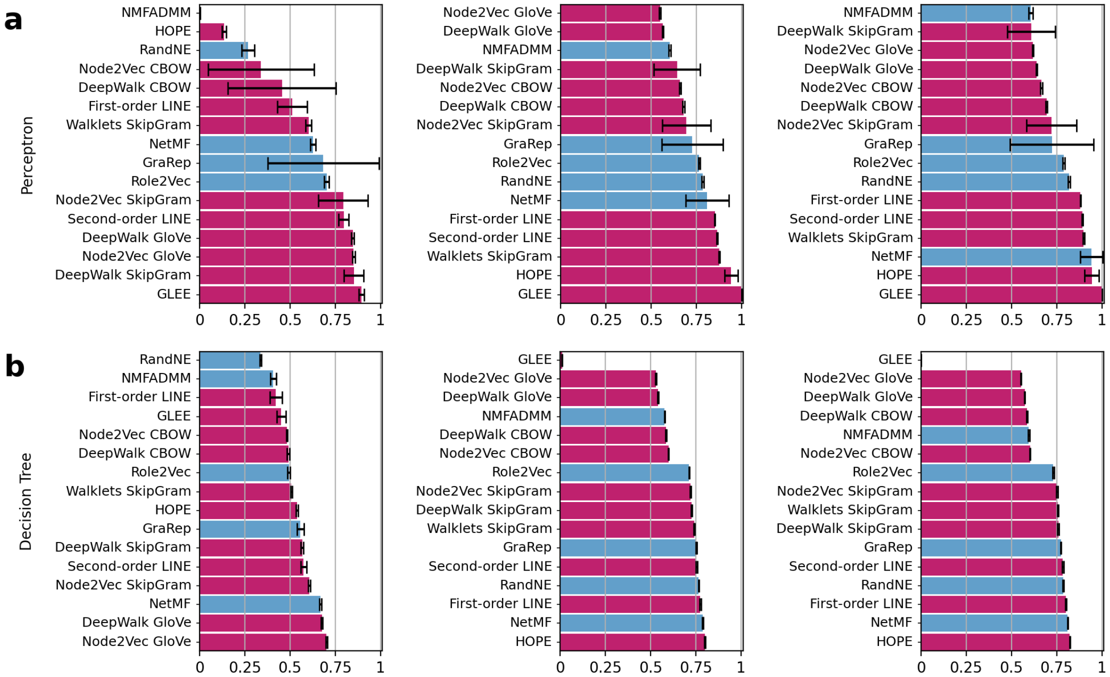

Supplementary Figure 5: **Average recall of edge prediction models trained on embedding methods.** Results are averaged across $n = 10$ holdouts. Data are presented as mean values $+/-$ SD. Embedding models are sorted for each task; methods implemented in *GRAPE* are in purple, while methods integrated through the *GRAPE* interface are in cyan. We show perceptron and decision tree in row **a** and **b**, respectively. From left to right, Human Phenotype Ontology, STRING Homo sapiens and STRING Mus musculus.

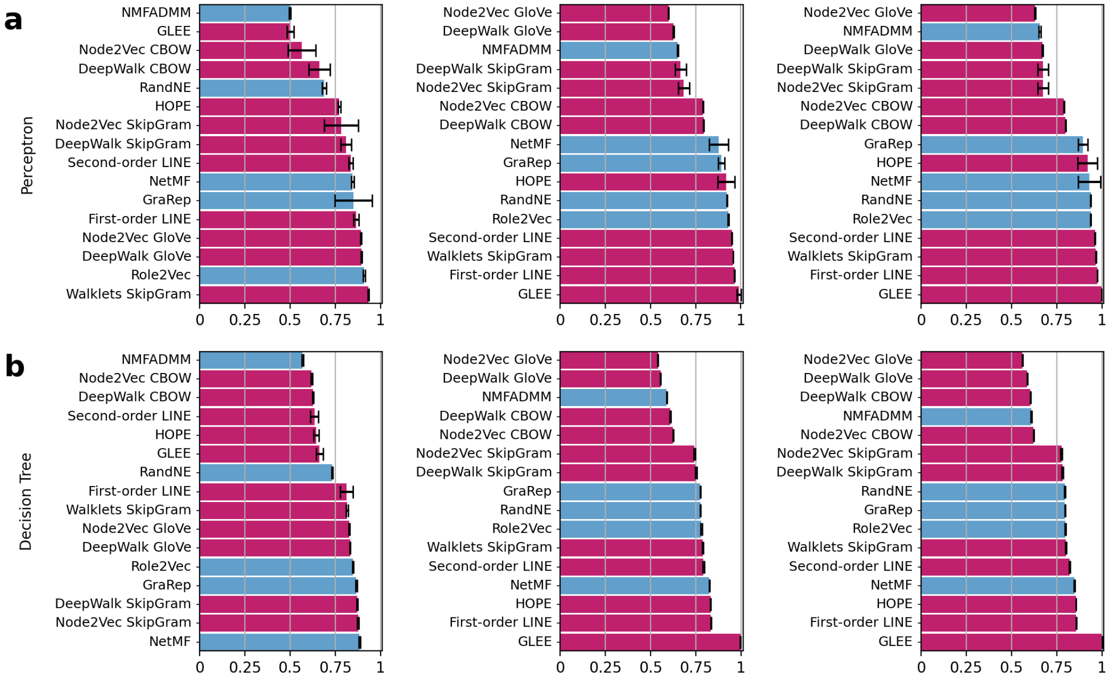

Supplementary Figure 6: **Average auroc of edge prediction models trained on embedding methods.** Results are averaged across $n = 10$ holdouts. Data are presented as mean values $+/-$ SD. Embedding models are sorted for each task; methods implemented in *GRAPE* are in purple, while methods integrated through the *GRAPE* interface are in cyan. We show perceptron and decision tree in row **a** and **b**, respectively. From left to right, Human Phenotype Ontology, STRING Homo sapiens and STRING Mus musculus.

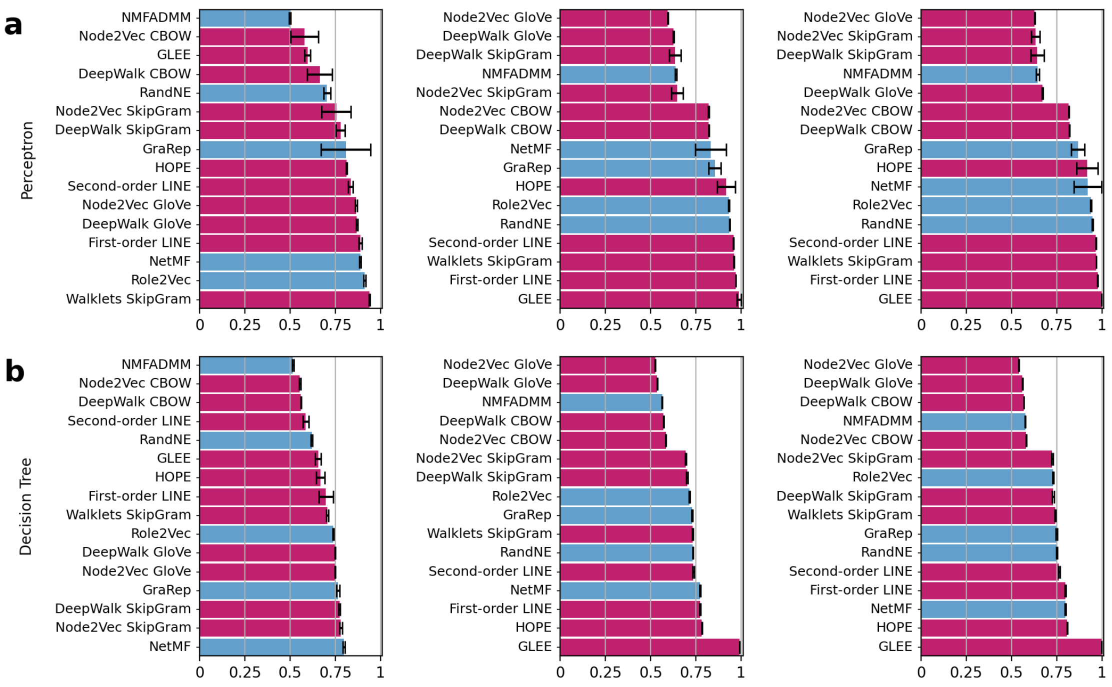

Supplementary Figure 7: **Average auprc of edge prediction models trained on embedding methods.** Results are averaged across $n = 10$ holdouts. Data are presented as mean values +/− SD. Embedding models are sorted for each task; methods implemented in *GRAPE* are in purple, while methods integrated through the *GRAPE* interface are in cyan. We show perceptron and decision tree in row **a** and **b**, respectively. From left to right, Human Phenotype Ontology, STRING Homo sapiens and STRING Mus musculus.

Supplementary Table 22: Decision Tree edge prediction performance in test evaluation on Human Phenotype Ontology. The rows are sorted by balanced accuracy.

	Balanced Accuracy	F1 Score	AUPRC	Accuracy	Recall	AUROC	Precision
NMFADMM	.52 ± .006	.46 ± .013	.52 ± .005	.52 ± .006	.41 ± .016	.57 ± .004	.52 ± .007
RandNE	.57 ± .003	.44 ± .005	.62 ± .004	.57 ± .003	.34 ± .005	.73 ± .004	.63 ± .006
Node2Vec CBOW	.58 ± .003	.53 ± .003	.55 ± .004	.58 ± .003	.48 ± .003	.62 ± .004	.60 ± .004
DeepWalk CBOW	.59 ± .004	.54 ± .006	.56 ± .002	.59 ± .004	.49 ± .008	.63 ± .003	.61 ± .004
Second-order LINE	.62 ± .019	.60 ± .018	.59 ± .017	.62 ± .019	.58 ± .015	.64 ± .022	.63 ± .023
First-order LINE	.64 ± .031	.54 ± .041	.70 ± .039	.64 ± .031	.42 ± .035	.81 ± .035	.75 ± .051
GLEE	.68 ± .01	.59 ± .022	.66 ± .015	.68 ± .01	.45 ± .026	.66 ± .018	.84 ± .009
Walklets	.69 ± .004	.62 ± .005	.71 ± .006	.69 ± .004	.51 ± .005	.82 ± .006	.79 ± .007
Role2Vec	.69 ± .004	.62 ± .006	.74 ± .004	.69 ± .004	.49 ± .008	.85 ± .003	.82 ± .004
HOPE	.73 ± .004	.67 ± .005	.67 ± .023	.73 ± .004	.54 ± .005	.64 ± .015	.88 ± .004
GraRep	.73 ± .01	.67 ± .016	.77 ± .01	.73 ± .01	.56 ± .02	.87 ± .004	.85 ± .006
DeepWalk SkipGram	.74 ± .004	.68 ± .006	.77 ± .004	.74 ± .004	.57 ± .007	.87 ± .003	.86 ± .004
Node2Vec SkipGram	.76 ± .005	.72 ± .006	.78 ± .007	.76 ± .005	.61 ± .006	.88 ± .005	.87 ± .006
DeepWalk GloVe	.77 ± .002	.75 ± .003	.75 ± .002	.77 ± .002	.68 ± .004	.83 ± .002	.84 ± .002
Node2Vec GloVe	.78 ± .002	.76 ± .003	.75 ± .002	.78 ± .002	.70 ± .005	.83 ± .002	.84 ± .002
NetMF	.79 ± .003	.76 ± .004	.80 ± .005	.79 ± .003	.67 ± .006	.88 ± .004	.89 ± .004

Supplementary Table 23: Perceptron edge prediction performance in test evaluation on Human Phenotype Ontology. The rows are sorted by balanced accuracy.

	Balanced Accuracy	F1 Score	AUPRC	Accuracy	Recall	AUROC	Precision
GLEE	.46 ± .007	.62 ± .007	.60 ± .017	.46 ± .007	.89 ± .014	.50 ± .019	.48 ± .004
NMFADMM	.50 ± .0002	.01 ± .0004	.50 ± .004	.50 ± .0002	.00 ± .0002	.50 ± .004	.46 ± .03
Node2Vec CBOW	.53 ± .014	.37 ± .157	.58 ± .077	.53 ± .014	.34 ± .292	.57 ± .077	.58 ± .041
HOPE	.56 ± .006	.24 ± .018	.81 ± .004	.56 ± .006	.13 ± .012	.77 ± .007	.94 ± .003
DeepWalk CBOW	.57 ± .018	.47 ± .147	.66 ± .069	.57 ± .018	.45 ± .298	.66 ± .059	.64 ± .064
RandNE	.60 ± .017	.40 ± .043	.70 ± .018	.60 ± .017	.27 ± .035	.69 ± .012	.79 ± .019
GraRep	.63 ± .08	.62 ± .165	.81 ± .137	.63 ± .08	.68 ± .307	.85 ± .103	.66 ± .103
Node2Vec SkipGram	.73 ± .07	.74 ± .089	.76 ± .08	.73 ± .07	.79 ± .136	.78 ± .094	.70 ± .058
DeepWalk SkipGram	.73 ± .025	.76 ± .021	.78 ± .025	.73 ± .025	.85 ± .054	.81 ± .029	.69 ± .03
First-order LINE	.74 ± .035	.66 ± .066	.89 ± .009	.74 ± .035	.51 ± .083	.86 ± .015	.95 ± .017
Second-order LINE	.76 ± .022	.77 ± .011	.84 ± .013	.76 ± .022	.80 ± .027	.84 ± .012	.75 ± .041
Walklets	.79 ± .007	.74 ± .012	.94 ± .002	.79 ± .007	.60 ± .016	.93 ± .002	.96 ± .002
NetMF	.79 ± .006	.75 ± .01	.89 ± .005	.79 ± .006	.63 ± .014	.85 ± .008	.94 ± .002
Role2Vec	.81 ± .006	.79 ± .008	.91 ± .005	.81 ± .006	.70 ± .013	.91 ± .006	.90 ± .005
Node2Vec GloVe	.82 ± .003	.83 ± .004	.87 ± .005	.82 ± .003	.85 ± .007	.89 ± .003	.80 ± .002
DeepWalk GloVe	.82 ± .003	.83 ± .003	.87 ± .006	.82 ± .003	.85 ± .006	.90 ± .003	.81 ± .003

Supplementary Table 24: Decision Tree edge prediction performance in test evaluation on Mus Musculus. The rows are sorted by balanced accuracy.

	Balanced Accuracy	F1 Score	AUPRC	Accuracy	Recall	AUROC	Precision
GLEE	.50 ± .	.00 ± .0001	1.00 ± .	.50 ± .	.00 ± .	1.00 ± .	1.00 ± .
Node2Vec GloVe	.56 ± .002	.56 ± .002	.54 ± .002	.56 ± .002	.55 ± .002	.56 ± .002	.56 ± .002
DeepWalk GloVe	.58 ± .002	.58 ± .001	.56 ± .001	.58 ± .002	.57 ± .002	.59 ± .002	.59 ± .002
DeepWalk CBOW	.61 ± .001	.60 ± .001	.57 ± .001	.61 ± .001	.59 ± .002	.60 ± .002	.61 ± .002
NMFADMM	.61 ± .003	.60 ± .004	.58 ± .002	.61 ± .003	.60 ± .004	.61 ± .003	.61 ± .003
Node2Vec CBOW	.62 ± .002	.62 ± .002	.58 ± .001	.62 ± .002	.60 ± .002	.62 ± .002	.63 ± .002
Node2Vec SkipGram	.77 ± .004	.77 ± .004	.73 ± .004	.77 ± .004	.75 ± .005	.78 ± .004	.78 ± .004
DeepWalk SkipGram	.78 ± .004	.77 ± .004	.73 ± .005	.78 ± .004	.76 ± .005	.78 ± .004	.79 ± .004
Role2Vec	.78 ± .003	.77 ± .003	.73 ± .003	.78 ± .003	.73 ± .004	.80 ± .003	.81 ± .003
Walklets	.79 ± .003	.78 ± .003	.74 ± .003	.79 ± .003	.76 ± .003	.80 ± .003	.81 ± .003
GraRep	.79 ± .002	.79 ± .002	.75 ± .004	.79 ± .002	.77 ± .003	.80 ± .003	.81 ± .002
RandNE	.80 ± .003	.79 ± .003	.75 ± .003	.80 ± .003	.79 ± .003	.80 ± .003	.80 ± .003
Second-order LINE	.81 ± .003	.81 ± .003	.76 ± .004	.81 ± .003	.79 ± .003	.82 ± .003	.83 ± .003
NetMF	.84 ± .001	.84 ± .001	.80 ± .003	.84 ± .001	.81 ± .001	.85 ± .002	.86 ± .002
First-order LINE	.84 ± .002	.84 ± .002	.80 ± .002	.84 ± .002	.80 ± .003	.86 ± .001	.87 ± .001
HOPE	.85 ± .002	.85 ± .002	.81 ± .001	.85 ± .002	.82 ± .002	.86 ± .001	.87 ± .002

Supplementary Table 25: Perceptron edge prediction performance in test evaluation on Mus Musculus. The rows are sorted by balanced accuracy.

	Balanced Accuracy	F1 Score	AUPRC	Accuracy	Recall	AUROC	Precision
GLEE	.50 ± .	.67 ± .	1.00 ± .	.50 ± .	1.00 ± .	1.00 ± .0001	.50 ± .
Node2Vec GloVe	.59 ± .002	.60 ± .002	.63 ± .002	.59 ± .002	.62 ± .004	.63 ± .003	.59 ± .002
NMFADMM	.62 ± .006	.61 ± .008	.65 ± .008	.62 ± .006	.61 ± .012	.66 ± .006	.62 ± .005
DeepWalk GloVe	.62 ± .003	.63 ± .003	.67 ± .002	.62 ± .003	.64 ± .004	.67 ± .003	.62 ± .003
DeepWalk SkipGram	.63 ± .03	.61 ± .059	.64 ± .036	.63 ± .03	.61 ± .132	.67 ± .029	.64 ± .039
Node2Vec SkipGram	.63 ± .027	.66 ± .062	.63 ± .024	.63 ± .027	.72 ± .139	.67 ± .03	.62 ± .028
HOPE	.70 ± .059	.76 ± .028	.92 ± .059	.70 ± .059	.94 ± .041	.92 ± .054	.64 ± .055
NetMF	.71 ± .092	.77 ± .05	.92 ± .077	.71 ± .092	.94 ± .061	.93 ± .063	.67 ± .112
Node2Vec CBOW	.74 ± .002	.72 ± .003	.82 ± .002	.74 ± .002	.66 ± .007	.79 ± .002	.78 ± .003
DeepWalk CBOW	.74 ± .002	.73 ± .002	.82 ± .002	.74 ± .002	.69 ± .005	.80 ± .002	.77 ± .003
GraRep	.77 ± .065	.74 ± .121	.87 ± .036	.77 ± .065	.72 ± .232	.90 ± .027	.83 ± .09
Role2Vec	.86 ± .003	.85 ± .004	.94 ± .002	.86 ± .003	.79 ± .006	.94 ± .002	.91 ± .002
RandNE	.87 ± .003	.86 ± .004	.95 ± .002	.87 ± .003	.82 ± .006	.94 ± .002	.92 ± .002
Second-order LINE	.91 ± .002	.91 ± .002	.97 ± .002	.91 ± .002	.89 ± .003	.96 ± .002	.93 ± .002
Walklets	.91 ± .002	.91 ± .002	.97 ± .001	.91 ± .002	.90 ± .004	.97 ± .001	.93 ± .002
First-order LINE	.92 ± .001	.92 ± .001	.98 ± .0005	.92 ± .001	.88 ± .002	.97 ± .0006	.96 ± .002

Supplementary Table 26: Decision Tree edge prediction performance in test evaluation on Homo Sapiens. The rows are sorted by balanced accuracy.

	Balanced Accuracy	F1 Score	AUPRC	Accuracy	Recall	AUROC	Precision
GLEE	.51 ± .0006	.02 ± .003	.99 ± .001	.51 ± .0006	.01 ± .001	1.00 ± .0006	.93 ± .011
Node2Vec GloVe	.54 ± .001	.53 ± .001	.52 ± .001	.54 ± .001	.53 ± .002	.54 ± .002	.54 ± .001
DeepWalk GloVe	.55 ± .001	.55 ± .002	.54 ± .001	.55 ± .001	.54 ± .002	.55 ± .001	.55 ± .001
NMFADMM	.59 ± .002	.58 ± .002	.56 ± .002	.59 ± .002	.58 ± .002	.59 ± .003	.59 ± .003
DeepWalk CBOW	.61 ± .002	.60 ± .002	.57 ± .002	.61 ± .002	.58 ± .003	.61 ± .002	.61 ± .002
Node2Vec CBOW	.62 ± .002	.61 ± .002	.58 ± .002	.62 ± .002	.60 ± .002	.62 ± .002	.63 ± .002
Node2Vec SkipGram	.74 ± .003	.73 ± .003	.69 ± .003	.74 ± .003	.72 ± .003	.74 ± .003	.75 ± .004
DeepWalk SkipGram	.75 ± .004	.74 ± .004	.70 ± .004	.75 ± .004	.73 ± .004	.75 ± .004	.76 ± .004
Role2Vec	.76 ± .003	.75 ± .003	.71 ± .004	.76 ± .003	.71 ± .003	.78 ± .004	.79 ± .004
GraRep	.77 ± .002	.77 ± .002	.73 ± .003	.77 ± .002	.75 ± .002	.77 ± .002	.78 ± .002
RandNE	.77 ± .002	.77 ± .002	.73 ± .002	.77 ± .002	.77 ± .002	.78 ± .002	.78 ± .002
Walklets	.78 ± .002	.77 ± .003	.73 ± .003	.78 ± .002	.74 ± .003	.79 ± .002	.80 ± .003
Second-order LINE	.78 ± .004	.78 ± .004	.74 ± .005	.78 ± .004	.75 ± .005	.79 ± .004	.80 ± .004
NetMF	.82 ± .001	.81 ± .001	.77 ± .002	.82 ± .001	.79 ± .002	.82 ± .002	.83 ± .002
First-order LINE	.82 ± .003	.81 ± .003	.77 ± .002	.82 ± .003	.77 ± .004	.83 ± .002	.85 ± .002
HOPE	.82 ± .002	.82 ± .002	.78 ± .002	.82 ± .002	.80 ± .002	.83 ± .002	.84 ± .002

Supplementary Table 27: Perceptron edge prediction performance in test evaluation on Homo Sapiens. The rows are sorted by balanced accuracy.

	Balanced Accuracy	F1 Score	AUPRC	Accuracy	Recall	AUROC	Precision
GLEE	.50 ± .	.67 ± .	.99 ± .011	.50 ± .	1.00 ± .	.99 ± .011	.50 ± .
Node2Vec GloVe	.57 ± .002	.56 ± .003	.60 ± .002	.57 ± .002	.55 ± .004	.60 ± .002	.57 ± .002
DeepWalk GloVe	.59 ± .002	.58 ± .002	.63 ± .003	.59 ± .002	.57 ± .003	.63 ± .003	.59 ± .002
NMFADMM	.62 ± .003	.61 ± .004	.64 ± .004	.62 ± .003	.60 ± .005	.65 ± .003	.62 ± .003
DeepWalk SkipGram	.63 ± .027	.63 ± .06	.64 ± .032	.63 ± .027	.64 ± .128	.67 ± .031	.63 ± .031
Node2Vec SkipGram	.64 ± .027	.65 ± .058	.65 ± .033	.64 ± .027	.70 ± .134	.68 ± .031	.63 ± .034
HOPE	.71 ± .069	.76 ± .035	.92 ± .05	.71 ± .069	.94 ± .036	.92 ± .048	.65 ± .06
Node2Vec CBOW	.74 ± .002	.72 ± .002	.82 ± .002	.74 ± .002	.66 ± .005	.79 ± .001	.79 ± .004
DeepWalk CBOW	.74 ± .002	.73 ± .003	.82 ± .002	.74 ± .002	.68 ± .006	.79 ± .002	.78 ± .004
GraRep	.77 ± .026	.75 ± .051	.86 ± .034	.77 ± .026	.73 ± .17	.89 ± .019	.83 ± .098
NetMF	.78 ± .052	.79 ± .025	.83 ± .085	.78 ± .052	.81 ± .119	.88 ± .054	.79 ± .107
Role2Vec	.85 ± .003	.83 ± .004	.93 ± .003	.85 ± .003	.77 ± .005	.93 ± .003	.92 ± .002
RandNE	.85 ± .002	.84 ± .003	.94 ± .001	.85 ± .002	.79 ± .005	.92 ± .002	.91 ± .001
Second-order LINE	.90 ± .002	.89 ± .002	.96 ± .001	.90 ± .002	.87 ± .003	.95 ± .002	.92 ± .002
Walklets	.90 ± .001	.90 ± .001	.96 ± .0009	.90 ± .001	.88 ± .002	.96 ± .001	.91 ± .002
First-order LINE	.91 ± .0007	.90 ± .0008	.97 ± .0004	.91 ± .0007	.85 ± .002	.97 ± .0005	.96 ± .002

5.2 Node-label prediction performance

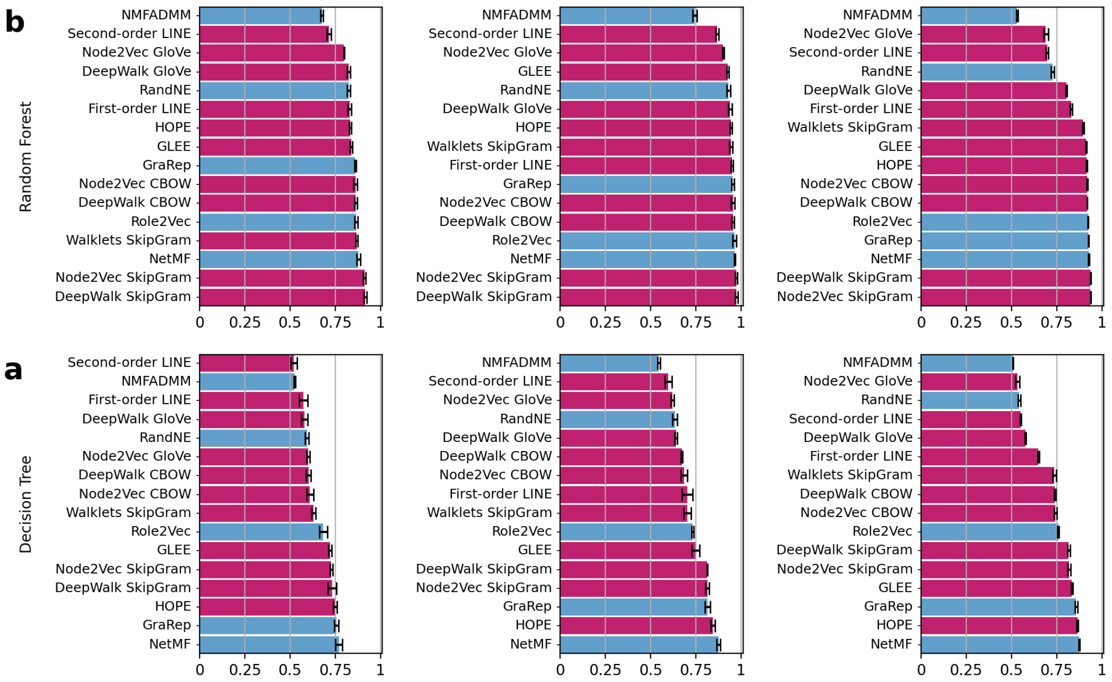

Supplementary Figure 8: **Average auroc of node label prediction models trained on embedding methods.** Results are averaged across $n = 10$ holdouts. Data are presented as mean values +/− SD. Embedding models are sorted for each task; methods implemented in *GRAPE* are in purple, while methods integrated through the *GRAPE* interface are in cyan. We show random forest and decision tree in row **a** and **b**, respectively. From left to right, CiteSeer, Cora and PubMed Diabetes datasets..

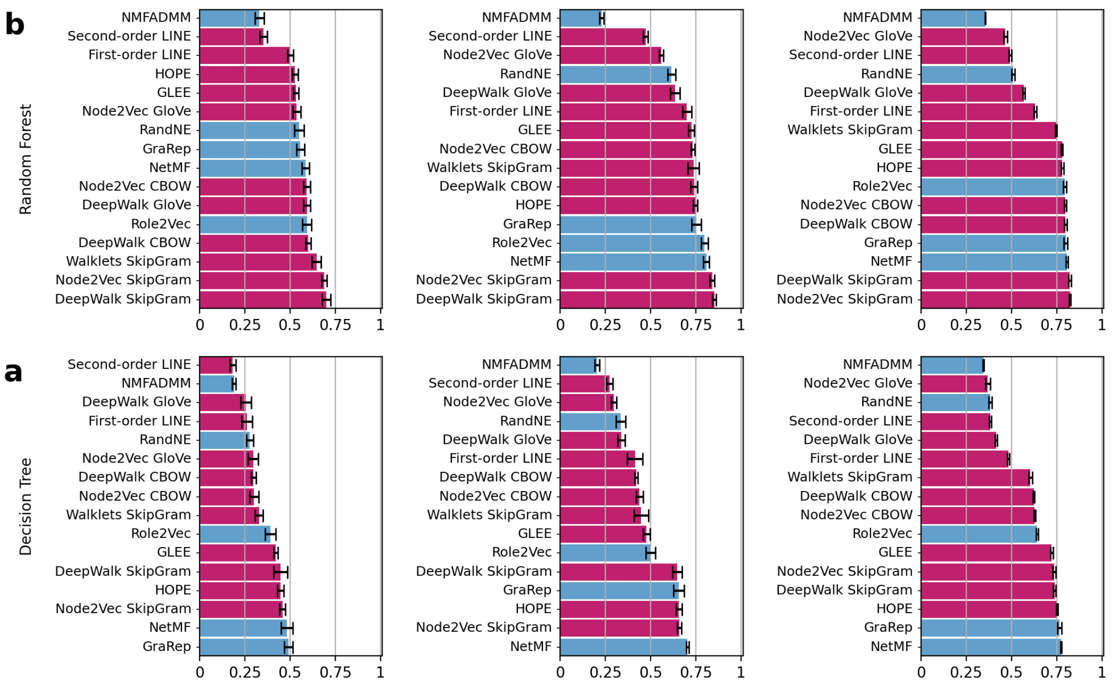

Supplementary Figure 9: **Average balanced accuracy of node label prediction models trained on embedding methods.** Results are averaged across $n = 10$ holdouts. Data are presented as mean values +/− SD. Embedding models are sorted for each task; methods implemented in *GRAPE* are in purple, while methods integrated through the *GRAPE* interface are in cyan. We show random forest and decision tree in row **a** and **b**, respectively. From left to right, CiteSeer, Cora and PubMed Diabetes datasets..

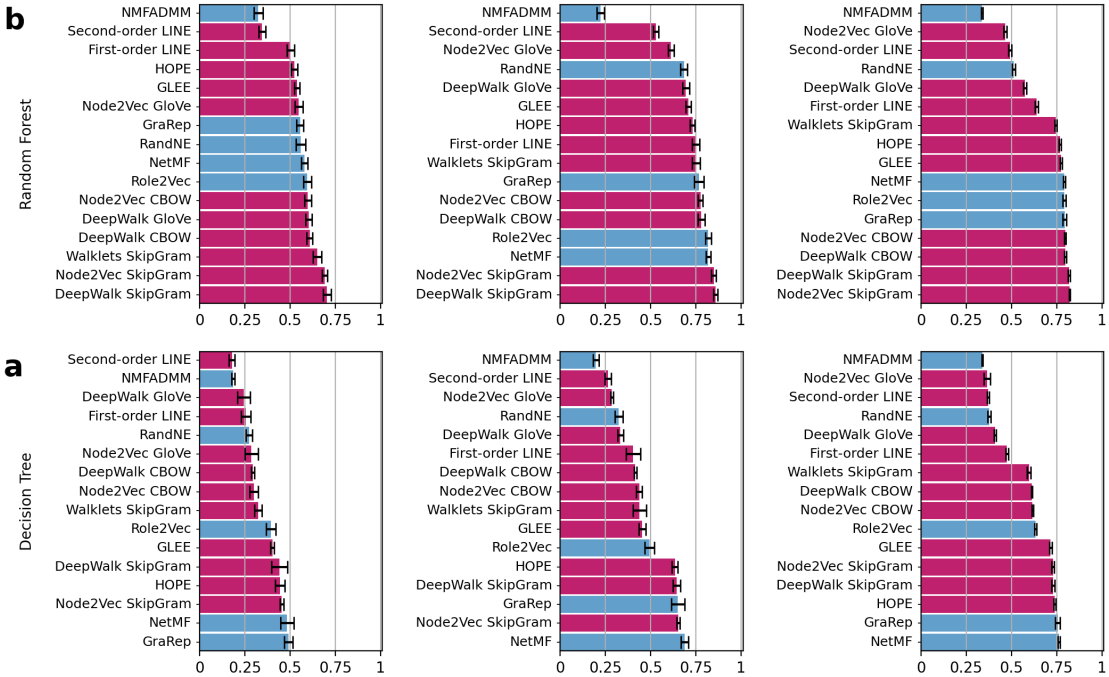

Supplementary Figure 10: **Average f1 score of node label prediction models trained on embedding methods.** Results are averaged across $n = 10$ holdouts. Data are presented as mean values $+/-$ SD. Embedding models are sorted for each task; methods implemented in *GRAPE* are in purple, while methods integrated through the *GRAPE* interface are in cyan. We show random forest and decision tree in row **a** and **b**, respectively. From left to right, CiteSeer, Cora and PubMed Diabetes datasets..

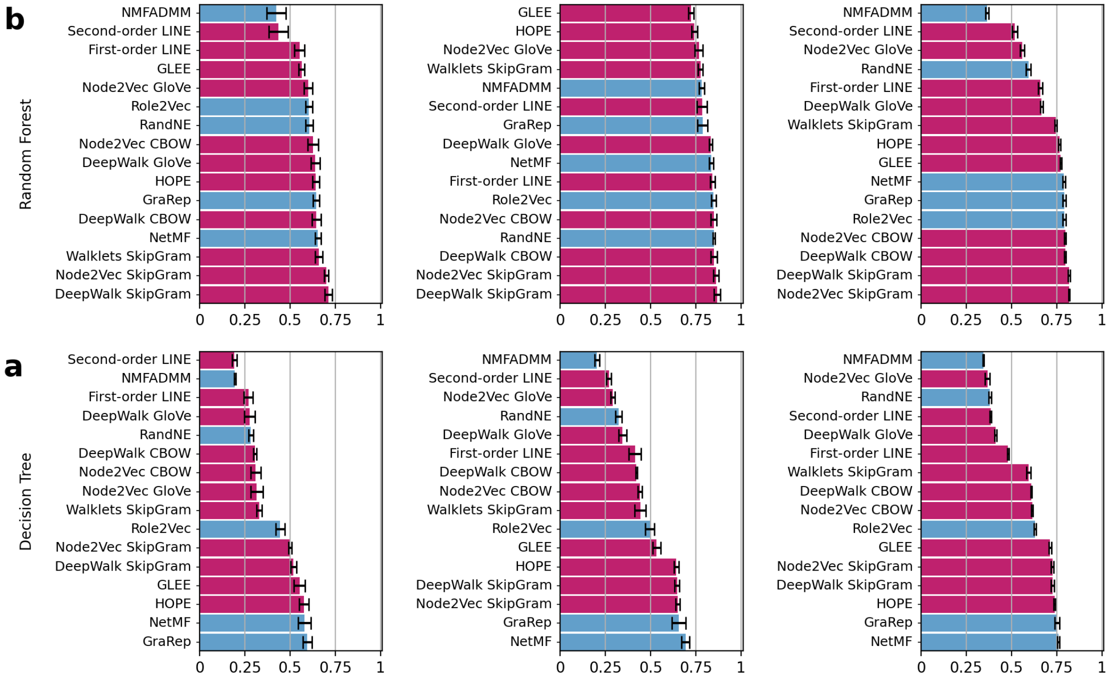

Supplementary Figure 11: **Average precision of node label prediction models trained on embedding methods.** Results are averaged across $n = 10$ holdouts. Data are presented as mean values $+/-$ SD. Embedding models are sorted for each task; methods implemented in *GRAPE* are in purple, while methods integrated through the *GRAPE* interface are in cyan. We show random forest and decision tree in row **a** and **b**, respectively. From left to right, CiteSeer, Cora and PubMed Diabetes datasets..

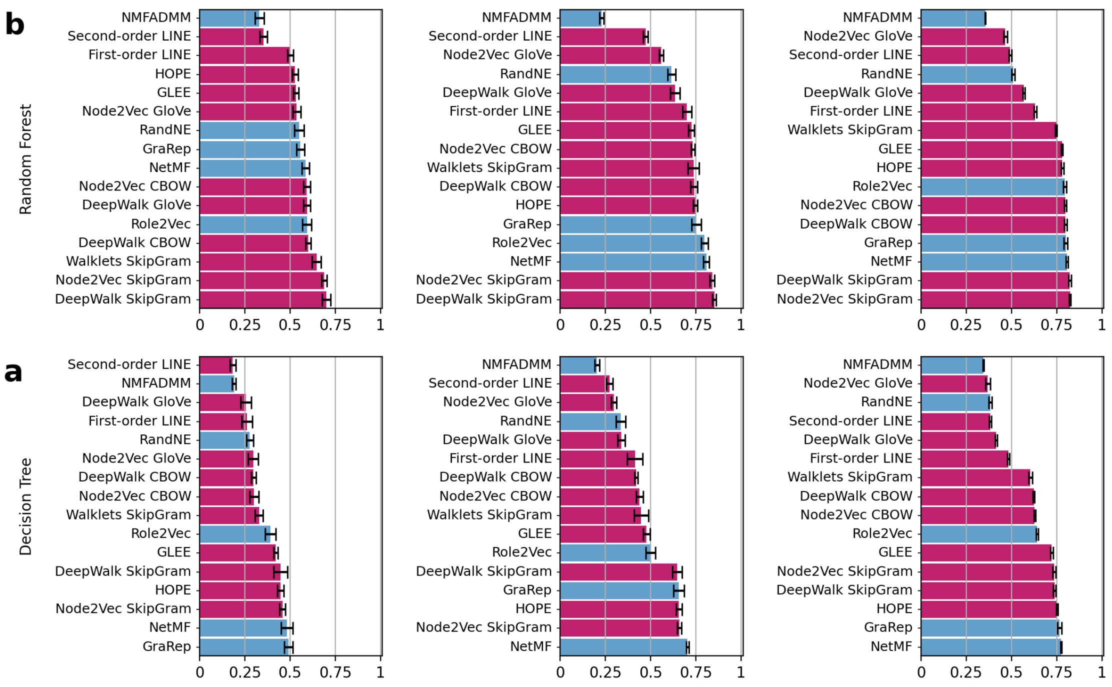

Supplementary Figure 12: **Average recall of node label prediction models trained on embedding methods.** Results are averaged across n = 10 holdouts. Data are presented as mean values +/− SD. Embedding models are sorted for each task; methods implemented in *GRAPE* are in purple, while methods integrated through the *GRAPE* interface are in cyan. We show random forest and decision tree in row **a** and **b**, respectively. From left to right, CiteSeer, Cora and PubMed Diabetes datasets..

Supplementary Table 28: Random Forest edge prediction performance in test evaluation on Cora. The rows are sorted by balanced accuracy.

	Balanced Accuracy	F1 Score	Accuracy	AUROC	Precision	Recall
NMFADMM	.23 ± .012	.22 ± .02	.37 ± .013	.74 ± .012	.78 ± .014	.23 ± .012
Second-order LINE	.47 ± .014	.53 ± .016	.57 ± .013	.87 ± .009	.78 ± .028	.47 ± .014
Node2Vec GloVe	.56 ± .013	.61 ± .016	.63 ± .009	.90 ± .004	.77 ± .022	.56 ± .013
RandNE	.62 ± .022	.68 ± .019	.68 ± .016	.93 ± .01	.85 ± .007	.62 ± .022
DeepWalk GloVe	.63 ± .026	.69 ± .019	.69 ± .017	.94 ± .01	.83 ± .009	.63 ± .026
First-order LINE	.70 ± .025	.75 ± .021	.75 ± .019	.95 ± .005	.84 ± .013	.70 ± .025
GLEE	.72 ± .016	.71 ± .015	.72 ± .015	.93 ± .006	.72 ± .014	.72 ± .016
Node2Vec CBOW	.73 ± .011	.78 ± .013	.78 ± .008	.95 ± .009	.85 ± .016	.73 ± .011
Walklets SkipGram	.74 ± .03	.75 ± .022	.76 ± .024	.94 ± .008	.78 ± .014	.74 ± .03
DeepWalk CBOW	.74 ± .02	.78 ± .019	.78 ± .013	.95 ± .007	.85 ± .019	.74 ± .02
HOPE	.75 ± .012	.73 ± .013	.75 ± .009	.94 ± .005	.74 ± .016	.75 ± .012
GraRep	.75 ± .025	.77 ± .026	.78 ± .021	.95 ± .007	.79 ± .029	.75 ± .025
Role2Vec	.80 ± .019	.82 ± .017	.83 ± .016	.96 ± .01	.85 ± .013	.80 ± .019
NetMF	.81 ± .016	.82 ± .014	.82 ± .011	.96 ± .003	.83 ± .011	.81 ± .016
Node2Vec SkipGram	.84 ± .013	.85 ± .014	.86 ± .014	.97 ± .006	.86 ± .015	.84 ± .013
DeepWalk SkipGram	.85 ± .01	.86 ± .013	.87 ± .013	.97 ± .007	.87 ± .017	.85 ± .01

Supplementary Table 29: Decision Tree edge prediction performance in test evaluation on Cora. The rows are sorted by balanced accuracy.

	Balanced Accuracy	F1 Score	Accuracy	AUROC	Precision	Recall
NMFADMM	.20 ± .013	.20 ± .016	.21 ± .031	.54 ± .007	.20 ± .015	.20 ± .013
Second-order LINE	.27 ± .018	.26 ± .017	.28 ± .017	.60 ± .019	.27 ± .013	.27 ± .018
Node2Vec GloVe	.30 ± .014	.29 ± .008	.30 ± .017	.62 ± .008	.29 ± .012	.30 ± .014
RandNE	.33 ± .026	.32 ± .022	.34 ± .022	.63 ± .013	.32 ± .017	.33 ± .026
DeepWalk GloVe	.34 ± .02	.33 ± .017	.34 ± .014	.64 ± .007	.34 ± .022	.34 ± .02
First-order LINE	.41 ± .043	.40 ± .04	.41 ± .045	.70 ± .028	.41 ± .034	.41 ± .043
DeepWalk CBOW	.42 ± .009	.42 ± .007	.42 ± .01	.67 ± .004	.42 ± .005	.42 ± .009
Node2Vec CBOW	.44 ± .019	.44 ± .017	.45 ± .016	.68 ± .018	.44 ± .013	.44 ± .019
Walklets SkipGram	.45 ± .039	.44 ± .036	.45 ± .038	.70 ± .021	.44 ± .031	.45 ± .039
GLEE	.48 ± .019	.45 ± .018	.44 ± .019	.75 ± .022	.53 ± .022	.48 ± .019
Role2Vec	.50 ± .026	.49 ± .026	.52 ± .021	.73 ± .006	.50 ± .024	.50 ± .026
DeepWalk SkipGram	.65 ± .027	.64 ± .02	.66 ± .015	.81 ± .001	.65 ± .013	.65 ± .027
GraRep	.66 ± .029	.65 ± .037	.67 ± .04	.81 ± .015	.66 ± .038	.66 ± .029
HOPE	.66 ± .017	.63 ± .016	.65 ± .019	.84 ± .011	.64 ± .012	.66 ± .017
Node2Vec SkipGram	.66 ± .01	.65 ± .009	.67 ± .005	.81 ± .011	.65 ± .013	.66 ± .01
NetMF	.70 ± .008	.69 ± .02	.69 ± .022	.87 ± .011	.69 ± .022	.70 ± .008

Supplementary Table 30: Random Forest edge prediction performance in test evaluation on CiteSeer. The rows are sorted by balanced accuracy.

	Balanced Accuracy	F1 Score	Accuracy	AUROC	Precision	Recall
NMFADMM	.33 ± .024	.33 ± .025	.38 ± .026	.68 ± .007	.42 ± .054	.33 ± .024
Second-order LINE	.35 ± .02	.35 ± .019	.40 ± .023	.72 ± .013	.44 ± .053	.35 ± .02
First-order LINE	.50 ± .016	.50 ± .02	.55 ± .013	.83 ± .011	.55 ± .028	.50 ± .016
HOPE	.53 ± .016	.52 ± .018	.51 ± .019	.83 ± .006	.64 ± .019	.53 ± .016
GLEE	.53 ± .015	.54 ± .016	.56 ± .016	.84 ± .008	.56 ± .017	.53 ± .015
Node2Vec GloVe	.54 ± .022	.55 ± .021	.57 ± .022	.80 ± .002	.60 ± .023	.54 ± .022
RandNE	.55 ± .027	.56 ± .026	.59 ± .026	.83 ± .009	.61 ± .02	.55 ± .027
GraRep	.56 ± .023	.56 ± .02	.55 ± .023	.86 ± .005	.64 ± .018	.56 ± .023
NetMF	.59 ± .02	.58 ± .018	.58 ± .021	.88 ± .01	.65 ± .016	.59 ± .02
Node2Vec CBOW	.59 ± .018	.60 ± .019	.64 ± .016	.86 ± .011	.63 ± .029	.59 ± .018
DeepWalk GloVe	.59 ± .019	.60 ± .019	.63 ± .017	.83 ± .009	.64 ± .025	.59 ± .019
Role2Vec	.59 ± .024	.60 ± .022	.62 ± .024	.87 ± .01	.60 ± .02	.59 ± .024
DeepWalk CBOW	.60 ± .015	.61 ± .016	.65 ± .015	.86 ± .008	.65 ± .025	.60 ± .015
Walklets SkipGram	.65 ± .025	.65 ± .023	.68 ± .022	.87 ± .007	.66 ± .02	.65 ± .025
Node2Vec SkipGram	.69 ± .015	.69 ± .014	.72 ± .012	.91 ± .007	.70 ± .012	.69 ± .015
DeepWalk SkipGram	.70 ± .024	.70 ± .022	.73 ± .018	.91 ± .009	.71 ± .021	.70 ± .024

Supplementary Table 31: Decision Tree edge prediction performance in test evaluation on CiteSeer. The rows are sorted by balanced accuracy.

	Balanced Accuracy	F1 Score	Accuracy	AUROC	Precision	Recall
Second-order LINE	.18 ± .015	.18 ± .015	.19 ± .013	.52 ± .017	.19 ± .013	.18 ± .015
NMFADMM	.19 ± .01	.19 ± .01	.20 ± .014	.53 ± .005	.20 ± .006	.19 ± .01
DeepWalk GloVe	.26 ± .029	.24 ± .036	.26 ± .021	.58 ± .018	.28 ± .029	.26 ± .029
First-order LINE	.26 ± .029	.26 ± .026	.27 ± .028	.57 ± .023	.27 ± .024	.26 ± .029
RandNE	.28 ± .019	.28 ± .018	.29 ± .014	.59 ± .01	.28 ± .014	.28 ± .019
Node2Vec GloVe	.30 ± .028	.29 ± .037	.30 ± .036	.60 ± .008	.32 ± .035	.30 ± .028
DeepWalk CBOW	.30 ± .012	.30 ± .009	.31 ± .003	.60 ± .013	.31 ± .01	.30 ± .012
Node2Vec CBOW	.30 ± .024	.30 ± .023	.32 ± .015	.61 ± .018	.31 ± .028	.30 ± .024
Walklets SkipGram	.33 ± .022	.33 ± .021	.34 ± .02	.63 ± .012	.33 ± .015	.33 ± .022
Role2Vec	.39 ± .03	.39 ± .027	.40 ± .034	.68 ± .023	.45 ± .025	.39 ± .03
GLEE	.42 ± .013	.40 ± .011	.39 ± .011	.72 ± .009	.55 ± .032	.42 ± .013
DeepWalk SkipGram	.45 ± .037	.44 ± .044	.44 ± .058	.73 ± .024	.52 ± .015	.45 ± .037
HOPE	.45 ± .018	.45 ± .026	.42 ± .026	.75 ± .01	.58 ± .026	.45 ± .018
Node2Vec SkipGram	.46 ± .015	.45 ± .011	.46 ± .012	.73 ± .007	.50 ± .01	.46 ± .015
NetMF	.48 ± .032	.48 ± .038	.47 ± .04	.77 ± .019	.58 ± .035	.48 ± .032
GraRep	.49 ± .023	.49 ± .024	.48 ± .029	.76 ± .012	.60 ± .025	.49 ± .023

Supplementary Table 32: Random Forest edge prediction performance in test evaluation on PubMedDiabetes. The rows are sorted by balanced accuracy.

	Balanced Accuracy	F1 Score	Accuracy	AUROC	Precision	Recall
NMFADMM	.36 ± .002	.34 ± .004	.41 ± .003	.53 ± .004	.37 ± .008	.36 ± .002
Node2Vec GloVe	.47 ± .009	.47 ± .008	.52 ± .012	.69 ± .013	.56 ± .011	.47 ± .009
Second-order LINE	.49 ± .008	.49 ± .008	.54 ± .009	.70 ± .008	.52 ± .014	.49 ± .008
RandNE	.51 ± .008	.51 ± .009	.56 ± .009	.73 ± .008	.59 ± .013	.51 ± .008
DeepWalk GloVe	.57 ± .006	.58 ± .008	.62 ± .004	.81 ± .003	.67 ± .007	.57 ± .006
First-order LINE	.63 ± .008	.64 ± .009	.67 ± .009	.83 ± .006	.66 ± .011	.63 ± .008
Walklets SkipGram	.75 ± .005	.75 ± .005	.76 ± .006	.90 ± .004	.74 ± .006	.75 ± .005
GLEE	.78 ± .005	.77 ± .005	.78 ± .004	.91 ± .004	.77 ± .004	.78 ± .005
HOPE	.78 ± .006	.77 ± .006	.78 ± .006	.92 ± .002	.77 ± .006	.78 ± .006
Role2Vec	.80 ± .009	.79 ± .008	.81 ± .007	.92 ± .002	.79 ± .009	.80 ± .009
Node2Vec CBOW	.80 ± .006	.80 ± .005	.81 ± .005	.92 ± .002	.80 ± .005	.80 ± .006
DeepWalk CBOW	.80 ± .007	.80 ± .005	.81 ± .003	.92 ± .001	.80 ± .004	.80 ± .007
GraRep	.80 ± .01	.79 ± .01	.81 ± .008	.93 ± .002	.79 ± .01	.80 ± .01
NetMF	.81 ± .007	.79 ± .006	.80 ± .006	.93 ± .002	.79 ± .006	.81 ± .007
DeepWalk SkipGram	.82 ± .008	.82 ± .006	.83 ± .005	.94 ± .001	.82 ± .006	.82 ± .008
Node2Vec SkipGram	.82 ± .004	.82 ± .003	.83 ± .002	.94 ± .001	.82 ± .003	.82 ± .004

Supplementary Table 33: Decision Tree edge prediction performance in test evaluation on PubMedDiabetes. The rows are sorted by balanced accuracy.

	Balanced Accuracy	F1 Score	Accuracy	AUROC	Precision	Recall
NMFADMM	.35 ± .004	.34 ± .003	.35 ± .011	.51 ± .001	.35 ± .004	.35 ± .004
Node2Vec GloVe	.37 ± .014	.36 ± .018	.38 ± .016	.53 ± .012	.37 ± .013	.37 ± .014
RandNE	.38 ± .009	.38 ± .008	.39 ± .007	.54 ± .007	.38 ± .008	.38 ± .009
Second-order LINE	.38 ± .005	.37 ± .006	.38 ± .008	.55 ± .002	.39 ± .004	.38 ± .005
DeepWalk GloVe	.42 ± .005	.41 ± .005	.42 ± .005	.58 ± .004	.41 ± .006	.42 ± .005
First-order LINE	.48 ± .006	.48 ± .008	.49 ± .011	.65 ± .004	.48 ± .005	.48 ± .006
Walklets SkipGram	.61 ± .011	.60 ± .011	.61 ± .012	.74 ± .011	.60 ± .011	.61 ± .011
DeepWalk CBOW	.62 ± .004	.61 ± .003	.62 ± .004	.74 ± .004	.61 ± .003	.62 ± .004
Node2Vec CBOW	.63 ± .004	.62 ± .005	.63 ± .006	.74 ± .007	.61 ± .005	.63 ± .004
Role2Vec	.64 ± .005	.63 ± .005	.65 ± .006	.76 ± .004	.63 ± .005	.64 ± .005
GLEE	.72 ± .007	.72 ± .007	.73 ± .007	.83 ± .004	.71 ± .007	.72 ± .007
Node2Vec SkipGram	.74 ± .008	.73 ± .008	.74 ± .008	.82 ± .008	.73 ± .007	.74 ± .008
DeepWalk SkipGram	.74 ± .008	.73 ± .009	.74 ± .01	.82 ± .007	.73 ± .009	.74 ± .008
HOPE	.75 ± .004	.74 ± .006	.75 ± .007	.87 ± .004	.74 ± .005	.75 ± .004
GraRep	.77 ± .011	.76 ± .013	.77 ± .014	.86 ± .008	.75 ± .013	.77 ± .011
NetMF	.78 ± .003	.76 ± .005	.77 ± .006	.87 ± .003	.76 ± .005	.78 ± .003

6 Large real-world graph experimental results and big graphs used in the experiments.

This Note includes detailed information about the data and the software we used to build up the three real world big graphs used in the experiments and more information about the state-of-the-art graph libraries we used to compare *GRAPE* performance, as well as additional results about large real-world graph experiments.

6.1 Big graphs description and construction

English Wikipedia. Wikipedia graphs are web graphs with nodes representing either Wiki sites pages or related websites; edges represent the links between the pages. In the experiments, we used the English Wikipedia graph having 17 million nodes and 130 million (undirected) edges (2021-11-01 version). The task for the English Wikipedia graph is a whole-graph edge prediction, that is, predicting whether an edge connects two given nodes in the entire graph. The set of positive edges is defined as the undirected edges present in the entire graph (about 130 million). The set of negative edges is instead defined as the edges that are not present in the graph, that is, around 150 trillion undirected edges.

Comparative Toxicogenomic Database (CTD). CTD is a publicly available database that aims to advance the understanding of how environmental exposures affect human health. It provides manually curated information about chemical–gene/protein interactions, chemical–disease, and gene-disease relationships. Also, it includes information about phenotypes, pathways, ontologies, and their relations with genes, chemicals, and diseases, including about 45 million edges and more than $100K$ nodes. The CTD edge-prediction task consists in predicting *gene-disease* associations. The set of positive edges is defined as the set of all the existing (undirected) relationships (edges) between gene and disease nodes, which amounts to about 29 million. Negative edges were defined by pairs of gene-disease being unrelated in the CTD dataset (about 362 million "negative" edges).

PheKnowLator biomedical data. PheKnowLator is a software resource designed to facilitate the construction of large-scale biomedical knowledge graphs using several knowledge models (instance-based and subclass-based). PheKnowLator currently integrates 12 Open Biomedical Ontologies and 31 linked open-data sources. In our experiments, we used a 2022-04-11 build including about 7 millions of (undirected) edges and about $800K$ nodes. The PheKnowLator task consists in the prediction of *genetic variant-disease* associations, using $44K$ known "positive" associations and a set of "negative" edges, including about 3 billions of variant-disease pairs having no known associations.

Supplementary Table 34 reports the pre-processed data we used to construct the graphs. Supplementary Tables 35 and 36 show data and scripts used to build up respectively the PheKnowLator Knowledge Graph and the CTD and Wikipedia graphs.

Resource	Link
Prebuilt CTD	https://archive.org/download/ctd_20220404/CTD.tar
Prebuilt PheKnowLator	https://archive.org/download/pheknowlator_20220411/PheKnowLator.tar
Prebuilt English Wikipedia	https://archive.org/download/wikipedia_edge_list.npy/wikipedia_edge_list.npy.gz

Supplementary Table 34: Preprocessed datasets used to build-up the experiments for CTD, the PheKnowLator biomedical KG and Wikipedia.

Resource	Link
PheKnowLator build datasets	https://github.com/callahantiff/PheKnowLator/wiki/v2-Data-Sources
PheKnowLator used in experiments	https://storage.googleapis.com/pheknowlator/archived_builds/release_v3.0.2/build_18OCT2021/data/original_data/downloaded_build_metadata.txt
Preprocessed PheKnowLator data	https://storage.googleapis.com/pheknowlator/archived_builds/release_v3.0.2/build_18OCT2021/data/processed_data/preprocessed_build_metadata.txt
PheKnowLator build scripts	https://github.com/callahantiff/PheKnowLator/tree/master/builds
PheKnowLator build logs	https://storage.googleapis.com/pheknowlator/archived_builds/release_v3.0.2/build_18OCT2021/knowledge_graphs/subclass_builds/inverse_relations/owlnets/pkt_build_log.log

Supplementary Table 35: Ontologies, open link data sources and scripts used for the generation of the PheKnowLator Knowledge Graph.

Resource	Link
Complete CTD dataset	http://ctdbase.org/reports/
Dumps of Wikipedia	https://dumps.wikimedia.org/backup-index.html
English Wikipedia script	https://github.com/AnacletoLAB/ensmallen/blob/develop/bindings/python/ensmallen/datasets/wikipedia_automatic_graph_retrieval.py

Supplementary Table 36: Data and script used to construct the CTD and English Wikipedia graphs.

6.2 State of the art graph libraries compared with *GRAPE*

In the experiments, we used two *GRAPE* implementations of embedding algorithms: CBOW and SkipGram. We compared them with the following state-of-the-art embedding libraries, widely used by the scientific community:

- *PecanPy* [9] is a Python library implementing a Numba-based version of *node2vec*, leveraging Numba's just-in-time Python compilation [2] to generate the RWs on the input graph, and forwarding them to an embedding model provided by Gensim natural language processing library [29].
- *NodeVectors*[1] is a Python package that enables fast and scalable node embedding algorithms. It leverages CSR matrix storage for graphs, but it also support NetworkX [3] graph loading. Besides *node2vec*, the library also implements several kinds of first and second-order RWs.
- *SNAP* [30], Stanford Network Analysis Platform, is a general-purpose system for manipulating and analysing large networks written in C++. Once compiled, it becomes an executable to analyze and compute different statistics about the graphs; it also implements different kinds of graph-processing algorithms and allows computing node embeddings by using a pre-processing phase for pre-computation of transition probabilities through the Alias method [5].
- *Node2Vec*[2] is a Python package for embedding networks through RW-based algorithms like *node2vec*. Similar to SNAP, it employs the Alias method [5] to pre-compute transition probabilities. It also handles the graph loading through the NetworkX library [3].
- *GraphEmbedding*[3] is a Python package that handles network embeddings with RW-based methods. Again, the transition probability is pre-computed via the Alias method and employs the NetworkX library to handle the loading of a graph.
- *FastNode2Vec*[4] implements the *node2vec* algorithm, leveraging both Numba and Gensim. This implementation scales linearly, in time and memory, with respect to the dimension of the input graph.

[1] https://github.com/VHRanger/nodevectors
[2] https://github.com/eliorc/node2vec
[3] https://github.com/shenweichen/GraphEmbedding
[4] https://github.com/louisabraham/fastnode2vec

- *PyTorch Geometric* [5] is a library built upon PyTorch mainly designed to develop and apply GNN for a wide range of applications related to structured data. It also provides a *node2vec* implementation using negative sampling optimization.

6.3 Evaluation of the results

For all of the considered tasks, we firstly computed the embedded graphs using graph libraries and then the resulting embeddings have been used to train machine learning methods for an edge prediction problem. To evaluate the ML models we adopted a connected Monte Carlo (Supplementary Information S9.2) repeated ten times, with a train:test ratio equal to 80% : 20% of the data. As evaluation metrics we applied precision, recall, accuracy, balanced accuracy, F1, AUROC, and AUPRC. In the experimental set-up we imposed the following memory and time constraints, using a Google Cloud VM with 64 cores[6]:

- A maximum time of 48 hours for each holdout to produce the embedding;
- The maximum memory usage allowed during the embedding phase is 64GB.
- The maximum memory usage allowed during the prediction phase is 256GB.

To keep track of memory and time requirements and of possible stops for exceptions and system-related errors (out of memory, core dumps), the Python library `memory_time_tracker` was used[7].

6.4 Additional experimental results with big graphs

Supplementary Figures and Supplementary Tables in this section show the comparison of the experimental results on the above big real-world graphs estimated using different metrics obtained by the Decision Trees trained on Node2vec embeddings generated by *GRAPE* and the other state-of-the-art graph embedding libraries.

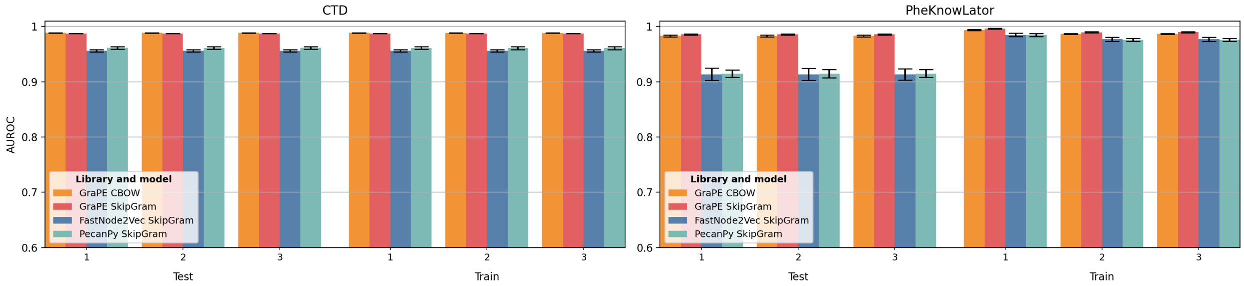

Supplementary Figure 13: **Average auroc of Decision Tree trained.** Results are averaged across ten holdouts. Data are presented as mean values $+/-$ SD. The bar plot on the left is relative to the CTD graph, while the one on the right is relative to the PheKnowLator graph. The number on the horizontal axis represents different unbalance rates of existing and non-existing edges considered during the evaluation: 1 means a balanced rate of existing and non-existing edges, 2 means 1 : 2 and 3 means 1 : 3. All embedding methods achieve worse performance with the increasing unbalance rates, but *GRAPE* implementations consistently outperform the other GenSim-based libraries.

[5]https://github.com/pyg-team/pytorch_geometric
[6]N1 Cpus with Intel Haswell micro-architecture
[7]https://github.com/LucaCappelletti94/memory_time_tracker

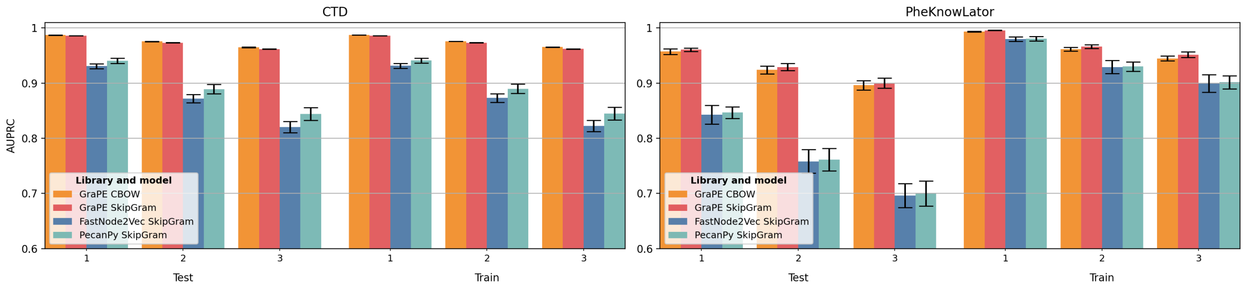

Supplementary Figure 14: **Average auprc of Decision Tree trained.** Results are averaged across ten holdouts. Data are presented as mean values +/− SD. The bar plot on the left is relative to the CTD graph, while the one on the right is relative to the PheKnowLator graph. The number on the horizontal axis represents different unbalance rates of existing and non-existing edges considered during the evaluation: 1 means a balanced rate of existing and non-existing edges, 2 means 1 : 2 and 3 means 1 : 3. All embedding methods achieve worse performance with the increasing unbalance rates, but *GRAPE* implementations consistently outperform the other GenSim-based libraries.

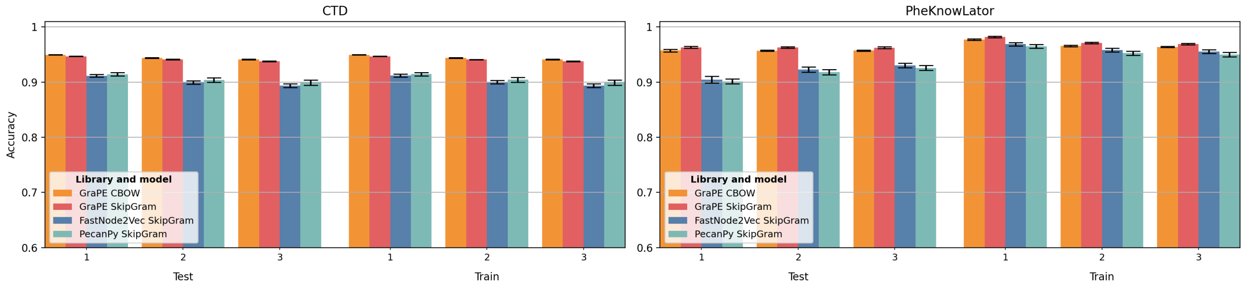

Supplementary Figure 15: **Average accuracy of Decision Tree trained.** Results are averaged across ten holdouts. Data are presented as mean values +/− SD. The bar plot on the left is relative to the CTD graph, while the one on the right is relative to the PheKnowLator graph. The number on the horizontal axis represents different unbalance rates of existing and non-existing edges considered during the evaluation: 1 means a balanced rate of existing and non-existing edges, 2 means 1 : 2 and 3 means 1 : 3. All embedding methods achieve worse performance with the increasing unbalance rates, but *GRAPE* implementations consistently outperform the other GenSim-based libraries.

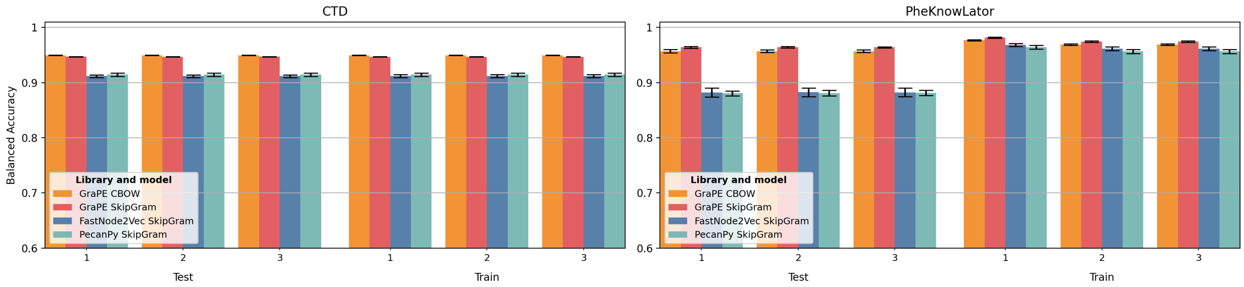

Supplementary Figure 16: **Average balanced accuracy of Decision Tree trained.** Results are averaged across ten holdouts. Data are presented as mean values +/− SD. The bar plot on the left is relative to the CTD graph, while the one on the right is relative to the PheKnowLator graph. The number on the horizontal axis represents different unbalance rates of existing and non-existing edges considered during the evaluation: 1 means a balanced rate of existing and non-existing edges, 2 means 1 : 2 and 3 means 1 : 3. All embedding methods achieve worse performance with the increasing unbalance rates, but *GRAPE* implementations consistently outperform the other GenSim-based libraries.

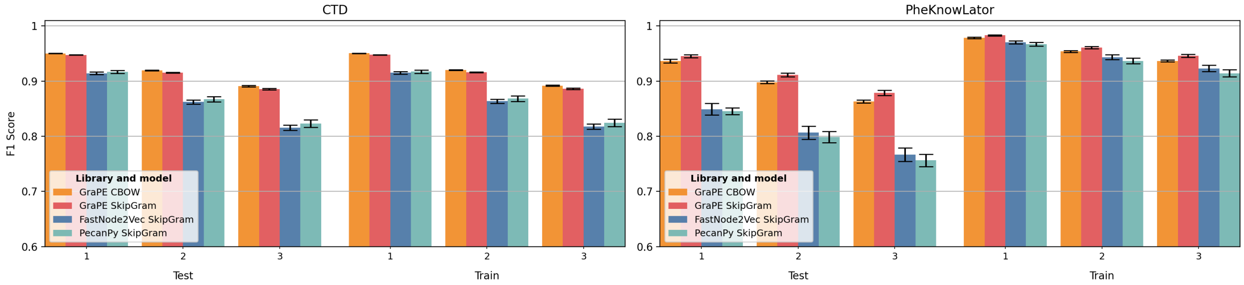

Supplementary Figure 17: **Average f1 score of Decision Tree trained.** Results are averaged across ten holdouts. Data are presented as mean values +/− SD. The bar plot on the left is relative to the CTD graph, while the one on the right is relative to the PheKnowLator graph. The number on the horizontal axis represents different unbalance rates of existing and non-existing edges considered during the evaluation: 1 means a balanced rate of existing and non-existing edges, 2 means 1 : 2 and 3 means 1 : 3. All embedding methods achieve worse performance with the increasing unbalance rates, but *GRAPE* implementations consistently outperform the other GenSim-based libraries.

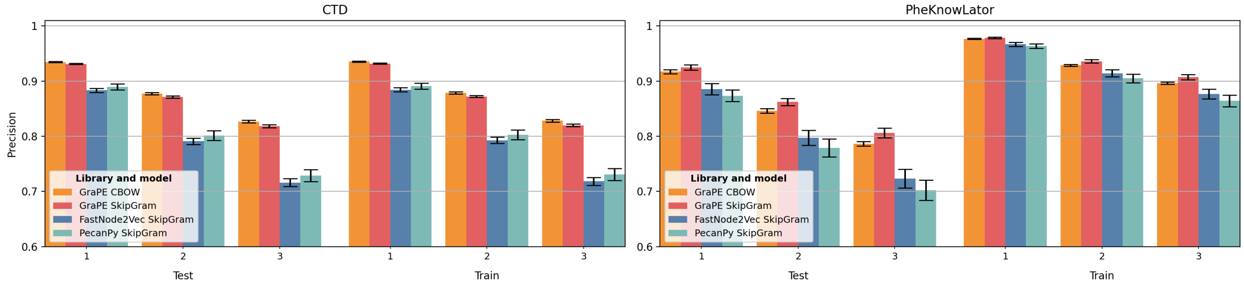

Supplementary Figure 18: **Average precision of Decision Tree trained.** Results are averaged across ten holdouts. Data are presented as mean values +/− SD. The bar plot on the left is relative to the CTD graph, while the one on the right is relative to the PheKnowLator graph. The number on the horizontal axis represents different unbalance rates of existing and non-existing edges considered during the evaluation: 1 means a balanced rate of existing and non-existing edges, 2 means 1 : 2 and 3 means 1 : 3. All embedding methods achieve worse performance with the increasing unbalance rates, but *GRAPE* implementations consistently outperform the other GenSim-based libraries.

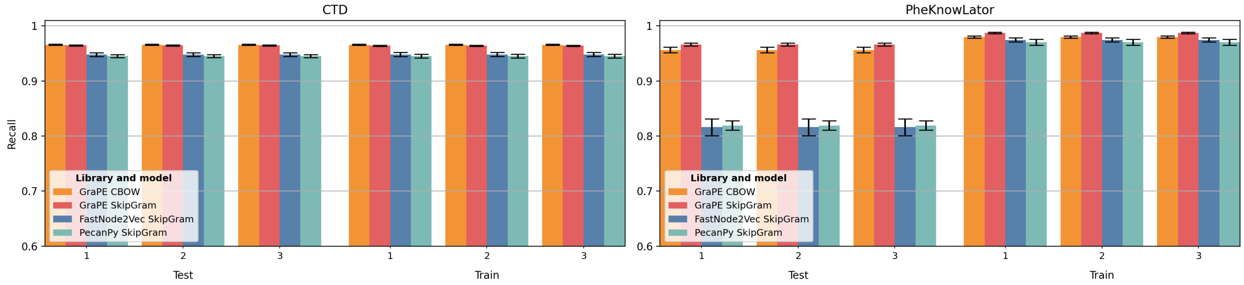

Supplementary Figure 19: **Average recall of Decision Tree trained.** Results are averaged across ten holdouts. Data are presented as mean values +/− SD. The bar plot on the left is relative to the CTD graph, while the one on the right is relative to the PheKnowLator graph. The number on the horizontal axis represents different unbalance rates of existing and non-existing edges considered during the evaluation: 1 means a balanced rate of existing and non-existing edges, 2 means 1 : 2 and 3 means 1 : 3. All embedding methods achieve worse performance with the increasing unbalance rates, but *GRAPE* implementations consistently outperform the other GenSim-based libraries.

Supplementary Table 37: Decision Tree edge prediction performance in train evaluation on CTD with unbalance rate 1

library and model	Accuracy	Precision	F1 Score	AUPRC	Balanced Accuracy	Recall	AUROC
FastNode2Vec SkipGram	.91 ± .0027	.88 ± .0036	.92 ± .0025	.93 ± .0042	.91 ± .0027	.95 ± .0036	.96 ± .002
GRAPE CBOW	.95 ± .0003	.94 ± .0011	.95 ± .0003	.99 ± .0002	.95 ± .0003	.97 ± .0011	.99 ± .0001
GRAPE SkipGram	.95 ± .0003	.93 ± .0011	.95 ± .0003	.99 ± .0001	.95 ± .0003	.96 ± .001	.99 ± .0001
PecanPy SkipGram	.91 ± .0031	.89 ± .0054	.92 ± .0028	.94 ± .0047	.91 ± .0031	.95 ± .0031	.96 ± .0027

Supplementary Table 38: Decision Tree edge prediction performance in train evaluation on CTD with unbalance rate 2

library and model	Accuracy	Precision	F1 Score	AUPRC	Balanced Accuracy	Recall	AUROC
FastNode2Vec SkipGram	.90 ± .003	.79 ± .0058	.86 ± .0037	.87 ± .0075	.91 ± .0027	.95 ± .0036	.96 ± .002
GRAPE CBOW	.94 ± .0006	.88 ± .0019	.92 ± .0007	.98 ± .0003	.95 ± .0003	.97 ± .0011	.99 ± .0001
GRAPE SkipGram	.94 ± .0006	.87 ± .0019	.92 ± .0007	.97 ± .0001	.95 ± .0003	.96 ± .001	.99 ± .0001
PecanPy SkipGram	.90 ± .0041	.80 ± .0087	.87 ± .0049	.89 ± .0084	.91 ± .0031	.95 ± .0031	.96 ± .0027

Supplementary Table 39: Decision Tree edge prediction performance in train evaluation on CTD with unbalance rate 3

library and model	Accuracy	Precision	F1 Score	AUPRC	Balanced Accuracy	Recall	AUROC
FastNode2Vec SkipGram	.89 ± .0033	.72 ± .0072	.82 ± .0048	.82 ± .0102	.91 ± .0027	.95 ± .0036	.96 ± .002
GRAPE CBOW	.94 ± .0007	.83 ± .0026	.89 ± .0012	.97 ± .0004	.95 ± .0003	.97 ± .0011	.99 ± .0001
GRAPE SkipGram	.94 ± .0007	.82 ± .0025	.89 ± .0011	.96 ± .0002	.95 ± .0003	.96 ± .001	.99 ± .0001
PecanPy SkipGram	.90 ± .0047	.73 ± .0109	.82 ± .0067	.84 ± .0114	.91 ± .0031	.95 ± .0031	.96 ± .0027

Supplementary Table 40: Decision Tree edge prediction performance in test evaluation on CTD with unbalance rate 1

library and model	Accuracy	Precision	F1 Score	AUPRC	Balanced Accuracy	Recall	AUROC
FastNode2Vec SkipGram	.91 ± .0026	.88 ± .0036	.91 ± .0024	.93 ± .0043	.91 ± .0026	.95 ± .0035	.96 ± .002
GRAPE CBOW	.95 ± .0004	.93 ± .0011	.95 ± .0003	.99 ± .0002	.95 ± .0004	.97 ± .0011	.99 ± .0001
GRAPE SkipGram	.95 ± .0003	.93 ± .0011	.95 ± .0003	.99 ± .0001	.95 ± .0003	.96 ± .001	.99 ± .0001
PecanPy SkipGram	.91 ± .0031	.89 ± .0054	.92 ± .0028	.94 ± .0047	.91 ± .0031	.95 ± .003	.96 ± .0026

Supplementary Table 41: Decision Tree edge prediction performance in test evaluation on CTD with unbalance rate 2

library and model	Accuracy	Precision	F1 Score	AUPRC	Balanced Accuracy	Recall	AUROC
FastNode2Vec SkipGram	.90 ± .003	.79 ± .0058	.86 ± .0037	.87 ± .0076	.91 ± .0026	.95 ± .0035	.96 ± .002
GRAPE CBOW	.94 ± .0006	.88 ± .002	.92 ± .0007	.98 ± .0003	.95 ± .0004	.97 ± .0011	.99 ± .0001
GRAPE SkipGram	.94 ± .0006	.87 ± .0019	.92 ± .0007	.97 ± .0001	.95 ± .0003	.96 ± .001	.99 ± .
PecanPy SkipGram	.90 ± .0041	.80 ± .0089	.87 ± .005	.89 ± .0085	.91 ± .0031	.95 ± .003	.96 ± .0027

Supplementary Table 42: Decision Tree edge prediction performance in test evaluation on CTD with unbalance rate 3

library and model	Accuracy	Precision	F1 Score	AUPRC	Balanced Accuracy	Recall	AUROC
FastNode2Vec SkipGram	.89 ± .0033	.72 ± .0072	.82 ± .0047	.82 ± .0102	.91 ± .0026	.95 ± .0035	.96 ± .002
GRAPE CBOW	.94 ± .0007	.83 ± .0025	.89 ± .0011	.96 ± .0004	.95 ± .0003	.97 ± .0011	.99 ± .0001
GRAPE SkipGram	.94 ± .0007	.82 ± .0025	.89 ± .0011	.96 ± .0002	.95 ± .0003	.96 ± .001	.99 ± .0001
PecanPy SkipGram	.90 ± .0047	.73 ± .0109	.82 ± .0067	.84 ± .0115	.91 ± .0031	.95 ± .003	.96 ± .0026

Supplementary Table 43: Decision Tree edge prediction performance in train evaluation on PheKnowLator with unbalance rate 1

library and model	Accuracy	Precision	F1 Score	AUPRC	Balanced Accuracy	Recall	AUROC
FastNode2Vec SkipGram	.97 ± .0029	.97 ± .0036	.97 ± .0027	.98 ± .0043	.97 ± .0029	.97 ± .0037	.98 ± .003
GRAPE CBOW	.98 ± .0013	.98 ± .0009	.98 ± .0012	.99 ± .0008	.98 ± .0012	.98 ± .002	.99 ± .0009
GRAPE SkipGram	.98 ± .0012	.98 ± .0014	.98 ± .0011	10 ± .0005	.98 ± .0012	.99 ± .0015	10 ± .0005
PecanPy SkipGram	.96 ± .0035	.96 ± .0038	.97 ± .0033	.98 ± .004	.96 ± .0034	.97 ± .0057	.98 ± .0025

Supplementary Table 44: Decision Tree edge prediction performance in train evaluation on PheKnowLator with unbalance rate 2

library and model	Accuracy	Precision	F1 Score	AUPRC	Balanced Accuracy	Recall	AUROC
FastNode2Vec SkipGram	.96 ± .0033	.91 ± .0065	.94 ± .0043	.93 ± .0116	.96 ± .0032	.97 ± .0037	.98 ± .0037
GRAPE CBOW	.97 ± .0012	.93 ± .0016	.95 ± .0016	.96 ± .0033	.97 ± .0013	.98 ± .002	.99 ± .0008
GRAPE SkipGram	.97 ± .0014	.94 ± .0032	.96 ± .0019	.97 ± .0036	.97 ± .0013	.99 ± .0015	.99 ± .0012
PecanPy SkipGram	.95 ± .0038	.91 ± .0078	.94 ± .0049	.93 ± .0085	.96 ± .0037	.97 ± .0057	.98 ± .0028

Supplementary Table 45: Decision Tree edge prediction performance in train evaluation on PheKnowLator with unbalance rate 3

library and model	Accuracy	Precision	F1 Score	AUPRC	Balanced Accuracy	Recall	AUROC
FastNode2Vec SkipGram	.96 ± .0035	.88 ± .009	.92 ± .0057	.90 ± .0159	.96 ± .0032	.97 ± .0037	.98 ± .0036
GRAPE CBOW	.96 ± .001	.90 ± .002	.94 ± .0018	.94 ± .0045	.97 ± .0013	.98 ± .002	.99 ± .0008
GRAPE SkipGram	.97 ± .0016	.91 ± .0048	.95 ± .0027	.95 ± .0051	.97 ± .0014	.99 ± .0015	.99 ± .0012
PecanPy SkipGram	.95 ± .004	.86 ± .0104	.91 ± .0064	.90 ± .0118	.96 ± .0037	.97 ± .0057	.98 ± .0029

Supplementary Table 46: Decision Tree edge prediction performance in test evaluation on PheKnowLator with unbalance rate 1

library and model	Accuracy	Precision	F1 Score	AUPRC	Balanced Accuracy	Recall	AUROC
FastNode2Vec SkipGram	.90 ± .0062	.89 ± .0101	.85 ± .0105	.84 ± .0167	.88 ± .0082	.82 ± .0152	.91 ± .0112
GRAPE CBOW	.96 ± .0024	.92 ± .0037	.94 ± .0036	.96 ± .0053	.96 ± .003	.96 ± .0053	.98 ± .0017
GRAPE SkipGram	.96 ± .0018	.92 ± .0047	.95 ± .0026	.96 ± .0032	.96 ± .0017	.97 ± .0027	.99 ± .0011
PecanPy SkipGram	.90 ± .0042	.87 ± .0104	.85 ± .0063	.85 ± .0105	.88 ± .0046	.82 ± .0086	.91 ± .0065

Supplementary Table 47: Decision Tree edge prediction performance in test evaluation on PheKnowLator with unbalance rate 2

library and model	Accuracy	Precision	F1 Score	AUPRC	Balanced Accuracy	Recall	AUROC
FastNode2Vec SkipGram	.92 ± .0046	.80 ± .0134	.81 ± .0117	.76 ± .0215	.88 ± .0081	.82 ± .0152	.91 ± .0108
GRAPE CBOW	.96 ± .0011	.85 ± .004	.90 ± .0026	.92 ± .0072	.96 ± .0024	.96 ± .0053	.98 ± .0016
GRAPE SkipGram	.96 ± .0015	.86 ± .0066	.91 ± .0033	.93 ± .0063	.96 ± .0012	.97 ± .0027	.99 ± .0012
PecanPy SkipGram	.92 ± .0048	.78 ± .0161	.80 ± .0101	.76 ± .0201	.88 ± .0053	.82 ± .0086	.91 ± .0073

Supplementary Table 48: Decision Tree edge prediction performance in test evaluation on PheKnowLator with unbalance rate 3

library and model	Accuracy	Precision	F1 Score	AUPRC	Balanced Accuracy	Recall	AUROC
FastNode2Vec SkipGram	.93 ± .004	.72 ± .0169	.77 ± .0121	.70 ± .0218	.88 ± .0076	.82 ± .0152	.91 ± .0102
GRAPE CBOW	.96 ± .001	.79 ± .0042	.86 ± .0029	.90 ± .0085	.96 ± .0025	.96 ± .0053	.98 ± .0017
GRAPE SkipGram	.96 ± .0017	.81 ± .0088	.88 ± .0046	.90 ± .0092	.96 ± .001	.97 ± .0027	.99 ± .0013
PecanPy SkipGram	.93 ± .0044	.70 ± .0184	.76 ± .0113	.70 ± .0225	.88 ± .0047	.82 ± .0086	.92 ± .0069

7 Ensmallen

7.1 Elias-Fano scheme

Ensmallen allows fast graph loading and processing of graph queries by using an optimized graph representation. In particular, after assigning a numeric identifier to each node of the graph, the adjacency matrix is transformed into a sorted list of integers by a bijective mapping that exploits the numeric representation of the nodes (see Supplementary subsection 7.1.1), which is then represented in memory by using Elias-Fano scheme [31, 32]. Such representation allows storing n non-negative integers, sorted in increasing orders and bounded by u, with at most $\mathcal{EF}(n,u) = 2n + n\left\lceil \log_2 \frac{u}{n} \right\rceil$ bits, and a memory usage that is less than half a bit away [33, 31, 34] from the succinct bound that is $Z + o(Z)$, where Z is the theoretical minimum number of bits needed to store the data: $Z = \left\lceil \log_2 \binom{u}{n} \right\rceil = n \log_2 \frac{u}{n} + \mathcal{O}(n)$ [35]. We support the description of Elias-Fano schema with an example, depicted in Supplementary Figure 21 of Elias-Fano [31] quasi-succint representation of the monotone list of integers [5, 8, 8, 15, 32]. In particular, the binary representation of the i-th value of the list, b_i, $i = 1, \ldots, n$, is initially split into two parts: a low-bits part, l_i, containing the lower $\lfloor \log_2 \frac{u}{n} \rfloor$ bits, and a high-bits part, h_i, containing the remaining bits (referred to as high-bits). Then, a low-bits array, (light-blue and referred to as L in Supplementary Figure 21) is formed by sequentially concatenating the explicit copies of the low-bits parts, while a high-bits array (red, and named H in Supplementary Figure 21) is composed by sequentially concatenating the gaps (differences) between consecutive high-bits parts (where the preceding value of the first element is assumed to be equal to zero), where each gap is represented by using the inverted unary representation, where an integer value equal to k is encoded by using k zeros followed by a one (for example, 0 in inverted unary representation is 1, 3 is 0001). Instead of computing the gaps, Pibiri et al. [36] propose a faster method to build an high bits array H which is equal to that obtained by Elias Fano. Specifically, Pibiri's et al. show that H can be created as a bit-vector with all zeros, exept for the elements at indexes $h_i + i$ (where i indexes the high-bits parts, h_i), which are set to 1. This method is faster because the encoding and decoding of each value no longer depends on the previous values (as gaps would) so that the representation may be built in parallel to further speed up the graph representation. Moreover, once the index of each value is known, exploiting atomic integers we can **build Elias-Fano fully in parallel** [8] without any lock. An example of this method is in Supplementary Figure 23, where the high bits index is no-longer computed using the gaps. The two fundamental operations to perform on Elias-Fano are Jacobson's **rank** and **select**. In particular, given a set of integers S, Jacobson defined the **rank** and **select** operations as follows [37]:

**select**(S, i)	returns the i-th smallest value in S
**rank**(S, m)	returns the number of elements in S less or equal than m

To speed up computation, we deviate from this definition by implementing a rank operation that extracts the number of elements strictly lower than m. The Rust implementations of these operations on our Elias-Fano representation are presented in Supplementary Figures 24 and 22. The complexity of the select mainly depends on the implementation of *find_one_of_index* since all the other operations take constant time. Similarly, the complexity of the rank depends on the implementation of *find_zero_of_index*. Therefore, to have **rank** and **select** in constant time we need to obtain a constant computational time for both *find_one_of_index* and *find_zero_of_index*. Both functions need to find the i-th one/zero in a bit-array, so that the naive way to solve the problem would be to scan the array from the start and, at each step, count how many values $v \in \{0, 1\}$ have been encountered so far. This algorithm scales linearly with the length of the bit-vector and thus is not practical for large arrays. A simple solution to improve the linear scan is to get a starting point that is closer to the result. To do so, given a bit-array to scan, we choose a quantum q and store the position of every q-th value $v \in \{0, 1\}$ into an auxiliary array $O = [o_0, o_1, \ldots]$. Therefore, to find the i-th value v in Elias-Fano's high-bits array, the linear scan will start from position o_k, where $k = \lfloor i/q \rfloor$, and it will have to scan at most until the next position o_{k+1}. Considering that Elias-Fano's high-bits array contains approximately half uniformly distributed ones and half uniformly distributed zeros, the average distance between two consecutive values $v \in \{0, 1\}$ is equal to 2 bits, which implies that the average distance between two consecutive positions o_k and o_{k+1}, that is the maximum number of bits to scan for searching a the i-th

[8] full implementation at `https://github.com/zommiommy/elias_fano_rust/blob/develop/src/concurrent_builder.rs`

value v, is $\mathbb{E}[o_{k+1} - o_k] = 2q$. Therefore, if the high-bits array has n bits, by using the auxiliary vector O, the average time complexity in the worst case is reduced from $\mathcal{O}(n)$ to $\mathbb{E}[o_{k+1} - o_k] = \mathcal{O}(q) = \mathcal{O}(1)$. Of note, we can further speed up the linear can by computing the number of vs in a word of memory (64-bits) using the **popcnt** instruction, thus allowing us to skip 64 bits each time. Finally, if the CPU supports the BMI2 instruction set, we can use the instructions **pdep** and **tzcnt** to find the wanted value v in a word in constant time (5 cycles) [9]. For what regards the memory complexity of the proposed index, we need on average $\mathcal{O}\left(\frac{n}{2q}\right)$ integers for storing the positions of the ones (or of the zeros), and each integer position needs at most $\lceil \log_2 n \rceil$ bits to be stored. Therefore, each of the two indexes (one index for the ones to speed the select, and one index for the zeros to speed the rank) cause a memory occupation, in the worst case, of $\mathcal{O}\left(\frac{n}{2q}\log_2 n\right)$ bits, which resolves to a total worst-case memory occupation of $\mathcal{O}\left(\frac{n}{q}\log_2 n\right)$ bits. Note that while indices with memory complexity of $o(n)$ exist, a careful implementation allows the use of a relatively high value for q, which practically results in low overhead. In particular to store KG Covid, which has around 450'000 nodes and 32'000'000 edges, Elias-Fano uses 56Mib to store the adjacency matrix, of which 0.6 Mib are for the **1s** and **0s** indices. Therefore, the overhead ratio of the indices is 1.07% when compared to the size of the whole structure. On this KG Covid we are able to perform ranks in 50ns and selects in 118 ns on a Ryzen 9 3900x.

[9] On Skylake CPUs, **pdep** has a latency of 3 cycles, **tzcnt** has a latency of 1 cycles in this use-case and **shlx** (the instruction needed for the binary shift to the left) has latency of 1 cycle, therefore obtaining a total latency of 5 cycles.

```rust
/// Returns position of `index`-th bit set to one.
pub fn select1(&self, index: u64) -> u64 {
    // use the index to find
    // in which block the value is
    let mut reminder_to_scan = index & INDEX_MASK;
    let idx = (index >> INDEX_SHIFT) as usize;
    // the bit position of the biggest
    // multiple of INDEX_SIZE which is
    // smaller than the choosen index,
    // this is were we will start our search
    let pos = self.high_bits_index_ones[idx];
    // find in which word the start value is
    let mut block_id = (pos >> WORD_SHIFT) as usize;
    let in_word_reminder = pos & WORD_MASK;
    // build the standard word to start scanning
    let mut code = self.high_bits[block_id];
    // clean the "already parsed lower bits"
    code &= u64::MAX << in_word_reminder;
    // use popcnt to find the right word
    loop {
        let popcnt = code.count_ones() as u64;
        if popcnt > reminder_to_scan {
            break
        }
        block_id += 1;
        reminder_to_scan -= popcnt;
        code = self.high_bits[block_id];
    }
    // Find index of `reminder_to_scan`-th
    // one in `code`
    let in_word_index = select1_in_word(
        code,
        reminder_to_scan
    );

    (block_id * WORD_SIZE) + in_word_index
}
```

```rust
/// Find index of `index`-th one in word
pub fn select1_in_word(
    word: u64,
    index: u64
) {
    // If the cpu supports
    // the BMI2 instruction set
    // use the optimized version
    // that exploits PDEP
    #[cfg(target_feature="bmi2")]
    unsafe {
        return core::arch::x86_64::_pdep_u64(
            1_u64 << n, x
        ).trailing_zeros() as u64;
    }
    // otherwise fall down
    // to the generic version
    #[cfg(not(target_feature="bmi2"))]
    {
        for _ in 0..reminder_to_scan {
            // reset the lowest set bits
            // if the cpu supports BMI1
            // this is transalted to
            // a `BLSR` instruction
            code &= code - 1;
        }
        return code.trailing_zeros() as u64;
    }
}
```

Supplementary Figure 20: **Select implementation with Rust** in this implementation we omitted the logic needed to handle all the corner-cases. The full implementation can be found at https://github.com/zommiommy/elias_fano_rust/blob/master/src/elias_fano.rs

DataStructure	Select Time (ns)
Vec	17
**EliasFano**	**120**
Indexed BitVec	906
Rank9	2'362
Jacobson	3'266
Fid	8'722
RsDict	12'021

DataStructure	Rank Time (ns))
Rank9	9
Jacobson	19
**EliasFano**	**50**
Indexed BitVec	51
Vec	72
Fid	96
RsDict	113

DataStructure	Memory (Mib)
**EliasFano**	**56**
Vec	256
RsDict	13'344

Supplementary Table 49: Benchmarks of our implementation of Elias-Fano against other data-structures on storing the KgCovid graph. Elias-Fano offers good performances in both time and memory. While RsDict offers good compression for sparse bit-vectors, the adjacency matrix of KgCovid is really sparse as only 0.015% of the bits are ones which could be a degenerate case for this data-structure.

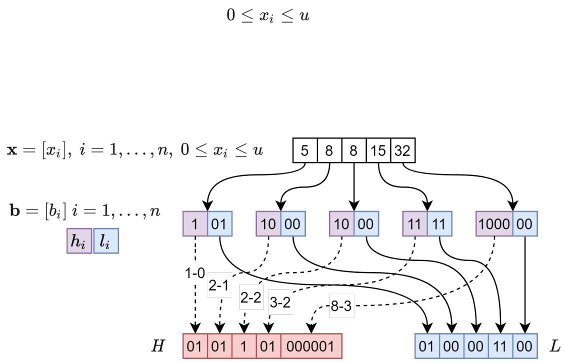

Supplementary Figure 21: **Example of Elias-Fano.** Elias-Fano splits the sorted values into high and low bits; the low-bits parts are then consecutively copied in the low-bits array, L (on the right, blue color), while the high-bits parts are coded into an high-bits array H (on the left, red color) by consecutively storing the gaps between consecutive high-bits parts, encoded by using the inverted unary representation.

```
/// Find index-th smallest value
fn select(&self, index: u64) -> u64 {
    // find the index of the high-th
    // one in the bit-vector
    // and subtract the index
    // to obtain the number of
    // zeros before the index-th one.
    let high = self.high_bits.find_one_of_index(
        index
    ) - index;
    // get the lower bits of the value
    let low  = self.read_lowbits(index);
    // merge the high and low bits
    (high << self.low_bits_size) | low
}
```

Supplementary Figure 22: **Rust implementation of a simplified select operation:** in this implementation we omitted the logic needed to handle all the corner-cases. The full implementation can be found at https://github.com/zommiommy/elias_fano_rust/blob/master/src/elias_fano.rs

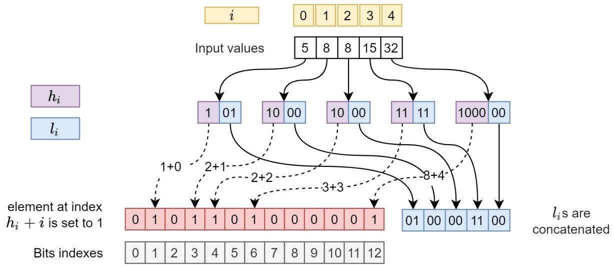

Supplementary Figure 23: **Algorithm for Elias-Fano encoding presented in [36].** While the low-bits array (on the right, blue color) is simply composed by sequentially concatenating the l_is for each $i = 1, \ldots, n-1$, the high-bits array is composed by setting the bit at index $h_i + i$ to 1.

```
/// Return number of elements smaller than value
fn rank(&self, value: u64) -> u64 {
    // split the value into
    // its higher and lower bits
    let high = value >> self.low_bits_size;
    let low = value & self.low_bits_mask;
    // find the index of the
    // high-th zero in the bit-vector
    let mut index = self.high_bits.find_zero_of_index(
        high
    );
    // start scanning the lower
    // bits to find the first element
    // bigger or equal than value
    while (
        self.high_bits[index] == 1 &&
        self.read_lowbits(index - high) < low
    ){
        index += 1;
    }
    // the number of elements is equivalent
    // to the index of the value
    // in the high-bits
    // minus the higher bits because
    // the count is the number
    // of ones before index and
    // high is the number of zeros before index.
    index - high
}
```

Supplementary Figure 24: **Rust implementation of a simplified rank operation:** in this implementation we omitted the logic needed to handle all the corner-cases. The full implementation can be found at https://github.com/zommiommy/elias_fano_rust/blob/master/src/elias_fano.rs

7.1.1 Edge encoding

In this subsection we describe how *Ensmallen* converts all the edges of a graph $G(V, E)$ into a sorted list of integers. In particular, considering an edge $e = (v, x) \in E$ connecting nodes v and x represented, respectively with integers a and b, the binary representation of a and b are concatenated through the function $\phi_k(a, b)$ to generate an integer index uniquely representing the edge e itself:

$$\phi_k(a,b) = a\ 2^k + b, \text{ where } k = \lceil \log_2 |V| \rceil \qquad \Rightarrow \qquad a = \left\lfloor \frac{\phi_k(a,b) - b}{2^k} \right\rfloor, \qquad b = \phi_k(a,b) - a\ 2^k$$

This implementation is particularly fast because it requires only few bit-wise instructions:

$$\phi_k(a,b) = a << k|b \qquad \Rightarrow \qquad a = \phi_k(a,b) >> k, \qquad b = \phi_k(a,b)\ \&\ (2^k - 1)$$

where $<<$ is the left bit-shift, $|$ is the bit-wise OR and & is the bit-wise AND. Since the encoding uses $2k$ bits, it has the best performances when it fits into a CPU word, which is usually 64-bits on modern computers, meaning that the graph must have less than 2^{32} nodes and and less than 2^{64} edges. However, by using multi-word integers it can be easily extended to even larger graphs [38]. As an example, considering a graph with at most 8 nodes, encoded with integers numbers ($v \in [0, \ldots, 7]$) In Supplementary Figure 25 we schematize the encoding of the edge $(2, 6)$ which has 2 as source node, and 6 as destination node. On the right we report the Rust implementation of the edge encoding and decoding. Once the edges are encoded, we can sort them and use Elias-Fano to store them.

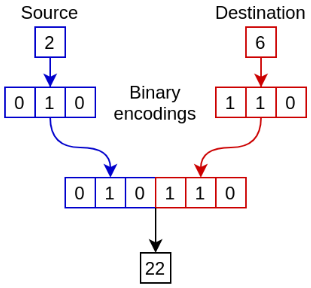


```rust
fn encode(src: u64, dst: u64, k: u64) -> u64 {
    (src << k) | dst
}
fn decode(edge: u64, k: u64) -> (u64, u64) {
    let src = edge >> k;
    let dst = edge & ((1 << k) - 1);
    (src, dst)
}
```

Supplementary Figure 25: On the left, an example of the encoding. On the right its implementation in Rust.

7.2 Specialized Random-Walks

While computing a second-order random walk, Leskovec et al. [39], when the walk has stepped from node t to node v, where it now resides, the next step is guided by the un-normalized transition probability π_{vx} of moving from v to x. π_{vx} is a function of the weight w_{vx} of the edge (v, x), and the search bias $\alpha_{pq}(t, x)$:

$$\pi_{vx} = \alpha_{pq}\ (t, x)\ w_{vx}$$

In Supplementary Figure 26 the node2vec schema for defining the search bias is schematized.

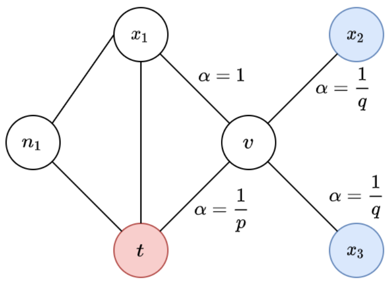

Supplementary Figure 26: **Illustration of the random walk procedure in node2vec.** The walk just transitioned from t to v and is now evaluating on which node to step next. Edge labels indicate search biases α. The nodes in blue are at distance 2 from t, so that the edge connecting them to v has α equal to the explore (in-out) bias; red nodes, at distance 1 from t, are connected to v by and edge with α equal to the return bias.

As explained in Section 4.1.4 of the main paper the in-out bias can be re-formulated to allow an efficient implementation: starting from an edge (t, v) we need to compute $\beta_q(t, x)$ for each $x \in N(v)$, where $N(v)$ is the set of nodes adjacent to v including node v itself.

$$\beta_q(t, x) = \begin{cases} 1 & d(t, x) \leq 1 \\ \frac{1}{q} & d(t, x) = 2 \end{cases} \qquad \Rightarrow \qquad \beta_q(t, x) = \begin{cases} 1 & x \in N(t) \\ \frac{1}{q} & x \notin N(t) \end{cases}$$

This formulation (Supplementary Figure 27c) allows us to compute in batch the set of nodes X_β affected by the in-out parameter q:

$$X_\beta = \left\{ x \mid \beta_q(t, x) = \frac{1}{q}, q \neq 1 \right\} = N(v) \setminus N(t)$$

Therefore the selection of the nodes X_β affected by β_q can be reduced to computing the difference of the two sets $N(v) \setminus N(t)$ ($N(t)$ is the neighborhood of t already computed at the preceding step. X_β is efficiently computed by using a SIMD algorithm implemented in assembly, leveraging AVX2 instructions that work on node-set representations as sorted vectors of the indices of the nodes (see Supplementary Figure 29). The algorithm is adapted from Lemire's et al. [40] SIMD algorithm for set intersection, which similarly works on sets represented as sorted arrays.

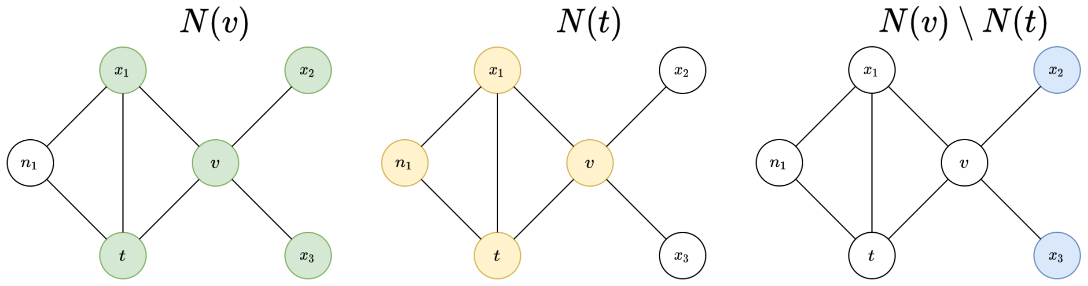

(a) Neighbours of v (b) Neighbours of t (c) The left difference of Neighbours

Supplementary Figure 27: **Illustration of the equivalence with Leskovec formulation.** The walk just transitioned from t to v and is now evaluating its next step out of node v. The green nodes are the neighbours of v, the yellow nodes are the neighbours of t, already computed in the previous step of the walk. The blue nodes are those effected by the in-out bias (also in Supplementary Figure 26), which may be computed as the difference of $N(v)$ and $N(t)$.

Depending on the values of p, q and on the type of the graph (weighted or unweighted), *Ensmallen* provides eight different specialized implementation of the Node2Vec algorithm, detailed in Supplementary Table 50. This trick allows to significantly speed-up the computation, as shown by the empirical computational times reported in Supplementary Table 51, which were obtained by each of the specialized algorithms when computing 1000 random walks of length 100 on the KGCovid19 Graph, using an AMD Ryzen 9 3900x processor.

Supplementary Table 50: **Specialized first and second-order random walks algorithms:** the 8 different algorithms, dynamically dispatched by the library according to the use case.

	$q = 1$		$q \neq 1$	
	$p = 1$	$p \neq 1$	$p = 1$	$p \neq 1$
**Unweighted Graph**	Unweighted first-order random walk	Unweighted second-order return-weight-only random walk	Unweighted second-order explore-weight-only random walk	Unweighted second-order random walk
**Weighted Graph**	Weighted first-order random walk	Weighted second-order return-weight-only random walk	Weighted second-order explore-weight-only random walk	Weighted second-order random walk

Supplementary Table 51: Empirical Computational time required by the different optimized implementations for computing the transition probability π_{vx} listed in Supplementary Table 50. The computational time was measured when computing 1000 random walks of length 100 on the KGCovid19 Graph, using an AMD Ryzen 9 3900x processor.

q	p	Graph	π_{vx}	Time (ms)
$q = 1$	$p = 1$	Unweighted	1	0.46 (±0.01)
$q = 1$	$p = 1$	Weighted	w_{vx}	0.50 (±0.01)
$q = 1$	$p \neq 1$	Unweighted	$\gamma_p(t, x)$	13.8 (±0.08)
$q = 1$	$p \neq 1$	Weighted	$\gamma_p(t, x)w_{vx}$	14.2 (±0.07)
$q \neq 1$	$p = 1$	Unweighted	$\beta_q(t, x)$	45.8 (±0.5)
$q \neq 1$	$p = 1$	Weighted	$\beta_q(t, x)w_{vx}$	47.3 (±1)
$q \neq 1$	$p \neq 1$	Unweighted	$\beta_q(t, x)\gamma_p(t, x)$	47.7 (±0.2)
$q \neq 1$	$p \neq 1$	Weighted	$\beta_q(t, x)\gamma_p(t, x)w_{vx}$	49.0 (±0.3)

7.2.1 Faster Pseudo-Random Numbers Generators (Vectorized xorshift)

Many of the algorithms inside of *Ensmallen*, e.g. the sampling of destination nodes during the random walk, or the generation of random negative edges for Skipgram [6] model, rely on the generation of random numbers. Therefore, a random number generator algorithm could be a bottleneck if not efficiently implemented. To guarantee efficiency, in *Ensmallen* we use the two following fast pseudo-random number generators, Vigna's Xoshiro256+ [41] and the Marsaglia's Xorshift [42]. Xoshiro256+ has a shallower dependencies chain than Xorshift, which results in lower latency, while Xorshift has fewer instruction than Xoshiro256+, so that it can be implemented to achieve higher throughput. Therefore, Xoshiro256+ is faster in the generation of a single random value, e.g. during the sampling of the destination node in a step of the random walk. On the other side, Xorshift is faster in the generation of multiple random numbers, e.g. when generating the random negative edges for Skipgram. Modern CPUs allow to execute up to 4 different independent instructions in the same cycle and can have eight or more memory requests running at a time, so a common way to exploit the super-scalarity of modern CPUs and out-of-order execution is to interleave different instances of the same algorithm. This reduces the importance of the depth of dependency chain of an algorithm and favors the number of instructions. For these reasons, we interleave eight different instances of Xorshift and, to further improve its throughput, we implemented a vectorized version which exploits Intel's AVX2 instruction sets to execute 4 instances in parallel. An non-interleaved example of the vectorized Xorshift is illustrated in Supplementary Figure 28. With the aforementioned implementation, we achieve a throughput of 256 random bytes (32 64-bits integers) at the cost of 4 concurrent cache misses (which are adjacent so the values should already be in the L1 cache). The complete implementation is available in Supplementary Figure 29 and achieves a throughput of more than 20 times higher than standard methods as shown in Supplementary Table 52.

```rust
pub fn xorshift(mut seed: u64) -> u64 {
    seed ^= seed << 13;
    seed ^= seed >> 7;
    seed ^= seed << 17;
    seed
}
```

```asm
vmovdqu ymm0, ymmword ptr [rsi]
vpsllq ymm1, ymm0, 13
vpxor ymm0, ymm0, ymm1
vpsrlq ymm1, ymm0, 7
vpxor ymm0, ymm0, ymm1
vpsllq ymm1, ymm0, 17
vpxor ymm0, ymm0, ymm1
vmovdqu ymmword ptr [rsi], ymm0
```

Supplementary Figure 28: On the left the classic xorshift algorithm is reported. On the right we report the vectorized xorshift which uses AVX2 to execute, through data parallellism, 4 64bits xorshifts.

Supplementary Table 52: The time taken by each method to generate a batch 32000 random 64-bits integers. This benchmark was executed on a single thread using Rust's default benchmark library which collects at least 50 samples. The performance difference, between the two considered CPUs, might be due to the fact that the AVX2 logical shifts instructions (vpsllq and vpsrlq) on Coffe Lake have twice the throughput when compared to Zen2.

	I7-8750H 4.1Ghz		Ryzen 3900x 4.0Ghz	
Method	Total Time (μs)	Throughput $\left(\frac{GB}{s}\right)$	Total Time (μs)	Throughput $\left(\frac{GB}{s}\right)$
thread rng	367.3 (±20.8)	0.7	349.3 (±20.66)	0.73
xorshift	48.1 (±7.89)	5.3	48.0 (±0.067)	5.3
xorshift avx2	36.9 (±1.88)	6.9	48.0 (±0.043)	5.3
xorshift avx2 4 interleaved	9.6 (±0.76)	26.7	14.5 (±0.014)	17.6
xorshift avx2 8 interleaved	10.0 (±1.07)	25.6	14.0 (±0.353)	18.3

```
; Load the data
vmovdqu ymm0, ymmword ptr [rsi]
vmovdqu ymm2, ymmword ptr [rsi + 32]
vmovdqu ymm4, ymmword ptr [rsi + 64]
vmovdqu ymm6, ymmword ptr [rsi + 96]
vmovdqu ymm8, ymmword ptr [rsi + 128]
vmovdqu ymm10, ymmword ptr [rsi + 160]
vmovdqu ymm12, ymmword ptr [rsi + 192]
vmovdqu ymm14, ymmword ptr [rsi + 224]
; << 13
vpsllq ymm1, ymm0, 13
vpsllq ymm3, ymm2, 13
vpsllq ymm5, ymm4, 13
vpsllq ymm7, ymm6, 13
vpsllq ymm9, ymm8, 13
vpsllq ymm11, ymm10, 13
vpsllq ymm13, ymm12, 13
vpsllq ymm15, ymm14, 13
; ^
vpxor ymm0, ymm0, ymm1
vpxor ymm2, ymm2, ymm3
vpxor ymm4, ymm4, ymm5
vpxor ymm6, ymm6, ymm7
vpxor ymm8, ymm9, ymm1
vpxor ymm10, ymm11, ymm3
vpxor ymm12, ymm13, ymm5
vpxor ymm14, ymm15, ymm7
; >> 7
vpsrlq ymm1, ymm0, 7
vpsrlq ymm3, ymm2, 7
vpsrlq ymm5, ymm4, 7
vpsrlq ymm7, ymm6, 7
vpsrlq ymm9, ymm8, 7
vpsrlq ymm11, ymm10, 7
vpsrlq ymm13, ymm12, 7
vpsrlq ymm15, ymm14, 7
; ^
vpxor ymm0, ymm0, ymm1
vpxor ymm2, ymm2, ymm3
vpxor ymm4, ymm4, ymm5
vpxor ymm6, ymm6, ymm7
vpxor ymm8, ymm9, ymm1
vpxor ymm10, ymm11, ymm3
vpxor ymm12, ymm13, ymm5
vpxor ymm14, ymm15, ymm7
; << 17
vpsllq ymm1, ymm0, 17
vpsllq ymm3, ymm2, 17
vpsllq ymm5, ymm4, 17
vpsllq ymm7, ymm6, 17
vpsllq ymm9, ymm8, 17
vpsllq ymm11, ymm10, 17
vpsllq ymm13, ymm12, 17
vpsllq ymm15, ymm14, 17
; ^
vpxor ymm0, ymm0, ymm1
vpxor ymm2, ymm2, ymm3
vpxor ymm4, ymm4, ymm5
vpxor ymm6, ymm6, ymm7
vpxor ymm8, ymm9, ymm1
vpxor ymm10, ymm11, ymm3
vpxor ymm12, ymm13, ymm5
vpxor ymm14, ymm15, ymm7
; Store the data
vmovdqu ymmword ptr [rdi], ymm0
vmovdqu ymmword ptr [rdi + 32], ymm2
vmovdqu ymmword ptr [rdi + 64], ymm4
vmovdqu ymmword ptr [rdi + 96], ymm6
vmovdqu ymmword ptr [rdi + 128], ymm8
vmovdqu ymmword ptr [rdi + 160], ymm10
vmovdqu ymmword ptr [rdi + 192], ymm12
vmovdqu ymmword ptr [rdi + 224], ymm14
```

Supplementary Figure 29: The complete implementation of the 8 way interleaved AVX2 xorshift. For compactness, the code is divided in two columns, the intended order is from left to right that uses the vectorized xorshift described in Supplementary Section 7.2.1.

7.2.2 Efficient cumulative sum computation

The cumulative sum, also called prefix sum, is one of the most known examples in the field of parallel computing; in *Ensmallen* it is fundamental during the computation of random walks, to compute the un-normalized cumulative sum of the un-normalized transition probabilities to each node (see Section 4.1.4 of the main paper). To obtain efficient computations, *Ensmallen*'s implementation exploits the Streaming SIMD Extensions (SSE 128-bit) instruction set, which is an extension of the SIMD instruction set for the x86_64 architecture. CPUs with SSE support have a special set of 8 128-bits registers, on which vector operations may be executed, that operate in parallel on 4 floats, therefore increasing the throughput by 4 times. The algorithm is based on two SSE instructions: *vaddps* (Supplementary Figure 30a), which provides element-wise sum of two registers, each containing 4 32-bits floats, and *vpalignr* (Supplementary Figure 30b), which concatenate two registers into a temporary register, shifts the result by a chosen number of bytes and then returns the lower 128-bits. The generalization of the algorithm for wider instruction sets such as AVX2 (256-bit) or AVX512 (512-bit) is limited by the lack of multi-lane logical shifts (the vpalignr instruction) which can be avoided by using opportunely shuffle and permutation instructions which introduces additional latency so the throughput don't scales linearly. While this does not improve the complexity of the algorithm it is almost ten times faster than the naive implementation and five time faster than the unrolled version. Loop unrolling [43] is an optimization technique for tight loops which increase the number of steps executed for each cycle, this reduces the overhead for the loop, allows to better exploit the CPU super-scalarity and reduces the number of branches. This technique increase the size of the function and thus if abused might degrade performances due to cache missing.

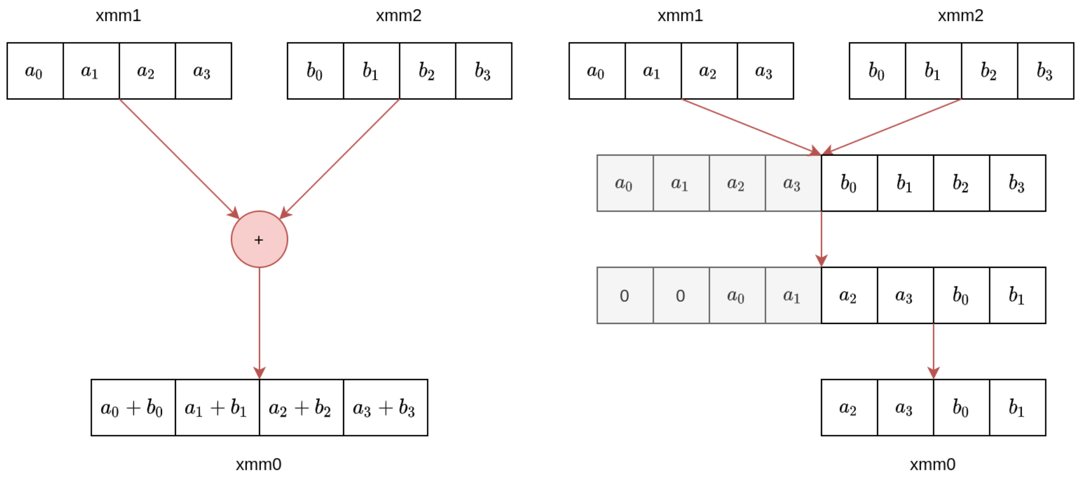

(a) vaddps xmm0, xmm1, xmm2

(b) vpalignr xmm0, xmm1, xmm2, 2

Supplementary Figure 30: **The two fundamental SIMD instructions for the prefix sum.** Left: *vaddps* computes the elementwise sum of two registers, each containing 4 floats represented by 32 bits. Right: *vpalignr* concatenates two registers, shifts the result by a number of bytes provided as input and returns the lower 128-bits.

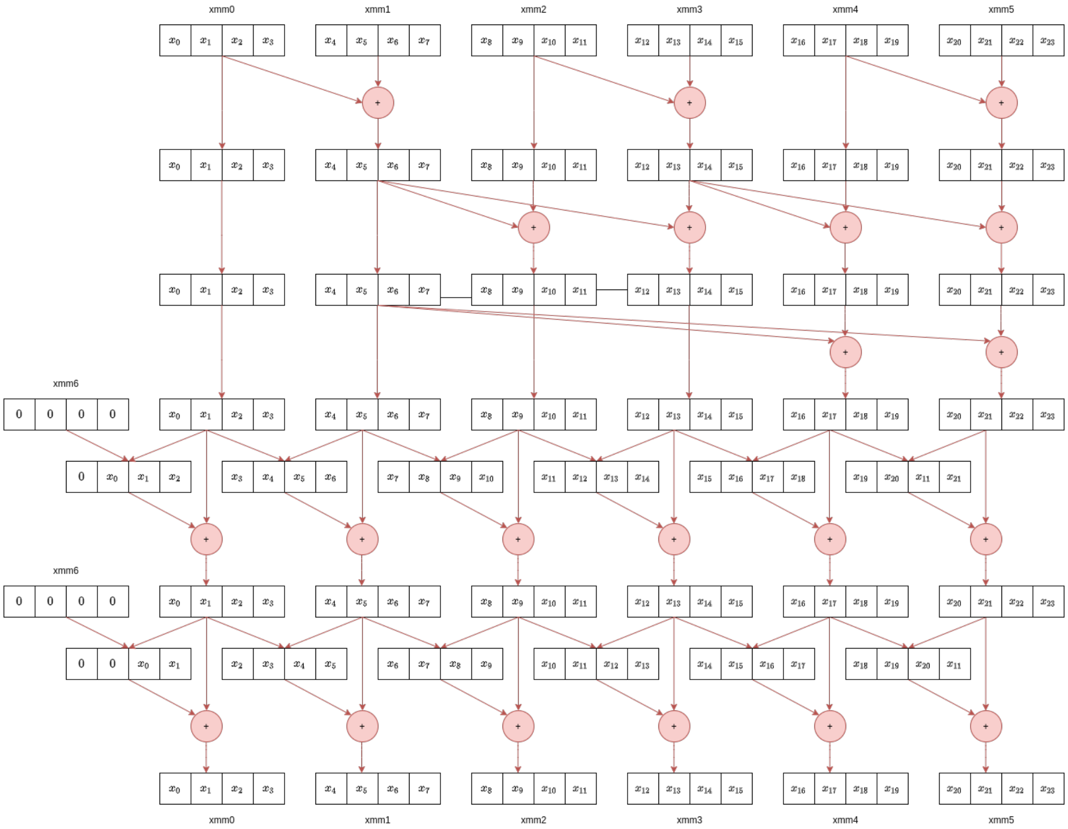

Supplementary Figure 31: **SIMD prefix sum algorithm.** To compute the prefix-sum of 24 float values, the prefix-sum we implemented uses mainly the *vaddps* and the *vpalignr* instructions. Note that, in the diagram the *vpalignr* instruction is represented in a compact way.

```
; first stage
vaddps xmm1, xmm0, xmm1
vaddps xmm2, xmm2, xmm3
vaddps xmm5, xmm4, xmm5
; second stage
vaddps xmm5, xmm3, xmm5
vaddps xmm4, xmm3, xmm4
vaddps xmm3, xmm1, xmm3
vaddps xmm2, xmm1, xmm2
; third stage
vaddps xmm5, xmm1, xmm5
vaddps xmm4, xmm1, xmm4
; fourth stage
vpalignr xmm7,  xmm0, xmm6, 12
vpalignr xmm8,  xmm1, xmm0, 12
vpalignr xmm9,  xmm2, xmm1, 12
vpalignr xmm10, xmm3, xmm2, 12
vpalignr xmm11, xmm4, xmm3, 12
vpalignr xmm12, xmm5, xmm4, 12
vaddps xmm0, xmm0, xmm7
vaddps xmm1, xmm1, xmm8
vaddps xmm2, xmm2, xmm9
vaddps xmm3, xmm3, xmm10
vaddps xmm4, xmm4, xmm11
vaddps xmm5, xmm5, xmm12
; fifth stage
vpalignr xmm7,  xmm0, xmm6, 8
vpalignr xmm8,  xmm1, xmm0, 8
vpalignr xmm9,  xmm2, xmm1, 8
vpalignr xmm10, xmm3, xmm2, 8
vpalignr xmm11, xmm4, xmm3, 8
vpalignr xmm12, xmm5, xmm4, 8
vaddps xmm0, xmm0, xmm7
vaddps xmm1, xmm1, xmm8
vaddps xmm2, xmm2, xmm9
vaddps xmm3, xmm3, xmm10
vaddps xmm4, xmm4, xmm11
vaddps xmm5, xmm5, xmm12
```

Supplementary Figure 32: **Inner core of the SIMD prefix sum algorithm** This code assumes that in in the registers from xmm0 to xmm5 are loaded with the data and that xmm6 is zero filled. The result will, also, be in the registers from xmm0 to xmm5. We need 13 xmm registers but the SSE standard only provide 8 of them, luckily the AVX expansion increase this number to 16, thus making this algorithm possible.

7.2.3 Alias method

The alias method [5] efficiently samples k integers from a discrete probability distribution. The algorithm is used since it requires $\mathcal{O}(n)$ steps in the pre-processing phase (when Vose's algorithm is used [44]) and $\mathcal{O}(1)$ steps for each point sampling. The algorithm is sketched in Supplementary Figure 33, using a discrete probability distribution $[p_0, \ldots, p_{n-1}]$ where each p_i is the probability of sampling an integer $i \in [0, \ldots, n-1]$ ($n = 4$ in Supplementary Figure 33). The heights of the bars correspond to $[p_0, \ldots, p_{n-1}]$ and their width is one, so that the histogram area is 1. Point sampling from the depicted distribution requires to draw a random value $0 \leq u \leq 1$ and find the segment (from p_0 to p_1, from p_1 to p_2, and so on) where u falls. This check could be performed by the following comparisons: if $u < p_0$ then pick 0; otherwise, if $p_0 \leq u < p_0 + p_1$ then sample 1; otherwise, if $p_0 + p_1 \leq u < p_0 + p_1 + p_2$ then sample 2; otherwise if $u \geq p_0 + p_1 + p_2$ then sample 3. This would be computationally expensive since each sampling would require, in the worst case, $n - 1$ comparisons. To reduce the computational complexity, a more efficient algorithm could be designed that draws 2D points falling in the gray rectangle shown in Supplementary Figure 33-b, where each bar represents the probability of sampling of one integer value according to the discrete probability distribution, or, in other words, each bar represents a sampling event. The algorithm needs only to check whether the drawn point falls in any of the bars. To this aim, an iterative process could be used that exploits only an indexed array with the discrete probabilities $P_{vec} = [p_0, \ldots, p_{n-1}]$ of each integer. Each iteration should draw two values: an index $0 \leq s \leq n$, that selects one of the elements of the array P_{vec}, and a random value $0 \leq u \leq 1$. The drawing continues until the drawn s and u are such that $u \leq P_{vec}[s]$, which leads to sampling s. In Supplementary Figure 33-b the index $s = 1$ and the value (corresponding to the gray dot) $u \geq P_{vec}[1]$ correspond to a miss (the gray dot) that does not allow selecting 1 as the drawn value; conversely, the green dot corresponds to a drawn index $s = 3$ and a drawn $u \leq P_{vec}[3]$, which allows selecting the value 3 as the sampled value. However, as shown in Supplementary Figure 33-b, usage of the aforementioned method has the disadvantage of having an "empty space" that would cause repeated iterations, therefore increasing the computational time. To guarantee success at each draws, in [5] authors propose composing a rectangle where the probabilities ("rectangles" in the Supplementary Figure) are rearranged by splitting them, so that no empty spaces are left and each column contains at (Supplementary Figure 33-c). In this way, each drawing of a 2D point would always fall into a decision region, therefore bringing to a decision. To this aim, the alias method applies a pre-processing phase, generally performed through Vose's algorithm [44] that, given the range $[0 - max]$ among which to sample, and the number n of integers to sample, computes two indexed vectors: the separating probability vector $P_{sep} = [p_0^{sep}, \ldots, p_{n-1}^{sep}]$, and the "other class" (also called alias) vector $OC = [oc_0, \ldots, oc_{n-1}]$. The algorithm then draws a unique point $0 \leq x \leq 1$, from which it then computes an integer $s = \lfloor nx \rfloor$, uniformly distributed in $0, 2, \ldots, n-1$, and a value $u = nx + 1 - s$ uniformly distributed on $[0, 1)$. If $u \leq P_{sep}[s]$ the alias method samples s, otherwise it samples $OC[s]$. Though efficient in the sampling process, when the range from which to sample becomes high, the pre-processing phase for computing the separating probabilities and the alias vector is impractical. Therefore, in *Ensmallen* we have implemented the SUSS algorithm, described in Supplementary Section 7.2.4.

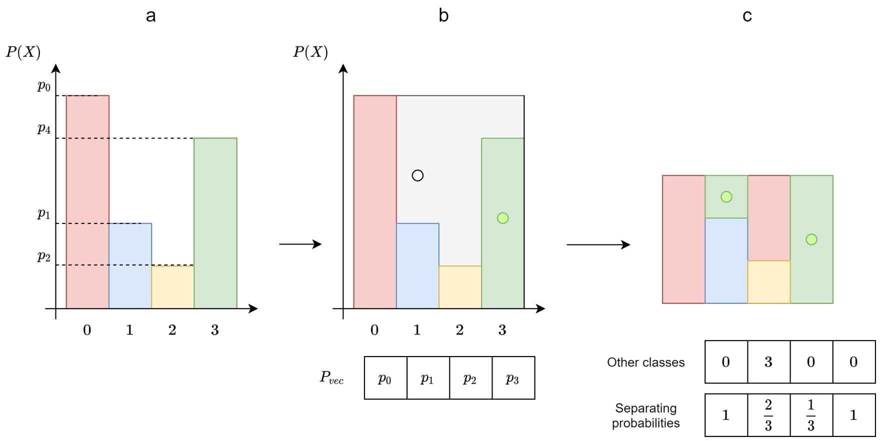

Supplementary Figure 33: **The Alias method**. Left: a discrete probability distribution. Center: a quasi-efficient sampling method containing empty spaces. Right: the alias method.

7.2.4 SUSS: Sorted Unique Sub-Sampling

The Sorted Unique Sub-Sampling algorithm (SUSS) has been designed in *Ensmallen* to allow sub-sampling k unique sorted integers among n integers, by following an approximate uniform distribution. After splitting the range $[0, \ldots, n-1]$ into k equal segments (buckets) with length $\lfloor \text{delta}/k \rfloor$, SUSS samples an integer from each bucket by using Xorshift random number generator. The implementation of the algorithm is reported in Supplementary Algorithm 1. To establish whether the distribution of the integers sampled with SUSS is truly approximating an uniform distribution, we sampled $n = 10.000.000$ integers over $[0, \ldots, 10.000]$, by using both SUSS and by drawing from a uniform distribution in $[0, \ldots, 10.000]$. We then used the Wilcoxon signed-rank test to compare the frequencies of the obtained indices and we obtained a p-value of 0.9428, meaning that there is not a statistically significant difference between the two distributions. Therefore, by using a time complexity $\Theta(k)$ and a spatial complexity $\Theta(k)$ SUSS produces reliable approximations of a uniform distribution.

Algorithm 1: Sorted Unique Sub-Sampling (SUSS)

input : Minimum value of range min_val
Maximum value of range max_val
Quantity of values to sample k
Seed to reproduce the sampling s
output: Sorted list of extracted unique indices

extracted $\leftarrow$ [];
delta $\leftarrow$ max_val $-$ min_val;
step $\leftarrow \lfloor \text{delta}/k \rfloor$;
for $i \leftarrow 0; i < k-1; i \leftarrow i+1$ **do**
  extracted.push(min_val $+$ step $* \, i + s \,\%\,$ step);
  $s \leftarrow$ xorshift(s);
end
extracted.push(max_value $- \, s \,\%\,$ (delta $-$ step $* \, (k-1)) - 1$);
return extracted

7.3 Methods for accurately measuring RAM peak and time requirements

For properly measuring the peak memory usage and the time requirements of both *Ensmallen* and the state-of-the-art libraries we use as benchmark comparison, we created an additional thread for logging purposes. We have measured the used memory by reading `/proc/meminfo`, which makes available five different metrics:

MemTotal RAM installed on the system

MemFree RAM not used

Buffers RAM used for I/O buffers

Cached RAM used for dirty pages and ramdisks

Slab RAM used by the kernel

We define the memory in use as: $\text{MemInUse} = \text{MemTotal} - \text{MemFree} - \text{Buffers} - \text{Cached} - \text{Slab}$. We executed *Ensmallen* and all the benchmarks on a dedicated server with no significant running process, except the sshd service we use to connect to it. Anyhow, to obtain a truthful evaluation of the memory usage due to the execution of a specific task, we have logged the average memory usage before the task starts, and we have subtracted such value from the memory usage we measure during the task execution. There are more accurate methods such as jemalloc [45], Valgrind [46], hooking malloc using `__malloc_hook` or using `LD_PRELOAD` to hook the malloc function, but this method is precise enough since we do not need to measure the difference in bytes, but we care about significant differences. We designed the tracker to have a linearly increasing delay between measurements because we have to measure tasks that might take from few microseconds to hours: long-running tasks would otherwise log too much data and start using Gigabytes of RAM. To further reduce this problem, we log the values in a constant size buffer, and when either the task finishes or the delay between measurement is significantly longer than the time necessary to write the log to disk, we dump the log to a file.

8 Overview of techniques implemented in *GRAPE*

8.1 Spectral and matrix factorization embedding methods

Laplacian Eigenmap (LE) computes the symmetrically normalized Laplacian and then computes as embeddings the eigenvectors corresponding to the k *smallest* eigenvalues. Building on top of LE, Geometric Laplacian Eigenmap Embedding (GLEE) [47], also computes the symmetrically normalized Laplacian and then computes the eigenvectors corresponding to the k *largest* eigenvalues. High-Order Proximity preserved Embedding (HOPE) [48] starts by computing a node-proximity matrix, where the proximity between two nodes may be defined in different ways, e.g. by using the number of common neighbors, or the Adamic-Adar index. Then HOPE computes the singular vectors corresponding to the k most significant singular values of the proximity matrix and uses the left and right product of the singular values with the singular vectors as the embeddings of, respectively, the source and destination nodes. Similar to HOPE, the Social Dimensions (SocioDim) approach computes the **dense** modularity matrix and uses as node embedding the eigenvectors corresponding to the *largest* k eigenvalues [49]. Alternating Direction Method of Multipliers for Non-Negative Matrix Factorization (NMFADMM) [50] leverages NMF to factorize the left Laplacian matrix into two non-negative matrices, which correspond to the embeddings of the source and destination nodes. Similar to NMFADMM, Iterative Random Projection Network Embedding (RandNE) [51] applies an iterative procedure to factorize the dot product of the left Laplacian and an (initially) random matrix R; after a user defined number of factorization the matrix R is used as the node embeddings. Graph Representations (GraRep) [52] analogously factorizes the left Laplacian matrix and, at every iteration, computes the singular vectors corresponding to the k most significant singular values, hence producing several embeddings equal to the number of iterations. The Network Matrix Factorization (NetMF), given a window size, first computes a sparse log co-occurrence matrix by using first-order RWs and then proceeds to compute the singular vectors corresponding to the k largest singular values [53].

8.2 RW-based embedding methods

DeepWalk and its Walklets [54] extension to a multiscale random-walk representation (detailed below) are first-order random-walk sampling methods.

Node2Vec [39], is a second-order RW method that uses weights to bias the walk towards breadth-first search or depth-first search. Node2Vec RWs are more computationally expensive than first-order RWs (see figure 2 **c** and **e** - main paper), since they require to tune two parameters, and our experimental results showed that models trained on Node2Vec walks do not necessarily outperform models trained on first-order walks, when a sufficient amount of training samples is made available (see figure 4 - main paper). This of course depends also on the characteristics of the graph, since it is well-known that by tuning the return and in-out parameters of Node2vec we can capture different topological and structural features of the underlying graph [39].

GloVe [55] trains a two-layer neural network to predict the logarithm of the co-occurrence frequency of two nodes within the contextual window of size w in RWs. CBOW [56] also trains a two-layer neural network to predict the central node of a RW sequence given the other contextual nodes. SkipGram [56] resembles a transposed version of CBOW: it predicts the contextual nodes of a sequence given its central node. Glove, CBOW and SkipGram may be trained with sequences sampled using either DeepWalk or Node2Vec.

Walklets-based SkipGram (or CBOW) computes w times a DeepWalk-based SkipGram (or CBOW) embedding with window size 1. For each $0 \dots i \dots w$ embedding, Walklets filters the random-walks by keeping only nodes whose position within the RW belongs to the congruence class in module i [54]. This is done to obtain node embedding that learn multi-scale RW representations.

Role2Vec with Weisfeiler-Lehman Hashing [57, 58, 59] uses first-order RWs to approximate the point-wise mutual information matrix obtained by multiplying the pooled adjacency power matrix with a structural feature matrix (in this case, Weisfeiler−Lehman features) to obtain a structural node embedding.

8.3 Triple-sampling methods.

Triple sampling methods are shallow neural networks trained on triples, (v, ℓ, s), where $\{v, s\}$ is a node-pair composed of a source (v) and a destination node (s), and ℓ is a property of the edge (v, s) connecting them; this property may, for instance, tell whether the edge exists, or can define the edge type, or its edge weight. They often include a single layer representing the node embedding, and in some models a second layer representing the contextual node embedding or the considered edge properties.

First-order LINE [60] samples node tuples and evaluates whether such tuples correspond to a known edge in the graph. The model optimizes a single layer corresponding to the node embedding; in this way, connected nodes will obtain a *parallel* embedding, while disconnected nodes will obtain an *orthogonal* embedding. Second-order LINE [60] differ from first-order models as they optimize two layers. The first layer corresponds to the source-node embeddings, the second layer corresponds to the destination-node (or contextual-node) embeddings. Analogous to the first-order model, connected source and destination nodes will obtain a parallel embedding, while disconnected nodes will obtain an orthogonal embedding.

8.4 Corrupted triple-sampling methods.

Similar to triple sampling methods, *corrupted*-triple sampling methods are shallow neural networks trained on the (*true*) triples (v, ℓ, s) defined by the existing edges in the graph (where v is the source node, ℓ is the property of the edge (v, s), and s is the destination node, see Supplementary Section 8.3), but also on *corrupted triples*, that are obtained by corrupting the original triples by substituting the source and/or destination nodes $\{v, s\}$ with randomly sampled nodes $\{v', s'\}$, while maintaining the attribute unchanged (v', ℓ, s').

The shallow neural network models used on corrupted-triple sampling batches include a weight matrix representing the node embedding, plus one or more matrices for representing the edge attributes, which are composed to capture the attribute meaning as algebraic operations (e.g. $woman + is\_royal = queen$). For this reason, they are particularly well suited to compute node and edge properties embedding of attributed graphs were the edge properties represent meaningful directed transitions (e.g. *is_royal*), while being out of scope when dealing with local undirected properties (e.g. *interacts with*). Given a distance metric defined for the triples the shallow models are generally optimized to minimize the distance of *true* triples while maximizing the distance of the *corrupted* ones.

The distance defined for triples is often a feature-wise distance, whose advantage is that the computation of the gradient of each feature is independent from any other feature. This allows for particularly effective data-racing-aware and synchronization-free parallel implementations [61].

TransE [61] is among the first and possibly one of the most commonly used of the corrupted-triple sampling methods presented in the literature, from which a large family of variations has been defined. The model trains a shallow neural network composed of two weight matrices representing the node embedding and the edge type embedding. It generally uses as distance metric a feature-wise euclidean distance (though any element-wise distance metric may be used) and defines its energy loss as:

$$\mathcal{L}_{TransE} = \sum_{(v,v',\ell,s,s')} \text{ReLU}\left[\text{constant} + (v + \ell - s)^2 - (v' + \ell - s')^2\right]$$

As per the aforementioned properties of feature-wise distances, our Rust implementation of the TransE model is a synchronization-free parallel implementation.

A large set of corrupted-triple sampling models is integrated from the PyKeen library. The integrated models include TransH, DistMult, HolE, AutoSF, TransF, TorusE, DistMA, ProjE, ConvE, RESCAL, QuatE, TransD, ERMLP, CrossE, TuckER, TransR, PairRE, RotatE, ComplEx, and BoxE [62]. We refer to each of the original papers for the extensive explanation. The parameters used for the evaluation of node embedding models in *GRAPE* pipelines are available in the Supplementary Information S4.1.

9 Visualization of Kipf GCN Models

In the current section, we show visualization based on Keras dot model visualizations of *Kipf GCN* models for node-label, edge-label and edge prediction. Analogous visualizations are available for all other support TensorFlow/Keras models.

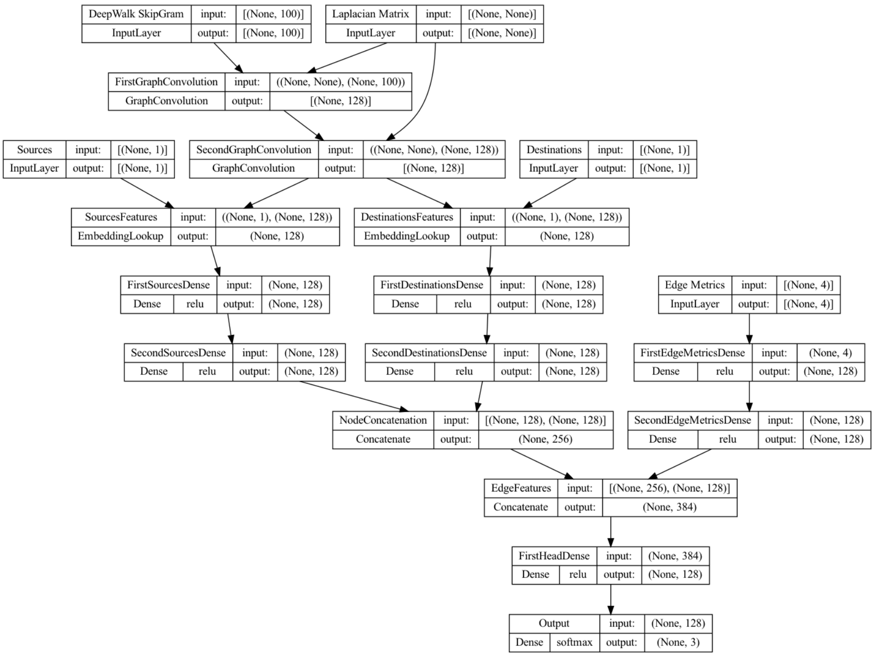

Supplementary Figure 34: Default Kipf GCN model for multiclass edge-label prediction, using also the edge metrics (Jaccard Coefficient, Adamic-Adar, Preferential Attachment, and Resource Allocation Index).

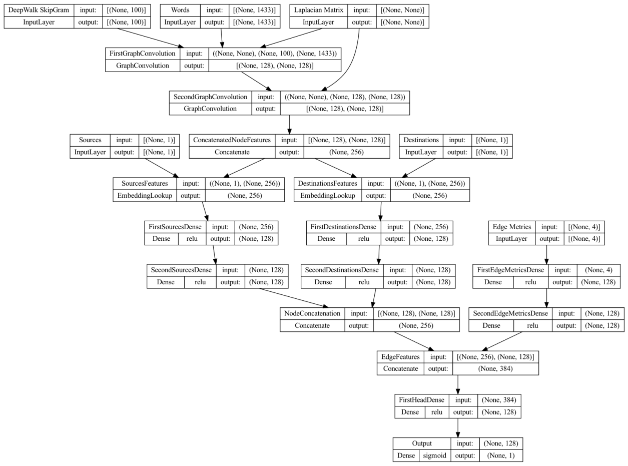

Supplementary Figure 35: Default Kipf GCN model for multi-modal edge prediction, using also the edge metrics (Jaccard Coefficient, Adamic-Adar, Preferential Attachment, and Resource Allocation Index).

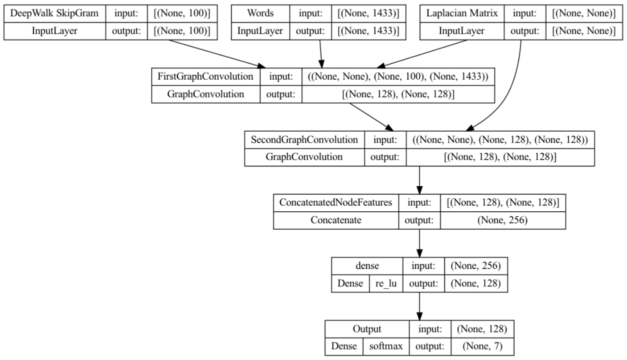

Supplementary Figure 36: Default Kipf GCN model for multi-modal node-label prediction.

10 Other *GRAPE* utilities.

10.1 Graph analysis and graph reporting

GRAPE implements fast algorithms to analyze the overall characteristics of the graph, including Breadth and Depth-first search, Dijkstra, Tarjan's strongly connected components, efficient Diameter computation, spanning arborescence and connected components, approximated vertex cover, triads counting, transitivity, clustering coefficient and triangles counting, Betweenness and stress centrality, Closeness and harmonic centrality, as well as optimized implementations for algebraic set graph-operations and node and edge filters.

10.2 Construction of train and test graphs for edge prediction

To generate train and test samples for the evaluation of edge prediction task, *GRAPE* makes available Kfold, Monte Carlo and Connected Monte Carlo techniques. All methods may be stratified relatively to user-provided edge types. We show examples in Supplementary Figure 37 on a graph with two components.

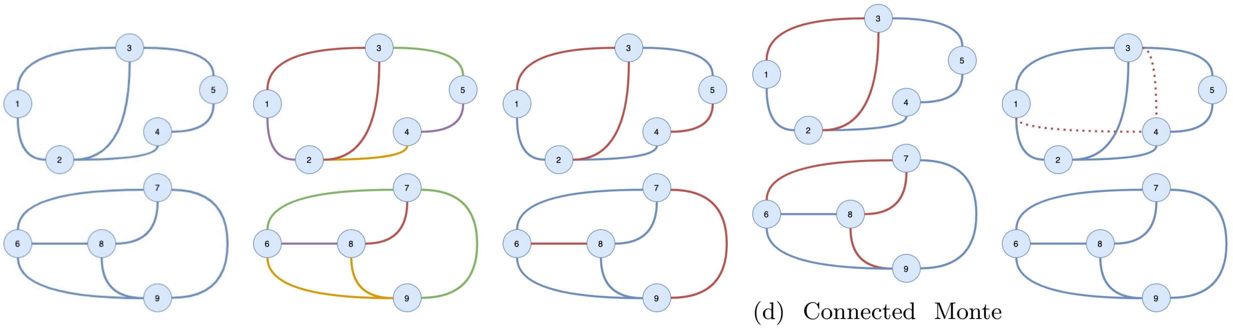

(a) Original graph (b) Kfolds (c) Monte Carlo (d) Connected Monte Carlo (e) Negative edges

Supplementary Figure 37: **Different holdouts on a graph with multiple components:** From left to right, A) the graph considered, composed of two connected components, B) K-folds edge holdouts (we represent each validation fold with a different colour), C) Monte Carlo Holdout, sampling random edges for the validation set (in red) that may generate new connected components and D) Connected Monte Carlo Holdout, sampling random edges for the validation set (in red) without generating new connected components. Finally, in picture E), we show in red as dotted, the generated negative edges.

10.2.1 K-folds

As per the normal Kfolds, this mechanism splits the graph edges into k folds to be used for cross-validating a model on a link prediction task (Supplementary Figure 37b). We suggest using this method when the graph is either composed of a single connected component with high density or, if multiple connected components exist, each component has a high density to avoid creating new components when the procedure randomly removes edges.

10.2.2 Monte Carlo

This method randomly samples the edges, but multiple holdouts may share the same edges (Supplementary Figure 37c). The training graph generated with this method, as for the aforementioned k-folds method, may contain more components than the original graph.

10.2.3 Connected Monte Carlo

Often, in link prediction tasks, it is assumed a closed word hypothesis: that is, components that are not connected do not have unknown edges connecting them. By using the Connected Monte Carlo holdouts guarantees that the training graph always has the same number of connected components of the original graph without creating new ones by reserving a set of edges forming a spanning arborescence for the training set and sampling the test set edges only from the remaining edges (Supplementary Figure 37d). The connected Monte Carlo holdout avoids introducing a negative bias when the task assumes a closed word hypothesis.

For instance, a link prediction model working on the closed word assumption would not predict links between the different components that, for instance, a simple Monte Carlo holdout may generate.

10.2.4 Negative edge sampling

To train edge prediction models, it is common practice to generate negative edges, that is edges that do not exist in the graph. We generate such edges by sampling their vertices from the same connected components, to avoid easily predictable negative edges. Indeed, from our experimental studies we noted that negative edges between two graph connected components have odd embeddings that make it easy to identify them, causing a positive bias in the model performance (Supplementary Figure 37e). To sample massive amounts of source and destination nodes we use a SIMD-vectorized version of xorshift, detailed in the Supplementary Section 7.2.1. Edges may be sampled by either following a uniform, or more preferably, a scale-free distribution. The latter has been shown to introduce less covariate-shift and therefore make the sampled negative edges a meaningful task, while often the uniform sampling can sample trivially false edges.

References

[1] Radim Řehůřek and Petr Sojka. Software Framework for Topic Modelling with Large Corpora. In *Proceedings of the LREC 2010 Workshop on New Challenges for NLP Frameworks*, pages 45–50, Valletta, Malta, May 2010. ELRA. http://is.muni.cz/publication/884893/en.

[2] Siu Kwan Lam, Antoine Pitrou, and Stanley Seibert. Numba: A llvm-based python jit compiler. In *Proceedings of the Second Workshop on the LLVM Compiler Infrastructure in HPC*, pages 1–6, 2015.

[3] Aric Hagberg, Pieter Swart, and Daniel S Chult. Exploring network structure, dynamics, and function using networkx. Technical report, Los Alamos National Lab.(LANL), Los Alamos, NM (United States), 2008.

[4] Chen, Shen Wei. Graphembedding. https://github.com/shenweichen/GraphEmbedding, 2021. Online; accessed 21 July 2021.

[5] Richard A Kronmal and Arthur V Peterson Jr. On the alias method for generating random variables from a discrete distribution. *The American Statistician*, 33(4):214–218, 1979.

[6] Tomas Mikolov, Ilya Sutskever, Kai Chen, Greg Corrado, and Jeff Dean. Distributed representations of words and phrases and their compositionality. In C. J. C. Burges, L. Bottou, M. Welling, Z. Ghahramani, and K. Q. Weinberger, editors, *Advances in Neural Information Processing Systems*, volume 26. Curran Associates, Inc., 2013.

[7] Gabor Csardi and Tamas Nepusz. The igraph software package for complex network research. *InterJournal*, Complex Systems:1695, 2006.

[8] Ranger, Matt. Csrgraph. https://github.com/VHRanger/CSRGraph, 2021. Online; accessed 21 July 2021.

[9] Renming Liu and Arjun Krishnan. Pecanpy: a fast, efficient and parallelized python implementation of node2vec. *Bioinformatics*, 37(19):3377–3379, 2021.

[10] Ryan A. Rossi and Nesreen K. Ahmed. The network data repository with interactive graph analytics and visualization. In *AAAI*, 2015.

[11] Damian Szklarczyk, Annika L Gable, David Lyon, Alexander Junge, Stefan Wyder, Jaime Huerta-Cepas, Milan Simonovic, Nadezhda T Doncheva, John H Morris, Peer Bork, Lars J Jensen, and Christian von Mering. STRING v11: protein–protein association networks with increased coverage, supporting functional discovery in genome-wide experimental datasets. *Nucleic Acids Research*, 47(D1):D607–D613, 2018.

[12] Alan Mislove, Hema Swetha Koppula, Krishna P Gummadi, Peter Druschel, and Bobby Bhattacharjee. Growth of the flickr social network. In *Proceedings of the first workshop on Online social networks*, pages 25–30. ACM, 2008.

[13] Justin T Reese, Deepak Unni, Tiffany J Callahan, Luca Cappelletti, Vida Ravanmehr, Seth Carbon, Kent A Shefchek, Benjamin M Good, James P Balhoff, Tommaso Fontana, et al. Kg-covid-19: a framework to produce customized knowledge graphs for covid-19 response. *Patterns*, 2(1):100155, 2021.

[14] Wouter De Nooy, Andrej Mrvar, and Vladimir Batagelj. *Exploratory social network analysis with Pajek*, 2011.

[15] Katrin Amunts, Claude Lepage, Louis Borgeat, Hartmut Mohlberg, Timo Dickscheid, Marc-Etienne Rousseau, Sebastian Bludau, Pierre-Louis Bazin, Lindsay B. Lewis, Ana-Maria Oros-Peusquens, Nadim J. Shah, Thomas Lippert, Karl Zilles, and Alan C. Evans. Bigbrain: An ultrahigh-resolution 3d human brain model. *Science*, 340(6139):1472–1475, 2013.

[16] Ara Cho, Junha Shin, Sohyun Hwang, Chanyoung Kim, Hongseok Shim, Hyojin Kim, Hanhae Kim, and Insuk Lee. Wormnet v3: a network-assisted hypothesis-generating server for caenorhabditis elegans. *Nucleic acids research*, 42(W1):W76–W82, 2014.

[17] J. Duch and A. Arenas. Community identification using extremal optimization phys. *Rev. E*, 72:027104, 2005.

[18] Kwang-Il Goh, Michael E Cusick, David Valle, Barton Childs, Marc Vidal, and Albert-László Barabási. The human disease network. *Proceedings of the National Academy of Sciences*, 104(21):8685–8690, 2007.

[19] Rohit Singh, Jinbo Xu, and Bonnie Berger. Global alignment of multiple protein interaction networks with application to functional orthology detection. *PNAS*, 105(35):12763–12768, 2008.

[20] Chris Stark, Bobby-Joe Breitkreutz, Teresa Reguly, Lorrie Boucher, Ashton Breitkreutz, and Mike Tyers. Biogrid: a general repository for interaction datasets. *Nucleic acids research*, 34(suppl_1):D535–D539, 2006.

[21] Mukesh Bansal, Vincenzo Belcastro, Alberto Ambesi-Impiombato, and Diego Di Bernardo. How to infer gene networks from expression profiles. *Molecular systems biology*, 3(1), 2007.

[22] Hawoong Jeong, Sean P Mason, A-L Barabási, and Zoltan N Oltvai. Lethality and centrality in protein networks. *Nature*, 411(6833):41–42, 2001.

[23] Renming Liu, Christopher A Mancuso, Anna Yannakopoulos, Kayla A Johnson, and Arjun Krishnan. Supervised learning is an accurate method for network-based gene classification. *Bioinformatics*, 36(11):3457–3465, 2020.

[24] Christopher J Mungall, Julie A McMurry, Sebastian Köhler, James P Balhoff, Charles Borromeo, Matthew Brush, Seth Carbon, Tom Conlin, Nathan Dunn, Mark Engelstad, et al. The monarch initiative: an integrative data and analytic platform connecting phenotypes to genotypes across species. *Nucleic acids research*, 45(D1):D712–D722, 2017.

[25] Lise Getoor. Link-based classification. In *Advanced methods for knowledge discovery from complex data*, pages 189–207. Springer, 2005.

[26] TJ Callahan. Pheknowlator, 2019.

[27] Prithviraj Sen, Galileo Namata, Mustafa Bilgic, Lise Getoor, Brian Galligher, and Tina Eliassi-Rad. Collective classification in network data. *AI magazine*, 29(3):93–93, 2008.

[28] Galileo Namata, Ben London, Lise Getoor, Bert Huang, and UMD EDU. Query-driven active surveying for collective classification. In *10th International Workshop on Mining and Learning with Graphs*, volume 8, 2012.

[29] Radim Řehůřek and Petr Sojka. Software Framework for Topic Modelling with Large Corpora. In *Proceedings of the LREC 2010 Workshop on New Challenges for NLP Frameworks*, pages 45–50, Valletta, Malta, May 2010. ELRA. http://is.muni.cz/publication/884893/en.

[30] Jure Leskovec and Rok Sosič. Snap: A general-purpose network analysis and graph-mining library. *ACM Transactions on Intelligent Systems and Technology (TIST)*, 8(1):1, 2016.

[31] Peter Elias. Efficient storage and retrieval by content and address of static files. *Journal of the ACM (JACM)*, 21(2):246–260, 1974.

[32] Sebastiano Vigna. Quasi-succinct indices. In *Proceedings of the sixth ACM international conference on Web search and data mining*, pages 83–92, 2013.

[33] Peter Elias. On binary representations of monotone sequences. In *Proc. sixth princeton conference on information sciences and systems*, pages 54–57, 1972.

[34] Peter Elias. Universal codeword sets and representations of the integers. *IEEE transactions on information theory*, 21(2):194–203, 1975.

[35] Giulio Ermanno Pibiri and Rossano Venturini. Dynamic elias-fano representation. In *28th Annual symposium on combinatorial pattern matching (CPM 2017)*. Schloss Dagstuhl-Leibniz-Zentrum fuer Informatik, 2017.

[36] Giulio Ermanno Pibiri. *Dynamic Elias-Fano Encoding*. PhD thesis, Master's Thesis, University of Pisa, Pisa, Italy, 2014.

[37] Guy Jacobson. Space-efficient static trees and graphs. In *30th Annual Symposium on Foundations of Computer Science*, pages 549–554. IEEE Computer Society, 1989.

[38] Donald Knuth. Seminumerical algorithms. *The art of computer programming*, 2, 1981.

[39] Aditya Grover and Jure Leskovec. node2vec: Scalable feature learning for networks. In *Proceedings of the 22nd ACM SIGKDD international conference on Knowledge discovery and data mining*, pages 855–864, 2016.

[40] Daniel Lemire, Leonid Boytsov, and Nathan Kurz. Simd compression and the intersection of sorted integers. *Software: Practice and Experience*, 46(6):723–749, 2016.

[41] David Blackman and Sebastiano Vigna. Scrambled linear pseudorandom number generators. *ACM Transactions on Mathematical Software (TOMS)*, 47(4):1–32, 2021.

[42] George Marsaglia. Xorshift rngs. *Journal of Statistical Software, Articles*, 8(14):1–6, 2003.

[43] Jung-Chang Huang and Tau Leng. Generalized loop-unrolling: a method for program speedup. In *Proceedings 1999 IEEE Symposium on Application-Specific Systems and Software Engineering and Technology. ASSET'99 (Cat. No. PR00122)*, pages 244–248. IEEE, 1999.

[44] Michael D Vose. A linear algorithm for generating random numbers with a given distribution. *IEEE Transactions on software engineering*, 17(9):972–975, 1991.

[45] Jason Evans. A scalable concurrent malloc (3) implementation for freebsd. In *Proc. of the bsdcan conference, ottawa, canada*, 2006.

[46] Nicholas Nethercote and Julian Seward. Valgrind: a framework for heavyweight dynamic binary instrumentation. *ACM Sigplan notices*, 42(6):89–100, 2007.

[47] Leo Torres, Kevin S Chan, and Tina Eliassi-Rad. Glee: Geometric laplacian eigenmap embedding. *Journal of Complex Networks*, 8(2):cnaa007, 2020.

[48] Mingdong Ou, Peng Cui, Jian Pei, Ziwei Zhang, and Wenwu Zhu. Asymmetric transitivity preserving graph embedding. In *Proceedings of the 22nd ACM SIGKDD international conference on Knowledge discovery and data mining*, pages 1105–1114, 2016.

[49] Lei Tang and Huan Liu. Relational learning via latent social dimensions. In *Proceedings of the 15th ACM SIGKDD international conference on Knowledge discovery and data mining*, pages 817–826, 2009.

[50] Dennis L Sun and Cedric Fevotte. Alternating direction method of multipliers for non-negative matrix factorization with the beta-divergence. In *2014 IEEE international conference on acoustics, speech and signal processing (ICASSP)*, pages 6201–6205. IEEE, 2014.

[51] Ziwei Zhang, Peng Cui, Haoyang Li, Xiao Wang, and Wenwu Zhu. Billion-scale network embedding with iterative random projection. In *2018 IEEE International Conference on Data Mining (ICDM)*, pages 787–796. IEEE, 2018.

[52] Shaosheng Cao, Wei Lu, and Qiongkai Xu. Grarep: Learning graph representations with global structural information. In *Proceedings of the 24th ACM international on conference on information and knowledge management*, pages 891–900, 2015.

[53] Jiezhong Qiu, Yuxiao Dong, Hao Ma, Jian Li, Kuansan Wang, and Jie Tang. Network embedding as matrix factorization: Unifying deepwalk, line, pte, and node2vec. In *Proceedings of the eleventh ACM international conference on web search and data mining*, pages 459–467, 2018.

[54] Bryan Perozzi, Vivek Kulkarni, Haochen Chen, and Steven Skiena. Don't walk, skip! online learning of multi-scale network embeddings. In *Proceedings of the 2017 IEEE/ACM International Conference on Advances in Social Networks Analysis and Mining 2017*, pages 258–265, 2017.

[55] Jeffrey Pennington, Richard Socher, and Christopher D Manning. Glove: Global vectors for word representation. In *Proceedings of the 2014 conference on empirical methods in natural language processing (EMNLP)*, pages 1532–1543, 2014.

[56] Tomas Mikolov, Kai Chen, Greg Corrado, and Jeff Dean. Efficient estimation of word representations in vector space. In Yoshua Bengio and Yann LeCun, editors, *1st International Conference on Learning Representations, ICLR 2013, Scottsdale, Arizona, USA, May 2-4, 2013, Workshop Track Proceedings*, 2013.

[57] Nesreen K Ahmed, Ryan A Rossi, John Boaz Lee, Theodore L Willke, Rong Zhou, Xiangnan Kong, and Hoda Eldardiry. role2vec: Role-based network embeddings. *Proc. DLG KDD*, pages 1–7, 2019.

[58] Nino Shervashidze, Pascal Schweitzer, Erik Jan Van Leeuwen, Kurt Mehlhorn, and Karsten M Borgwardt. Weisfeiler-lehman graph kernels. *Journal of Machine Learning Research*, 12(9), 2011.

[59] Benedek Rozemberczki, Oliver Kiss, and Rik Sarkar. Karate Club: An API Oriented Open-source Python Framework for Unsupervised Learning on Graphs. In *Proceedings of the 29th ACM International Conference on Information and Knowledge Management (CIKM '20)*, page 3125–3132. ACM, 2020.

[60] Jian Tang, Meng Qu, Mingzhe Wang, Ming Zhang, Jun Yan, and Qiaozhu Mei. Line: Large-scale information network embedding. In *Proceedings of the 24th International Conference on World Wide Web*, pages 1067–1077, 2015.

[61] Denghui Zhang, Manling Li, Yantao Jia, Yuanzhuo Wang, and Xueqi Cheng. Efficient parallel translating embedding for knowledge graphs. In *Proceedings of the International Conference on Web Intelligence*, pages 460–468, 2017.

[62] Mehdi Ali, Max Berrendorf, Charles Tapley Hoyt, Laurent Vermue, Sahand Sharifzadeh, Volker Tresp, and Jens Lehmann. PyKEEN 1.0: A Python Library for Training and Evaluating Knowledge Graph Embeddings. *Journal of Machine Learning Research*, 22(82):1–6, 2021.
