# Supplementary material for: GRAPE for fast and scalable graph processing and random-walk-based embedding
Source: Nat Comput Sci. 2023 Jun 26;3(6):552–68. doi: 10.1038/s43588-023-00465-8 (PMC10768636; doi:10.1038/s43588-023-00465-8)
Supplement: Supplementary file 2 — Reporting summary [file 43588_2023_465_MOESM2_ESM.pdf]

nature portfolio

Corresponding author(s): Giorgio Valentini

Last updated by author(s): May 6, 2023

Reporting Summary

Nature Portfolio wishes to improve the reproducibility of the work that we publish. This form provides structure for consistency and transparency in reporting. For further information on Nature Portfolio policies, see our Editorial Policies and the Editorial Policy Checklist.

Statistics

For all statistical analyses, confirm that the following items are present in the figure legend, table legend, main text, or Methods section.

n/a	Confirmed	
☐	☒	The exact sample size (*n*) for each experimental group/condition, given as a discrete number and unit of measurement
☐	☒	A statement on whether measurements were taken from distinct samples or whether the same sample was measured repeatedly
☐	☒	The statistical test(s) used AND whether they are one- or two-sided<br>*Only common tests should be described solely by name; describe more complex techniques in the Methods section.*
☐	☒	A description of all covariates tested
☒	☐	A description of any assumptions or corrections, such as tests of normality and adjustment for multiple comparisons
☐	☒	A full description of the statistical parameters including central tendency (e.g. means) or other basic estimates (e.g. regression coefficient) AND variation (e.g. standard deviation) or associated estimates of uncertainty (e.g. confidence intervals)
☐	☒	For null hypothesis testing, the test statistic (e.g. *F*, *t*, *r*) with confidence intervals, effect sizes, degrees of freedom and *P* value noted<br>*Give P values as exact values whenever suitable.*
☒	☐	For Bayesian analysis, information on the choice of priors and Markov chain Monte Carlo settings
☒	☐	For hierarchical and complex designs, identification of the appropriate level for tests and full reporting of outcomes
☒	☐	Estimates of effect sizes (e.g. Cohen's *d*, Pearson's *r*), indicating how they were calculated

Our web collection on statistics for biologists contains articles on many of the points above.

Software and code

Policy information about availability of computer code

Data collection

All the graphs considered within the manuscript experiments can be downloaded using the GRAPE software available from our GitHub repository (https://github.com/AnacletoLAB/grape).

Data analysis

In order to perform the data analysis performed in our work we used our GRAPE open source code and also open source code from other publicly available libraries.
In particular we used Python v. 3.7 and Rust v. 1.63.0 and the following open source libraries:
networkx v. 2.8.5
igraph v. 0.9.11
csrgraph v. 0.1.28
pecanpy v.2.0.0 https://github.com/LucaCappelletti94/PecanPy
node2vec v.0.4.5 https://github.com/LucaCappelletti94/node2vec
graphEmbedding v.0.1.0 https://github.com/LucaCappelletti94/GraphEmbedding
nodevectors v.0.1.23 https://github.com/LucaCappelletti94/nodevectors
snap v.6.0 https://github.com/snap-stanford/snap/tree/0b73cda5f0c9f0dcfd47172eea8be26ba414941a
fastnode2vec v. 0.0.6
PyTorch Geometric v.2.2.0 https://github.com/pyg-team/pytorch_geometric
PyKeen v.1.10.1 https://github.com/pykeen/pykeen
Karateclub v.1.3.4 https://github.com/benedekrozemberczki/karateclub
Nodevectors v.0.1.23 https://github.com/VHRanger/nodevectors
All the GRAPE code related to the experiments presented within the manuscript is available from publicly accessible GitHub repositories.
Firstly, GRAPE (v. 0.1.30) can be readily installed from PyPI: https://pypi.org/project/grape.

The source code, reference manual and tutorials for its usage, alongside several application examples, are available on GitHub: https://github.com/AnacletoLAB/grape.
All the scripts to reproduce the experiments showed in the paper are available from GitHub:
a) Ensmallen benchmarks: loading graphs, executing first and second-order random walks: https://github.com/LucaCappelletti94/ensmallen_experiments;
b) Approximated random walk experiments: https://github.com/AnacletoLAB/grape/blob/main/tutorials/Comparing%20DeepWalk%20and%20Node2Vec%20running%20on%20exact%20and%20approximated%20random%20walks.ipynb;
c) Experimental comparison of node embedding methods: (I) Edge prediction experiments: https://github.com/AnacletoLAB/grape/blob/main/tutorials/Using%20the%20edge%20prediction%20pipeline.ipynb, (II) Node-label prediction experiments: https://github.com/AnacletoLAB/grape/blob/main/tutorials/Using%20the%20node-label%20prediction%20pipeline.ipynb;
d) Comparison of GRAPE with state-of-the-art libraries on big real-world graphs: https://github.com/LucaCappelletti94/embiggen_experiments/tree/master/node2vec_comparisons.
The GRAPE software is delivered under the MIT license.

For manuscripts utilizing custom algorithms or software that are central to the research but not yet described in published literature, software must be made available to editors and reviewers. We strongly encourage code deposition in a community repository (e.g. GitHub). See the Nature Portfolio guidelines for submitting code & software for further information.

Data

Policy information about availability of data

All manuscripts must include a data availability statement. This statement should provide the following information, where applicable:

- Accession codes, unique identifiers, or web links for publicly available datasets
- A description of any restrictions on data availability
- For clinical datasets or third party data, please ensure that the statement adheres to our policy

GRAPE graph retrieval includes all the graphs used in the Ensmallen benchmarks and the pipeline experiments and more than 80,000 graphs are available from https://github.com/AnacletoLAB/grape. Graphs used for the Ensmallen benchmarks are detailed in Supplementary Information Section 2. Graphs used for edge and node-label prediction experiments are detailed in Supplementary Information Section 3. The real world graphs used in Section 2.5 are downloadable from https://archive.org/download/ctd_20220404/CTD.tar (Pre-built CTD), https://archive.org/download/pheknowlator_20220411/PheKnowLator.tar (Pre-built biomedical PheKnowLator data), and https://archive.org/download/wikipedia_edge_list.npy/wikipedia_edge_list.npy.gz (Pre-built English Wikipedia). More details are available in Supplementary Information Section 6. The procedures for the construction of train and test graphs for edge prediction are detailed in Supplementary Information Section 10.2. Source Data for Figures 2, 3, 4 and 5 are available with this manuscript

Human research participants

Policy information about studies involving human research participants and Sex and Gender in Research.

Reporting on sex and gender	NA
Population characteristics	NA
Recruitment	NA
Ethics oversight	NA

Note that full information on the approval of the study protocol must also be provided in the manuscript.

Field-specific reporting

Please select the one below that is the best fit for your research. If you are not sure, read the appropriate sections before making your selection.

☒ Life sciences ☐ Behavioural & social sciences ☐ Ecological, evolutionary & environmental sciences

For a reference copy of the document with all sections, see nature.com/documents/nr-reporting-summary-flat.pdf

Life sciences study design

All studies must disclose on these points even when the disclosure is negative.

Sample size

Our resource sw can be applied not only to biological data, but to any data that can be represented by a graph. From this standpoint our porposed sw resource can be applied not only to Life Science studies, Behavorial and social sciences and Ecological and environmental sciences, but to any discipline where the corresponding data can be represented through a graph. In our experiments we used data from different public repositories (including biological data too) and we did not study the sample size since in most cases we analyzed very big graphs, characterized by a high number of nodes and huge number of edges.
We included 44 different data sets for comparing the empirical computational time in classical graph processing tasks across different state-of-the-art graph libraries. The size of each graph varies from a few hundreds to billions of edges in order to evaluate the scalability of software libraries. To our knowledge this is one of the largest esperimental comparison performed to evaluate the performance of graph processing software libraries. We then analyzed in detail the performance of state of the art graph processing libraries on three large real-worlds graphs.

	We selected the CTD and Wikipedia graphs since they are two wll-known big graphs largely used by the scientific community. Moreover we used also a biomedical Knowledge Graph generated through the tool PheKnowLator, in order to provide an experimental comparison on a significant and large biomedical graph to show the effectiveness of GRAPE in processing and analyzing big biomedical graphs that are interesting for the biomedcial community.
Data exclusions	We did not exclude any data from our experiments. We only used the GRAPE sw to remove duplicated data in the analyzed repository.
Replication	GRAPE has been designed to make easily reproducible all the results, also comparing results obtained with different sw resources and libraries, by using the standardized pipelines available in GRAPE. All the machine learning experiments described in the paper can be successfully reproduced using the scripts available from the GRAPE GitHub repository.<br>In particular experimental comparisons between the different libraries and methods have been repeated from 10 to 30 times, depending on the different computational experiments performed, using multiple hold-out techniques. The results obtained by the GRAPE library are robust and stable as witnessed by the low standard deviation of the measured accuracy on the different test sets generated in the repeated experiments.
Randomization	Examples were always randomly assigned to training and test samples, using multiple Montecarlo hold-out techniques in order to obtain statistically robust results. GRAPE offers sw resources to perform these randomization steps in a fully automated way in the context of graph-structured data.
Blinding	In our experiments we used publicly available data in graph format where labels associated with nodes (when available) are public. However our experimental procedures to assign samples to groups were completely randomized and in our supervised or semi-supervised prediction tasks always the labels of the test set have been not used for training. Hence from this standpoint our experiments were blind. However, the main aim of our work was not to provide novel experimental results in Life Sciences (or in any other field), but to provide a sw resource that can be robustly applied to the analysis of graphs to obtain reproducible results and that can scale efficiently with big graphs obtaining at the same time prediction results comparable with state of the art methods.

Reporting for specific materials, systems and methods

We require information from authors about some types of materials, experimental systems and methods used in many studies. Here, indicate whether each material, system or method listed is relevant to your study. If you are not sure if a list item applies to your research, read the appropriate section before selecting a response.

Materials & experimental systems

n/a	Involved in the study
☒	☐ Antibodies
☒	☐ Eukaryotic cell lines
☒	☐ Palaeontology and archaeology
☒	☐ Animals and other organisms
☒	☐ Clinical data
☒	☐ Dual use research of concern

Methods

n/a	Involved in the study
☒	☐ ChIP-seq
☒	☐ Flow cytometry
☒	☐ MRI-based neuroimaging
